# *cis*-Selective Acyclic Diene Metathesis Polymerization of **α**,**ω**-Dienes

**DOI:** 10.1021/jacs.3c03978

**Published:** 2023-05-31

**Authors:** Samuel J. Kempel, Ting-Wei Hsu, Jake L. Nicholson, Quentin Michaudel

**Affiliations:** † Department of Chemistry, Texas A&M University, College Station, Texas 77843, United States; § Department of Materials Science & Engineering, Texas A&M University, College Station, Texas 77843, United States

## Abstract

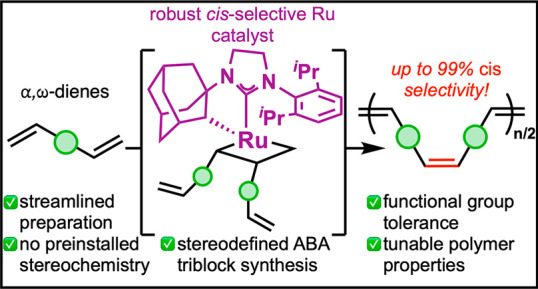

The *cis*/*trans* stereochemistry of repeating alkenes in polymers provides a powerful handle to modulate the thermal and mechanical properties of these soft materials, but synthetic methods to precisely dictate this parameter remain scarce. We report herein a *cis*-selective acyclic diene metathesis (ADMET) polymerization of readily available α,ω-diene monomers with high functional group tolerance. Identification of a highly stereoselective cyclometalated Ru catalyst allowed the synthesis of a broad array of polymers with *cis* contents up to 99%. This platform was leveraged to study the impact of the *cis* geometry on the thermal and mechanical properties of polyalkenamers, including an ABA triblock copolymer synthesized via extension of a *cis*-rich telechelic polyoctenamer with d,l-lactide. These results suggest that *cis*-selective ADMET affords an efficient strategy to tune the properties of a variety of polymers.

The development of stereoselective methods to access olefin-containing macromolecules with precise geometries remains a grand synthetic challenge despite the documented dependence of the properties of such soft materials on *cis*/*trans* stereochemistry.^1^ For example, *cis*-polyisoprene (PI) is an elastic soft material, while *trans*-PI is a hard, brittle material.^2^ Homogeneous and heterogeneous catalysts have been developed for the coordination–insertion polymerization of 1,3-dienes with selective formation of either *trans* or *cis* linkages, but these catalytic systems are notoriously intolerant to polar functional groups and can lead to the formation of vinyl defects through competitive 1,2-insertions.^3^ Recently, several elegant approaches have been implemented to deliver polymers with predictable *cis*/*trans* contents either through thiol–yne click chemistry^4−6^ or via a metal-free ring-opening metathesis polymerization (ROMP) mediated by light.^7^ However, the scope of these processes is limited, and high *cis* contents are generally more challenging to access because of thermodynamic penalties. Monomers containing a spectator *cis*-olefin have been used to circumvent this issue,^8−10^ but undesired isomerization can erode the stereochemistry of the macromolecules.^11,12^

Polymerizations based on olefin metathesis, such as acyclic diene metathesis (ADMET)^13,14^ and ROMP,^15^ represent a promising and versatile strategy to access a diverse pool of stereodefined polyalkenamers because of the robustness, functional-group tolerance, and structural diversity of metathesis catalysts.^16,17^ Specifically designed W or Mo alkylidenes were found to overcome the thermodynamic preference of ROMP and to deliver high *cis* selectivity mostly with nonpolar monomers via kinetic control.^18,19^ The recent development of *Z*-selective^20−25^ and stereoretentive^26−28^ Ru catalysts has allowed the expansion of the scope of *cis*-selective ROMP processes.^29−32^ Interestingly, while ADMET is a powerful tool for the precise synthesis of polymers,^33,34^ control over the stereochemistry of the repeating alkenes has long escaped this versatile polymerization.

ADMET typically delivers polymers with a predominance of *trans* alkenes with Grubbs and Hoveyda–Grubbs dichloro Ru catalysts (Scheme 1a).^13,14^ We recently leveraged the exquisite stereoretention afforded by dithiolate Ru catalysts to produce a variety of all-*cis* polyalkenamers using *cis*,*cis*-diene monomers (Scheme 1b).^35^ However, this method required the synthesis of monomers with preinstalled *cis*,*cis* stereochemistry and utilized air-sensitive Ru carbenes.^36^

**Scheme 1:**
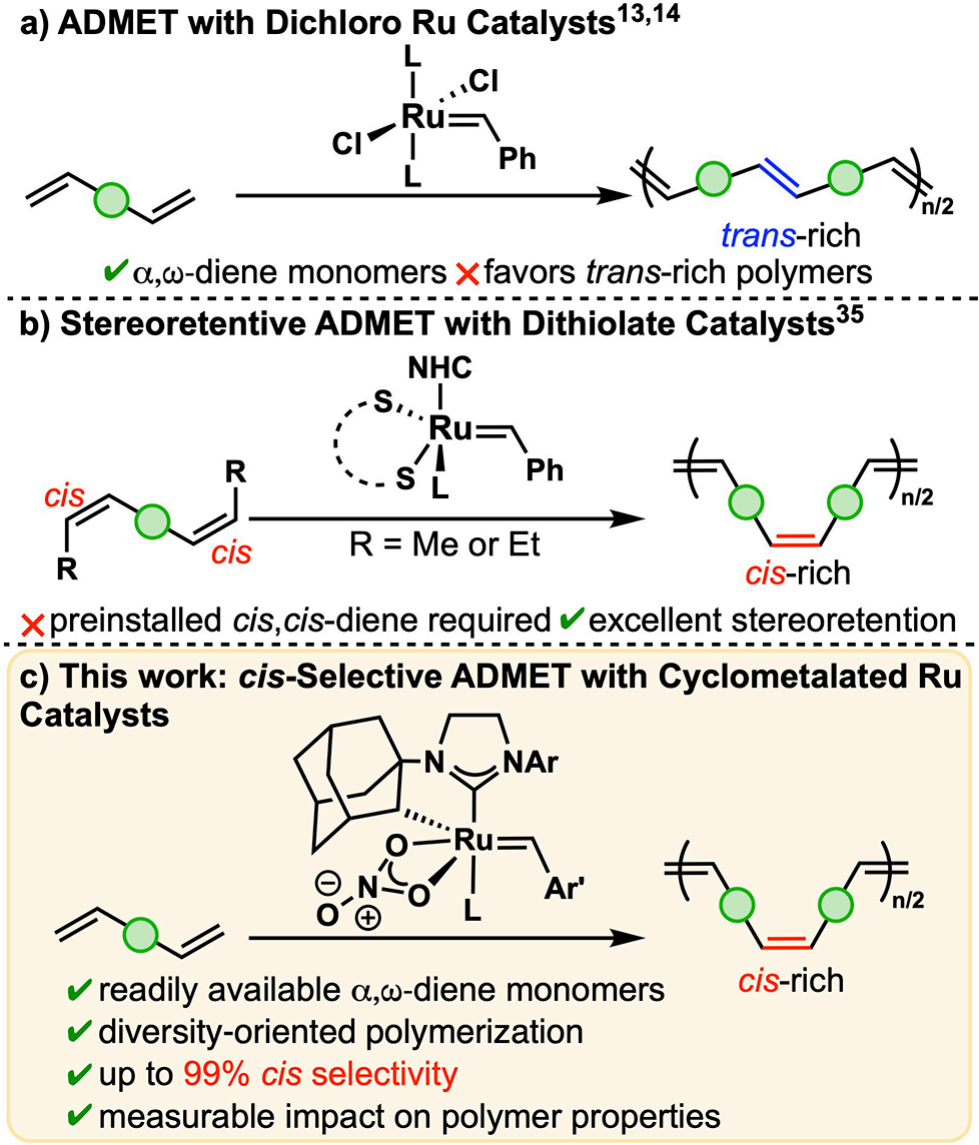
Typical ADMET versus Stereoretentive and *cis*-Selective Processes

Herein, we report a *cis*-selective ADMET process capitalizing on robust cyclometalated Ru–carbenes (Scheme 1c), which afforded a broad array of polyalkenamers with *cis* content up to >99% from readily accessible α,ω-dienes containing various functional groups. This diversity-oriented polymerization allowed the study of the influence of olefin stereochemistry over the thermal properties of these materials. Finally, an ABA triblock copolymer was prepared to demonstrate that mechanical properties can be modulated through modification of the stereochemistry of the middle block.

ADMET is a polycondensation involving iterative cross-metathesis reactions between α,ω-dienes (Scheme 2). To drive this fully reversible process toward high molar masses, continuous removal of ethylene is required. We hypothesized that using catalysts allowing kinetic control, including cyclometalated **Ru-3**(21,37) or dithiolate **Ru-4**(38) (Table 1), would thwart the thermodynamic selectivity and lead to a *cis*-selective ADMET if a robust catalyst capable of maintaining high *cis* selectivity over time could be identified. Carbonate monomer **1a** was selected at the onset of the investigation to favor ADMET over the competing ring-closing metathesis. As a benchmark, monomer **1a** was exposed to typical ADMET conditions using dichloro **Ru-1** and **Ru-2**. Upon reaction with **Ru-1** at 80 °C in 1,2,4-trichlorobenzene (TCB) under vacuum (100 mTorr), polymer **P1a** was formed with only 14% *cis* double bonds (Table 1, entry 1). Polymerization with **Ru-2** delivered **P1a** with a similarly low *cis* content (9%) (Table 1, entry 2). Surprisingly, commercially available *cis*-selective catalyst **Ru-3a** only marginally improved the *cis* content to 18% (Table 1, entry 3). On the basis of the unique geometry of the ruthenacycle imparted by the nitrato and adamantane ligand,^24,39^ we hypothesized that increasing the steric hindrance of the aryl substituent of the *N*-heterocyclic carbene (NHC) (DIPP vs Mes) might improve the stereoselectivity. Pleasingly, switching to **Ru-3b**, which was first reported by Grubbs and coworkers,^23^ more than doubled the *cis* selectivity to 38% (Table 1, entry 4). To further favor kinetic control and minimize potential unselective secondary metathesis events, the reaction temperature was lowered to 40 °C, which led to 97% *cis* content (Table 1, entry 5). Decreasing the temperature further to 23 °C led to the isolation of an all-*cis***P1a** (>99% *cis*) within the limit of detection of ^1^H NMR (Table 1, entry 6). While a decrease in molar masses was observed at lower temperature, respectable degrees of polymerization (DP = ∼50–70) and molar masses (*M*_n_ = 9.8 and 13.5 kg/mol) were obtained for **P1a** exhibiting 97–99% *cis*-alkenes (Table 1, entries 5 and 6).

**Scheme 2:**
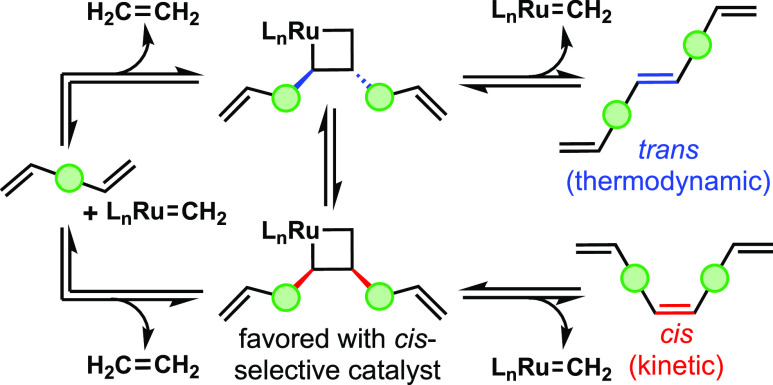
Development of a *cis-*Selective ADMET Process through Kinetic Control

**Table 1:**
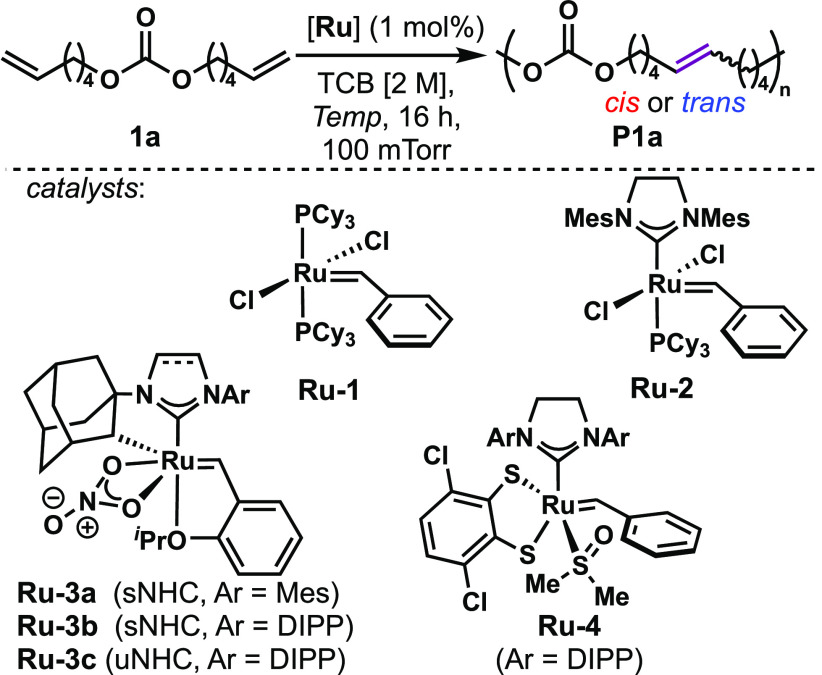
Optimization of *cis*-Selective ADMET^a^

entry	catalyst	*T* (°C)	*M*_n_ (kg/mol)^b^	*Đ*	*cis* (%)^c^
**1**	**Ru-1**	80	27.9	1.75	14
**2**	**Ru-2**	80	25.6	1.89	9
**3**	**Ru-3a**	80	17.7	1.73	18
**4**	**Ru-3b**	80	28.7	2.99	38
**5**	**Ru-3b**	40	13.5	1.75	97
**6**	**Ru-3b**	23	9.8	1.67	99
**7**^d^	**Ru-3b**	23	9.9	1.47	99
**8**^d^	**Ru-3a**	23	11.2	1.61	80
**9**^d^	**Ru-3c**	23	6.8	1.52	89
**10**^d^,^e^	**Ru-4**	23	—	—	—

a sNHC = saturated NHC; uNHC = unsaturated NHC; DIPP = 2,6-diisopropylphenyl; Mes = 2,4,6-trimethylphenyl.

b Determined through size exclusion chromatography (SEC) in THF against polystyrene standards.

c Determined via ^1^H NMR analysis.

d Reaction performed at a concentration of 5 M.

e Reacted for 4 h instead of 16 h.

Performing the polymerization at higher concentration (*C* = 5 M) did not increase *M*_n_ (Table 1, entry 7), while attempts to run the reaction in the bulk only delivered small oligomers (Table S1). The importance of the DIPP substituent on *cis* selectivity was further demonstrated by using **Ru-3a** in the optimal reaction conditions, which resulted in only 80% *cis*-**P1a** (Table 1, entry 8). Unsaturated variant **Ru-3c**,^37^ showcased slightly lower stereoselectivity (89% *cis*) and molar masses (6.8 kg/mol) (Table 1, entry 9). Finally, stereoretentive catalyst **Ru-4** led to unproductive ADMET presumably because of the rapid degradation of the unstable dithiolate Ru methylidene intermediate (Table 1, entry 10).^35,36^ Further investigation into the solvent concentration, temperature, time, and catalyst loading did not produce polymers with higher molar masses (Table S1).

With these optimized conditions in hand, we investigated the scope of the polymerization (Table 2). Polycarbonate **P1a**, polysulfite **P2a**, and polyether **P4a** were all isolated with 99% *cis* content, while polyester **P3a** was formed with slightly diminished *cis* content (91% *cis*). Reducing the number of methylene spacers between the alkene and the functional group did not negatively affect the stereoselectivity (**P1b–4b**). Commercially available deca-1,9-diene (**5**) was transformed into all-*cis* polyoctenamer **P5**,^40^ the *cis* variant of industrially produced vestenamer.^41^ To further explore the functional group tolerance of the *cis*-selective ADMET, polysiloxanes, which are common in coatings, ceramics, and dynamic covalent networks,^42,43^ were targeted. Polysiloxanes **P6a** and **P6b** were isolated from monomers **6a** and **6b** with exquisite *cis* selectivity and *M*_n_ values up to 17.6 kg/mol. Halogenated monomers **7a,b** and **8a,b** were tolerated, albeit with a slight decrease in *cis* selectivity, which is nonetheless in stark contrast to the typical ADMET polymerization of **7a,b** and **8a,b** with dichloro Ru carbenes.^44^ These halogenated polymers might be amenable to postpolymerization functionalization for the precise synthesis of additional polymer classes.^45,46^ Interestingly, alcohol monomers **9a** and **9b** were polymerized with high *cis* selectivity (93 and 96%) but in lower molar masses, which was ascribed to potential poisoning of the Ru catalyst. Overall, all monomers could be purchased or synthesized without tedious purifications, which is a notable advantage of the *cis*-selective ADMET. Additionally, **Ru-3b** was not found to be sensitive to oxidative degradation in contrast to dithiolate Ru catalysts (e.g., **Ru-4**, Table S2).^47^

**Table 2:**
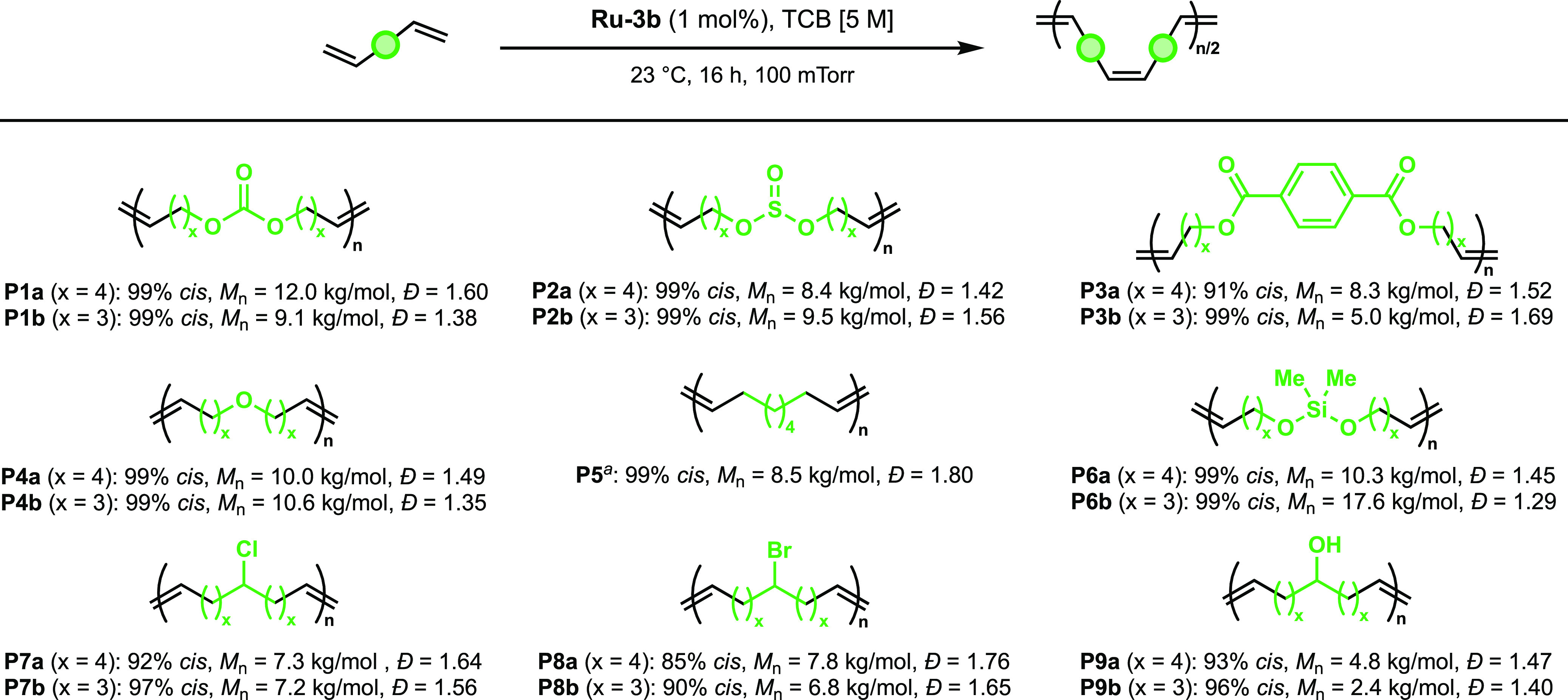
Substrate Scope for *cis*-Selective ADMET

a N_2_ purging instead of vacuum; *C* = 2 M instead of 5 M.

The development of a versatile *cis*-selective ADMET allowed us to probe the impact of *cis*/*trans* stereochemistry over the thermal properties of about 30 polyalkenamers. *trans*-Rich variants of **P1**–**P8** (>70% *trans*) were synthesized using **Ru-1** (Supporting Information) and compared with the *cis*-rich polymers synthesized with **Ru-3b**. The thermal stability of the polymers was tested through thermogravimetric analysis (TGA, Figure 1a). Interestingly, *cis*-rich polyalkenamers were found to have higher decomposition temperature (*T*_d_) values in almost all cases, with the exception of **P5** and **P6a**. The increased thermal stability is especially marked for polyesters (Δ*T*_d_ = 36 °C for **P3b**) and polycarbonates (Δ*T*_d_ = 36 °C for **P1a** and 61 °C for **P1b**). The thermal properties were further investigated through differential scanning calorimetry (DSC). A general trend was also observed for the glass-transition temperature (*T*_g_) values. All polyalkenamers with an observable *T*_g_ within the scanned temperature range exhibited a lower *T*_g_ for the *cis* congener relative to the *trans* one (Figure 1b). Finally, only a few polyalkenamers presented a melting transition (Figure 1c). While **P5** was characterized by a melting temperature (*T*_m_) in both *cis*-rich (*T*_m_ = 32 °C) and *trans*-rich forms (*T*_m_ = 24 °C), **P1a**, **P3a**, **P4a**, and **P4b** only had a *T*_m_ when *trans* linkages were predominant throughout the backbone. Semicrystallinity is known to increase with the *trans* content in poly(1,3-diene)s, but inconsistent trends have been observed between stereochemistry and semicrystallinity with other families, including polycarbonates^8,35^ and polynorbornene.^7,48^ ADMET offers a unique opportunity to generate libraries of both *trans*-rich and *cis*-rich polyalkenamers upon the choice of catalyst and to interrogate the complex relationship between precise molecular structure and material properties.

**Figure 1:**
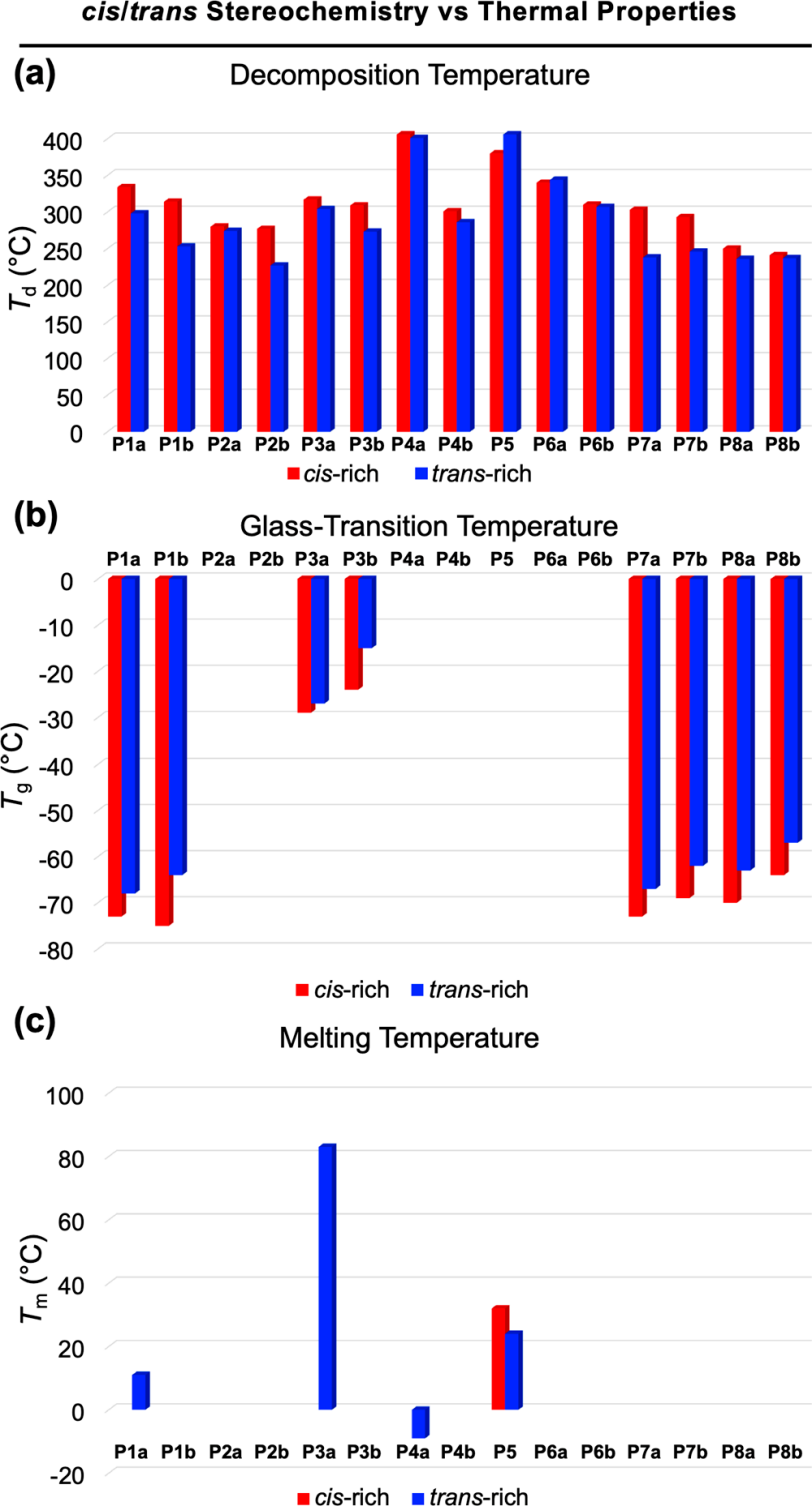
Bar graphs comparing the influence of stereochemistry on (a) *T*_d_ (5% weight loss), (b) *T*_g_, and (c) *T*_m_.

Polyalkenamers are commonly incorporated into industrial block copolymers to obtain thermoplastic elastomers (TPE),^1^ but very few studies have investigated the effect of *cis*/*trans* configuration.^49^ We sought to build an ABA triblock copolymer using **P5** as the middle block and poly(lactic acid) (PLA) as the end blocks. As a biosourced and biodegradable polymer, PLA is an attractive, yet brittle, material^50^ whose toughness can be improved by incorporation of a rubbery middle block.^51^ Building upon a polymerization–depolymerization ADMET strategy initially reported by Wagener and coworkers with **Ru-1**,^52^ telechelic **P5**_***cis***_**-OAc** was synthesized using monomer **5** in the presence of **Ru-3b** and acetate reagent **10** (Figure 2a). Optimized conditions provided quantitative capping of both chain ends, as shown by NMR. Subsequent basic hydrolysis cleanly delivered macromolecular diol **P5**_***cis***_**-OH** (*M*_n_ = 3.3 kg/mol, 99% *cis*) with no change in *M*_n_ compared with the acetoxy precursor. Meanwhile, **P5**_***trans***_**-OH** (*M*_n_ = 3.1 kg/mol, 89% *trans*) was prepared similarly using **Ru-1**. Chain extension of telechelic macroinitiators **P5**_***cis***_**-OH** and **P5**_***trans***_**-OH** using d,l-lactide (**11**) and triazabicyclodecene (TBD) as catalyst efficiently provided ABA triblock copolymers **P11**-*b*-**P5**-*b*-**P11** with either a *cis*- or *trans-*rich middle **P5** block but similar molar mass distributions. TGA and DSC analysis revealed interesting trends (Figure 2b). Both triblocks displayed higher *T*_d_ values than that of homopolymer **P11**, with the *cis* triblock being highest (278 °C), which is consistent with our previous observations. Incorporation of a *cis* middle block also led to the starkest decrease in *T*_g_ (32 vs 44 °C for the *trans* and 49 °C for **P11**). Finally, only the *trans* triblock showcased crystallinity, which is in line with previous literature reports.^51^ Nanoindentation was subsequently used to determine the hardness (Figure S45) and reduced Young’s modulus (*E*_r_) of all three polymers from the unloading segments of the load–displacement curves (Figure S44) using the standard Oliver and Pharr analysis.^53^ As expected on the basis of prior studies,^51^ both triblock architectures had a decreased *E*_r_ compared with **P11** (4.7 GPa, Figure 2c). The *cis* triblock exhibited a lower *E*_r_ (3.0 GPa) than the *trans* congener (3.4 GPa), which indicates that the stiffness of the rubbery block can be finely tuned as a function of its stereochemistry.

**Figure 2:**
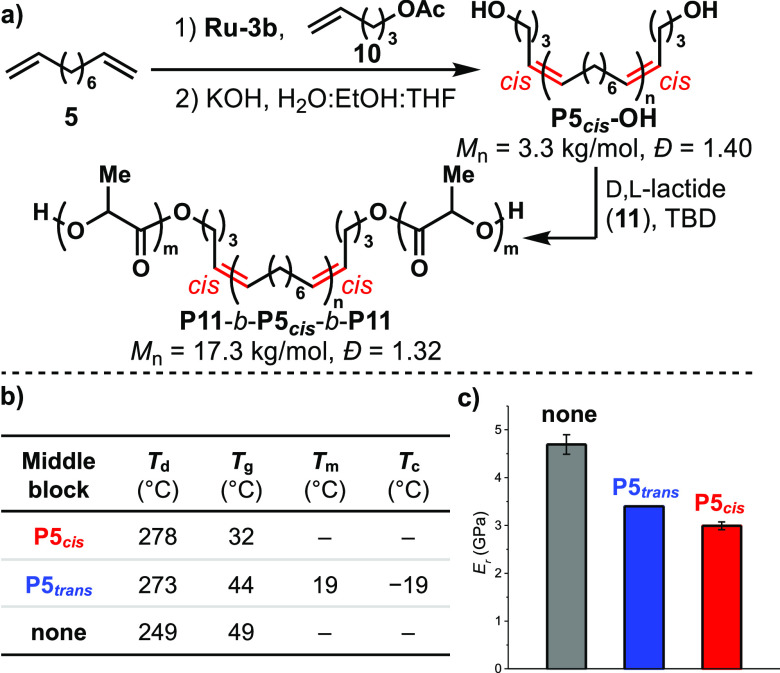
(a) Synthesis of *cis* triblock copolymer **P11**-*b*-**P5**_***cis***_-*b*-**P11**. (b) Thermal properties and (c) reduced Young’s modulus (*E*_r_) of *cis*/*trans* triblocks and homopolymer **P11**.

In summary, we have developed a *cis*-selective ADMET polymerization of readily available and inexpensive α,ω-dienes. Up to 99% *cis* selectivity was obtained for most monomers through exquisite kinetic control of the olefin metathesis process enabled by a robust cyclometalated Ru catalyst (**Ru-3b**) at room temperature. This diversity-oriented polymerization allowed us to compare the thermal properties of a variety of *cis*-rich polyalkenamers containing different polar functional groups with their *trans-*rich congeners. High *cis* content was found to correlate with increased thermal stability, a lower glass-transition temperature, and typically amorphous behavior. Moreover, an ABA triblock copolymer with PLA as end blocks and polyoctenamer as a rubbery middle segment was synthesized. Nanoindentation measurements revealed that the *cis* stereochemistry led to a greater decrease in stiffness when compared with the *trans* triblock. Overall, this study provides both insights in stereoselective catalysis for polymerization and a general method for the modulation of thermal and mechanical properties of soft materials, including TPE, via control of the *cis*/*trans* stereochemistry throughout the main chain.

## Supporting Information Available

Detailed experimental procedures, spectroscopic characterization, and additional supporting data (PDF)
