# Supplementary material for: cis-Selective Acyclic Diene Metathesis Polymerization of α,ω-Dienes
Source: J Am Chem Soc. 2023 May 31;145(23):12459–64. doi: 10.1021/jacs.3c03978 (PMC10330887; doi:10.1021/jacs.3c03978)
Supplement: Supplementary file 1 — ja3c03978_si_001.pdf [file ja3c03978_si_001.pdf]

Supporting Information

cis-Selective Acyclic Diene Metathesis Polymerization of α,ω-Dienes

Samuel J. Kempel,[‡1] Ting-Wei Hsu,[‡1] Jake L. Nicholson,[1] and Quentin Michaudel*[1,2]

[1]*Department of Chemistry, Texas A&M University, College Station, Texas 77843, United States*

[2]*Department of Materials Science and Engineering, Texas A&M University, College Station, Texas 77843, United States*

*Corresponding Author: quentin.michaudel@chem.tamu.edu

‡These authors contributed equally

Experimental Procedures

General Reagent Information S4

General Analytical Information S5

Monomer Synthesis S6

Monomer **1a** S6

Monomer **2a** S7

Monomer **3a** S7

Compound **S1** S8

Monomer **4a** S9

General Siloxane Monomer Procedure S9

General Halogen Monomer Procedure S10

General Alcohol Monomer Procedure S12

Polymerization Procedures S14

Optimization of the cis-Selective ADMET S14

Figure S1. Typical ADMET reaction setup S14

Table S1. Polymerization Results for **P1a** S15

Table S2. Effect of Oxygen on Stereoretentive (**Ru-4**) and *cis*-Selective ADMET (**Ru-3b**). S16

General *cis*-Selective Polymerization Procedure Using Vacuum (100 mTorr) S16

Table S3. Polymerization Results for **P1a** S17

Table S4. Polymerization Results for **P1b** S18

Table S5. Polymerization Results for **P2a**. S18
Table S6. Polymerization Results for **P2b**. S19
Table S7. Polymerization Results for **P3a** S20
Table S8. Polymerization Results for **P3b** S20
Table S9. Polymerization Results for **P4a** S21
Table S10. Polymerization Results for **P4b** S22
Table S11. Polymerization Results for **P6a** S22
Figure S2. ^{1}H NMR (400 MHz) of dec-5-ene-1,10-diol obtained from hydrolysis of *cis* **P6a**........ S23
Table S12. Polymerization Results for **P6b** S24
Figure S3. ^{1}H NMR (400 MHz) of dec-5-ene-1,10-diol obtained from hydrolysis of *cis* **P6b** S25
Table S13. Polymerization Results for **P7a** S25
Table S14. Polymerization Results for **P7b** S26
Table S15. Polymerization Results for **P8a** S27
Table S16. Polymerization Results for **P8b** S27
Table S17. Polymerization Results for **P9a** S28
Table S18. Polymerization Results for **P9b** S29
***cis*-Selective ADMET Polymerization Procedure Using an N_2 Flow for P5** S30
Figure S4. Purging reaction setup for polymer synthesis with **Ru-3b**........ S30
Table S19. Polymerization Results for **P5** S31
Triblock Copolymer Synthesis S31
Figure S5. SEC traces of **P11**-*b*-**P5**-*b*-**P11** S34
Deconvolution Explanation S34
Figure S6. ^{1}H NMR deconvolution of *cis*/*trans* **P1a** S35
Thermal Characterization........ S35
Table S20. Summary of thermal data........ S35
Figure S7. TGA thermograms of **P1a**........ S37
Figure S8. DSC thermograms of **P1a** S38
Figure S9. TGA thermograms of **P1b**........ S38
Figure S10. DSC thermograms of **P1b** S39

Figure S11. TGA thermograms of **P2a** S39
Figure S12. DSC thermograms of **P2a** S40
Figure S13. TGA thermograms of **P2b** S40
Figure S14. DSC thermograms of **P2b** S41
Figure S15. TGA thermograms of **P3a** S41
Figure S16. DSC thermograms of **P3a** S42
Figure S17. TGA thermograms of **P3b** S42
Figure S18. DSC thermograms of **P3b** S43
Figure S19. TGA thermograms of **P4a** S43
Figure S20. DSC thermograms of **P4a** S44
Figure S21. TGA thermograms of **P4b** S44
Figure S22. DSC thermograms of **P4b** S45
Figure S23. TGA thermograms of **P5** S45
Figure S24. DSC thermograms of **P5** S46
Figure S25. TGA thermograms of **P5-OAc** S46
Figure S26. DSC thermograms of **P5-OAc** S47
Figure S27. TGA thermograms of **P5-OH** S47
Figure S28. DSC thermograms of **P5-OH** S48
Figure S29. TGA thermograms of **P11**-*b*-**P5**-*b*-**P11** S48
Figure S30. DSC thermograms of **P11**-*b*-**P5**-*b*-**P11** S49
Figure S31. TGA thermograms of **P6a** S49
Figure S32. DSC thermograms of **P6a** S50
Figure S33. TGA thermograms of **P6b** S50
Figure S34. DSC thermograms of **P6b** S51
Figure S35. TGA thermograms of **P7a** S51
Figure S36. DSC thermograms of **P7a** S52
Figure S37. TGA thermograms of **P7b** S52
Figure S38. DSC thermograms of **P7b** S53
Figure S39. TGA thermograms of **P8a** S53
Figure S40. DSC thermograms of **P8a** S54
Figure S41. TGA thermograms of **P8b** S54

Figure S42. DSC thermograms of **P8b** S55
Figure S43. TGA and DSC thermograms of **P11** S55
Nanoindentation Data S56
Figure S44. Load Displacement S56
Figure S45. Hardness and Reduced Young's Modulus Data S56
^{1}H and ^{13}C NMR Data S57
References S110

General Reagent Information

All reactions were carried out under an inert nitrogen atmosphere with dry solvents under anhydrous conditions unless otherwise stated. Dry dichloromethane (DCM), tetrahydrofuran (THF), *N*,*N*-dimethylformamide (DMF) and toluene (PhMe) were obtained by passing the previously degassed solvents through activated alumina columns. Anhydrous 1,2,4-trichlorobenzene (TCB) was purchased from Sigma Aldrich and used without further purification and degassed via "freeze-pump-thaw" before being brought into a nitrogen-filled glove box. All polymerizations using ruthenium catalysts were set up in a nitrogen-filled glove box (SG1800/750TS-F, VIGOR). Reagents were purchased at the highest commercial quality and used without further purification, unless otherwise stated. For example, Hex-5-en-1-ol (97%), 6-bromohex-1-ene (97%), deca-1,9-diene (**5**) (98%) and 5-bromopent-1-ene (98%) were purchased from Oakwood Chemical and Pent-4-en-1-ol (97%) and TBD (97%) were purchased from Combi-blocks. D,L-Lactide (97%) was purchased from Combi-blocks and recrystallized from ethyl acetate prior to use. Monomers **1b**, **2b**, **3b**, and **4b** were prepared following our previously published procedures.[1] Catalysts **Ru-1**, **Ru-2**, and **Ru-3a** were purchased from Sigma-Aldrich and used without further purification. Catalyst **Ru-3b** and **Ru-4** were generously donated by Umicore. Catalyst **Ru-3c** was prepared following a literature procedure.[2] Yields refer to chromatographically and spectroscopically (^{1}H NMR) homogeneous material, unless otherwise stated. Reactions were monitored by thin layer chromatography (TLC) carried out on 250 μm SiliCycle SiliaPlate™ silica plates (F254), using UV light as the visualizing agent and a solution of $KMnO_4$ and heat as a developing agent. Flash silica gel chromatography was performed using SiliCycle SiliaFlash® Irregular Silica Gel (60 Å, particle size 40–63 μm). Polymers were isolated after precipitation using an Eppendorf 5804 centrifuge.

General Analytical Information

All polymer samples were analyzed using a Tosoh EcoSec HLC 8320GPC system with a TSKgel SuperHM-M column and a TSKgel SuperH-RC column at a flow rate of 0.40 mL/min at 40 °C. THF stabilized with BHT was used as the eluent and all number-average molecular weights (M_n), weight-average molecular weights (M_w), and dispersities ($Đ$) for polymers were calculated from refractive index and UV chromatograms against TSKgel polystyrene standards. NMR spectra were recorded on Bruker Avance Neo 400 or Bruker Avance 500 instruments and were calibrated using residual undeuterated solvent as an internal reference ($CHCl_3$ @ 7.26 ppm ^{1}H-NMR, 77.16 ppm ^{13}C-NMR). The following abbreviations were used to explain NMR peak multiplicities: s = singlet, d = doublet, dd = doublet of doublets, t = triplet, q = quartet, p = pentet, m = multiplet, br = broad. Thermogravimetric analysis (TGA) was performed on a TA Instruments TGA 5500 Thermogravimetric Analyzer. Differential scanning calorimetry (DSC) was performed using a TA instrument DSC 2500. High-resolution mass spectra (HRMS) were recorded on an Agilent LC/MSD TOF mass spectrometer or on an Orbitrap Velos Pro (Thermo Fisher Scientific) mass spectrometer by Atmospheric pressure chemical ionization (APCI) or electrospray ionization (ESI). Nanoindentation experiments were performed on a Hysitron TI 950 Triboindenter equipped with a diamond Berkovich indenter tip. The tip shape was calibrated to a fused quartz standard using the Oliver and Pharr methodology prior to testing.[3]

Thermogravimetric Analysis (TGA)

Samples were weighed on to a platinum sample pan. Samples were heated at 20 °C/min to 100 °C, followed by an isothermal period of 1 minute, and then heated at 10 °C/min to 600 °C under nitrogen.

Differential Scanning Calorimetry (DSC)

Samples (around 2–6 mg) were heated to 180 °C at 10 °C/min (150 °C for **P1**), followed by an isothermal period of 5 min. The samples were then cooled to −80 °C at 10 °C/min, followed by an isothermal period of 5 min. This process was repeated two times. Thermograms taken from the third heating/cooling cycle.

Nanoindentation

Samples for nanoindentation experiments were prepared by drop-casting a solution of 30 mg of polymer in 0.1 ml DCM onto a 1 cm square glass plate. The samples were left at room temperature for 4 h then annealed at 80 °C under vacuum for 4 h, at room temperature under vacuum for 16 h,

and then affixed onto steel pucks with epoxy. 5 x 5 Arrays of indentations with 20 µm spacing between neighboring indents were performed. All indents were load-controlled with a maximum applied load of 6000 µN, a loading and holding time of 10 s, and an unloading time of 2 s. The hardness (H) and reduced modulus (E_r) were then calculated from the unloading segments of the load-displacement curves using the standard Oliver and Pharr analysis.[3]

Monomer Synthesis

Monomer **1a**

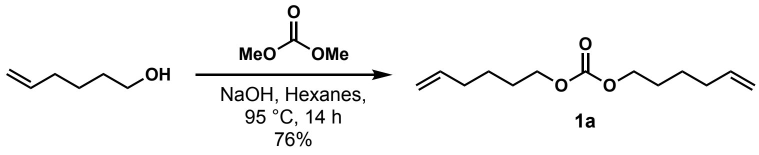

Synthesis adapted from a literature procedure.[1] Dimethyl carbonate (1.07 g, 1.0 mL, 11.9 mmol, 1 equiv), hexanes (12 mL), 5-hexen-1-ol (2.38 g, 2.85 mL, 23.8 mmol, 2 equiv) and NaOH (48 mg, 1.1 mmol, 0.1 equiv) were combined in a round bottom flask under air. The round bottom flask was fitted with a Dean-Stark apparatus. Water was added to fill up half of the volume of the Dean-Stark, followed by hexanes for the remainder of the volume. The Dean Stark was then fitted with a waterless condenser. The round bottom flask was lowered into an oil bath preheated to 95 °C. After stirring for 1 h, an additional batch of 5-hexen-1-ol (2.38 g, 2.85 mL, 23.8 mmol, 2 equiv) was added after cooling the reaction to room temperature. The round bottom flask was again lowered into the oil bath and allowed to stir overnight (14 h). The reaction was allowed to cool to room temperature. The reaction mixture was transferred to a separatory funnel where it was washed with water (3 x 10 mL). The organic portion was dried over $MgSO_4$, filtered, and the solvent was removed *in vacuo*. The resulting oil was purified through column chromatography (SiO_2; hexanes to 6:4 hexanes:DCM). The product was visualized with a $KMnO_4$ stain. Removal of solvent and subsequent drying on the high vacuum line provided the product as a clear oil. Yield: 2.04 g, 76%.

The spectroscopic data for this compound were identical to those reported in the literature.[4]

^{1}H NMR (400 MHz, $CDCl_3$) δ 5.86–5.72 (m, 2 H), 5.07–4.91 (m, 4 H), 4.13 (t, *J* = 6.7 Hz, 4 H), 2.09 (q, *J* = 7.1 Hz, 4 H), 1.69 (p, *J* = 6.7 Hz, 7.2 Hz, 4 H), 1.48 (p, *J* = 7.7 Hz, 7.5 Hz, 4 H) ppm. HRMS-APCI: calc'd. for $C_{13}H_{23}O_3$ $[M+H]^+$ 227.1639, found 227.1642.

Monomer **2a**

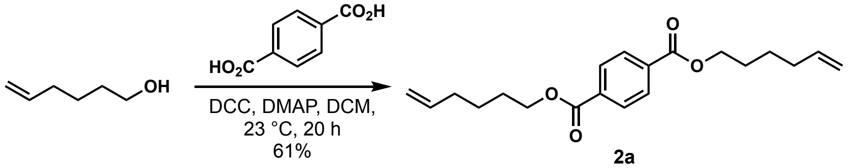

2a

Synthesis adapted from a literature procedure.[1] Terephthalic acid (829 mg, 4.97 mmol, 1 equiv), DCC (2.06 g, 9.98 mmol, 2 equiv), DMAP (122 mg, 1.0 mmol, 0.2 equiv), and DCM (40 mL) were combined in a round bottom flask equipped with a stir bar under air. 5-hexen-1-ol (1.0 g, 1.2 mL, 9.98 mmol, 2 equiv) was added dropwise to the stirring solution and upon complete addition, the reaction was stirred for 20 h at room temperature. Afterwards, the resulting white suspension was filtered through a pad of celite. The solvent was removed *in vacuo,* and the resulting oil was purified through column chromatography (SiO_2, 1:1 hexanes:DCM to 100% DCM). The pure product was obtained as a clear oil. Yield: 1.01 g, 61%

The spectroscopic data for this compound were identical to those reported in the literature.[5]

^{1}H NMR (400 MHz, $CDCl_3$) δ 8.10 (s, 4 h), 5.89–5.76 (m, 2 H), 5.08–4.94 (m, 4 H), 4.35 (t, J = 6.7 Hz, 4 H), 2.18–2.10 (q, J = 7.1 Hz, 4 H), 1.81 (p, J = 6.7 Hz, 7.0 Hz, 4 H), 1.56 (p, J = 7.7 Hz, 7.6 Hz, 4 H) ppm.

HRMS-APCI: calc'd. for $C_{20}H_{26}O_4$ $[M+H]^+$ 331.1904, found 331.1897.

Monomer **3a**

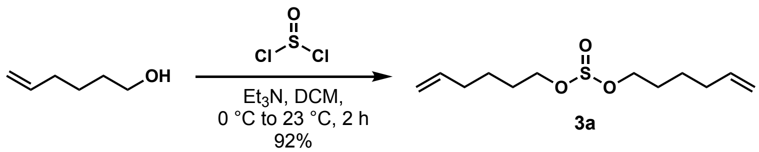

3a

Synthesis adapted from a literature procedure.[1] 5-hexen-1-ol (1.0 g, 1.2 mL, 10 mmol, 2 equiv), Et_3N (1.0 g, 1.4 mL, 10 mmol, 2 equiv), and DCM (17 mL) were combined in a flame-dried round-bottom flask equipped with a stir bar under N_2. The flask was cooled in an ice/water bath and thionyl chloride (595 mg, 0.36 mL, 5 mmol, 1 equiv) was added dropwise. After complete addition, the flask was allowed to warm to room temperature and was monitored by TLC until completion (2 h). The reaction was then quenched with water (20 mL). The organic layer was extracted with additional DCM (20 mL) and the organic layer was then washed with brine (20 mL). The organic layer was then separated and dried over sodium sulfate. The organic solvent was then removed *in*

vacuo and the oil was purified through column chromatography (SiO_2, 1:1 hexanes:DCM) to give the product as a clear oil (1.1 g, 92%).

^{1}H NMR (500 MHz, $CDCl_3$) δ 5.85–5.72 (m, 2 H), 5.06–4.93 (m, 4 H), 4.08–3.87 (m, 4 H), 2.13–2.04 (q, J = 7.0 Hz, 4 H), 1.74–1.65 (m, 4 H), 1.54–1.44 (m, 4 H) ppm.
^{13}C NMR (125 MHz, $CDCl_3$) δ 138.2, 115.0, 62.2, 33.2, 29.0, 25.1 ppm.
HRMS-APCI: calc'd. for $C_{12}H_{22}O_3S$ $[M+H]^+$ 247.1362, found 247.1359.

Compound **S1**

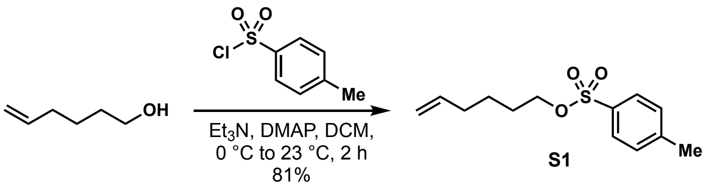

Synthesis adapted from a literature procedure.[1] Tosyl chloride (2.1 g, 11 mmol, 1.1 equiv) freshly recrystallized from benzene and DMAP (122 mg, 1 mmol, 0.1 equiv) were weighed into a flame-dried round-bottom flask equipped with a stir bar and the flask was subsequently subjected to vacuum on a Schlenk line followed by an N_2 refill. This process was repeated twice and then dry DCM (25 mL) was added. The solution was cooled to 0 °C in an ice/water bath. Then, 5-hexen-1-ol (1.0 g, 1.2 mL, 10 mmol, 1 equiv) and anhydrous Et_3N (2.3 g, 3.2 mL, 23 mmol, 2.3 equiv) were each added dropwise. After the addition, the flask was allowed to warm to room temperature and the reaction was monitored by TLC until complete consumption of the alcohol (2 h). The reaction was then washed with water (3 x 10 mL) followed by brine (10 mL). The organic layer was dried over $MgSO_4$. After filtering out the $MgSO_4$, the solvent was removed *in vacuo*. The resulting yellow residue was purified through column chromatography (SiO_2, 2:1 hexanes:DCM) to give the product as a clear oil. Yield 2.06 g, 81%

The spectroscopic data for this compound were identical to those reported in the literature.[6]
^{1}H NMR (400 MHz, $CDCl_3$) δ 7.81–7.76 (m, 2 H), 7.37–7.32 (m, 2 H), 5.78–5.65 (m, 1 H), 4.99–4.91 (m, 2 H), 4.03 (t, J = 6.4 Hz, 2 H), 2.45 (s, 3 H), 2.04–1.95 (q, J = 7.0 Hz, 2 H), 1.69–1.60 (m, 2 H), 1.45–1.36 (m, 2 H) ppm.

Monomer **4a**

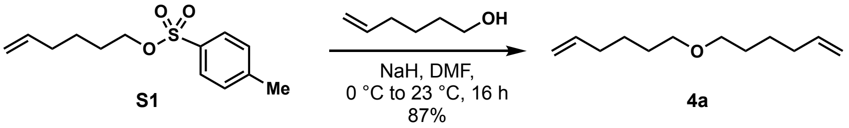

Synthesis adapted from a literature procedure.[1] Compound **S1** (2.06 g, 8.1 mmol, 1 equiv) was weighed into a flame-dried round-bottom flask equipped with a stir bar. The flask was then subjected to three vacuum/N_2 cycles. Dry DMF (27 mL) was added to the flask followed by NaH (60% dispersion in mineral oil) (388 mg, 9.7 mmol, 1.2 equiv). The flask was cooled to 0 °C in an ice/water bath. 5-hexen-1-ol (1.0 g, 1.2 mL, 10 mmol, 1.2 equiv) was added slowly to the reaction over 5 min. After addition of 5-hexen-1-ol, the reaction was allowed to warm to room temperature and was stirred for 16 h. Ether (25 mL) was then added to the reaction and the dilute solution was washed with saturated *aq.* NH_4Cl (3 x 10 mL). The organic layer was then dried over $MgSO_4$. Following the removal of $MgSO_4$, the solvents were removed *in vacuo* and the yellow-orange oil was purified through column chromatography (SiO_2, hexanes) to give the product as a clear oil. Yield: 1.3 g, 87%

The spectroscopic data for this compound were identical to those reported in the literature.[7]
^{1}H NMR (400 MHz, $CDCl_3$) δ 5.88–5.74 (m, 2 H), 5.05–4.91 (m, 4 H), 3.40 (t, J = 6.6 Hz, 4 H), 2.11–2.03 (m, 4 H), 1.63–1.54 (m, 4 H), 1.50–1.40 (m, 4 H) ppm.
HRMS-APCI: calc'd. for $C_{12}H_{22}O$ $[M+H]^+$ 183.1741, found 183.1743.

General Siloxane Monomer Procedure

To a flame dried round-bottom flask, imidazole (2.1 equiv) and dry THF (1 M with respect to alcohol) were added. The flask was cooled to 0 °C with an ice/water bath and dichlorodimenthylsilane (1 equiv) was added dropwise. After stirring at room temperature for 1 h, the mixture was cooled to 0 °C and treated with of the unsaturated alcohol (1.0 mL, 2.1 equiv). The cloudy suspension was stirred at room temperature for 16 h and then diluted with 50 mL of hexanes, washed with saturated *aq.* NH_4Cl (20 mL), water (20 mL) and brine (20 mL). The organic layer was dried over sodium sulfate and the solvent was removed *in vacuo* to provide the monomer without further purification.

Monomer **6a**

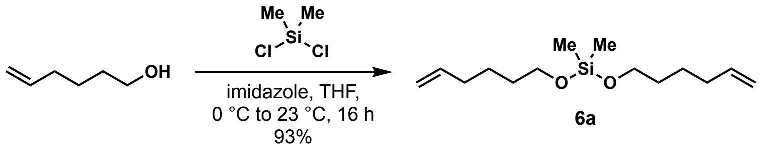

Monomer **6a** was synthesized following the **General Siloxane Monomer Procedure** using 5-hexen-1-ol (1.0 mL, 8.33 mmol), imidazole (8.33 mmol), dichlorodimethylsilane (3.97 mmol, 0.48 mL), and THF (8.0 mL). Product isolated as a colorless oil (0.95 g, 3.70 mmol, 93% yield).

^{1}H NMR (400 MHz, $CDCl_3$) δ 5.87–5.73 (m, 2 H), 5.05–4.88 (m, 4 H), 3.67 (t, J = 6.6 Hz, 4 H), 2.12–2.01 (m, 4 H), 1.63–1.50 (m, 4 H), 1.49–1.36 (m, 4 H), 0.11 (s, 6 H) ppm.
^{13}C NMR (101 MHz, $CDCl_3$) δ 138.9, 114.6, 62.4, 33.6, 32.2, 25.3, −3.1 ppm.
HRMS-APCI: calc'd. for $C_{14}H_{28}O_2Si$ $[M+H]^+$ 257.1931, found 257.1930.

Monomer **6b**

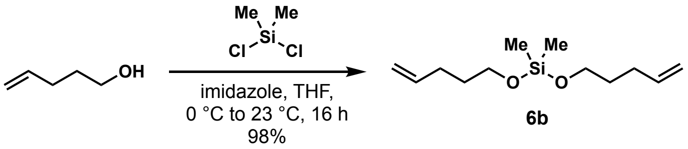

Monomer **6b** was synthesized following the **General Siloxane Monomer Procedure** using 4-penten-1-ol (1.0 mL, 9.68 mmol), imidazole (9.68 mmol), dichlorodimethylsilane (4.61 mmol, 0.56 mL), and THF (9.2 mL). Product isolated as a colorless oil (1.03 g, 4.50 mmol, 98% yield).
^{1}H NMR (400 MHz, $CDCl_3$) δ 5.88–5.74 (m, 2 H), 5.07–4.91 (m, 4 H), 3.68 (t, J = 6.6 Hz, 4 H), 2.15–2.06 (m, 4 H), 1.69–1.60 (m, 4 H), 0.11 (s, 6 H) ppm.
^{13}C NMR (101 MHz, $CDCl_3$) δ 138.4, 114.8, 62.0, 31.8, 30.1, −3.1 ppm.
HRMS-APCI: calc'd. for $C_{12}H_{24}O_2Si$ $[M+H]^+$ 229.1618, found 229.1618.

General Halogen Monomer Procedure

Triphenylphosphine (1.1 equiv) was added to a reaction vial and dissolved in DCM (2.0 M with respect to alcohol). Then, the alcohol (1 equiv) was added to the vial with stirring and the mixture was cooled in an ice water bath to 0 °C. Next, either NCS (1.1 equiv, for **7a** and **7b**) or NBS (1.1

equiv, for **8a** and **8b**) was added slowly in portions to the reaction mixture. The reaction was removed from the ice water bath and left to stir at room temperature for 16 h. After removing the solvent *in vacuo*, the reaction was purified using column chromatography (SiO_2, pentane) to give the product.

Monomer **7a**

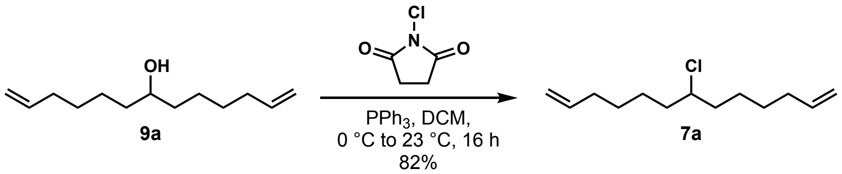

Monomer **7a** was synthesized following the **General Halogenation Monomer Procedure** using triphenyl phosphine (588 mg, 2.2 mmol, 1.1 equiv), DCM (4.1 mL), **9a** (400 mg, 2.0 mmol, 1 equiv), and NCS (299 mg, 2.2 mmol, 1.1 equiv). Product isolated as clear oil (359 mg, 82%).

^{1}H NMR (400 MHz, $CDCl_3$) δ 5.89–5.77 (m, 2 H), 5.07–4.94 (m, 4 H), 3.95–3.87 (m, 1 H), 2.15–2.04 (m, 4 H), 1.80–1.65 (m, 4 H), 1.65–1.35 (m, 8 H) ppm.
^{13}C NMR (101 MHz, $CDCl_3$) δ 138.9, 114.7, 64.2, 38.5, 33.8, 28.6, 26.1 ppm.
HRMS-APCI: calc'd. for $C_{13}H_{23}Cl$ $[M–H]^+$ 213.1405, found 213.1415.

Monomer **7b**

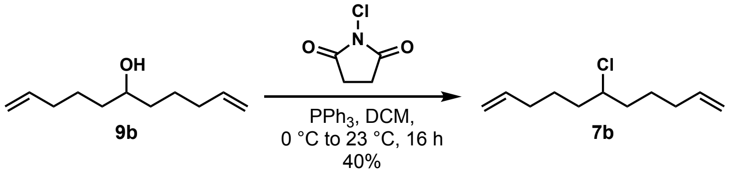

Monomer **7b** was synthesized following the **General Halogenation Monomer Procedure** using triphenyl phosphine (857 mg, 3.3 mmol, 1.1 equiv), DCM (5.9 mL), **9b** (500 mg, 3.0 mmol, 1 equiv), and NCS (436 mg, 3.3 mmol, 1.1 equiv). Product isolated as clear oil (222 mg, 40%).

The spectroscopic data for this compound were identical to those previously reported in the literature.[8]
^{1}H NMR (400 MHz, $CDCl_3$) δ 5.85–5.74 (m, 2 H), 5.06–4.94 (m, 4 H), 3.94–3.86 (m, 1 H), 2.15–2.00 (m, 4 H), 1.80–1.44 (m, 8 H) ppm.
HRMS-APCI: calc'd. for $C_{11}H_{19}Cl$ $[M–H]^+$ 185.1092, found 185.1100.

Monomer **8a**

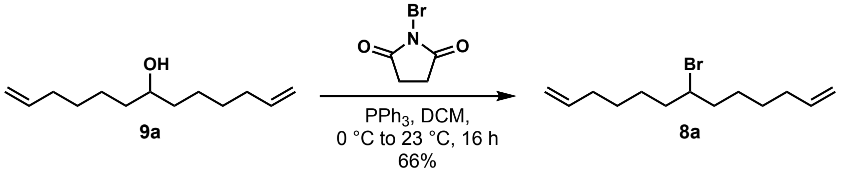

Monomer **8a** was synthesized following the **General Halogenation Monomer Procedure** using triphenyl phosphine (294 mg, 1.1 mmol, 1.1 equiv), DCM (2.0 mL), **9a** (200 mg, 2.0 mmol, 1 equiv), and NBS (199 mg, 1.1 mmol, 1.1 equiv). Product isolated as clear oil (174 mg, 66%).

^{1}H NMR (400 MHz, $CDCl_3$) δ 5.86–5.75 (m, 2 H), 5.00–4.92 (m, 4H), 4.06–3.98 (m, 1 H), 2.11–2.01 (m, 4H), 1.89–1.74 (m, 4H), 1.62–1.32 (m, 8H) ppm.
^{13}C NMR (101 MHz, $CDCl_3$) δ 138.8, 114.7, 58.7, 39.1, 33.7, 28.5, 27.2 ppm.
HRMS-APCI: calc'd. for $C_{13}H_{23}Br$ $[M+H]^+$ 259.1058, found 259.1053.

Monomer **8b**

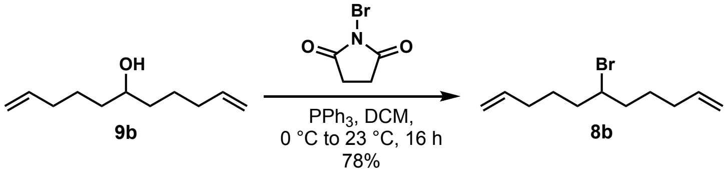

Monomer **8b** was synthesized following the **General Halogenation Monomer Procedure** using triphenyl phosphine (514 mg, 2.0 mmol, 1.1 equiv), DCM (3.6 mL), **9b** (300 mg, 1.8 mmol, 1 equiv), and NBS (349 mg, 2.0 mmol, 1.1 equiv). Product isolated as clear oil (320 mg, 78%).

The spectroscopic data for this compound were identical to those previously reported in the literature.[9]
^{1}H NMR (400 MHz, $CDCl_3$) δ 5.85–5.74 (m, 2 H), 5.06–4.94 (m, 4H), 4.07–3.99 (m, 1 H), 2.16–2.00 (m, 4H), 1.90–1.76 (m, 4H), 1.74–1.45 (m, 4H) ppm.
HRMS-APCI: calc'd. for $C_{13}H_{23}Br$ $[M+H]^+$ 231.0743, found 231.0758.

General Alcohol Monomer Synthesis

Synthesis adapted from literature procedure.[10] In a flame-dried 250 mL round bottom flask under N_2, a suspension of Mg turnings (2.5 equiv) and iodine (0.05 equiv) in dry THF (0.5 M with respect to the bromoalkene) was prepared. The bromoalkene (2.5 equiv) was then slowly added to the

suspension, which was stirred for 45 min at room temperature. The mixture was then cooled to 0 °C in an ice bath, and freshly distilled ethyl formate (1 equiv) was then slowly added to the mixture. After removing the ice bath, the reaction was left to stir at room temperature for 16 h. The reaction was quenched with saturated *aq.* NH_4Cl and extracted with diethyl ether (3 x 20 mL). The combined organics were then washed with water (40 mL), dried with Na_2SO_4, filtered, and the volatiles were removed *in vacuo*. The product was purified through column chromatography (SiO_2, hexanes to 19:1 hexanes:diethyl ether) to give the product as a clear oil.

Monomer **9a**

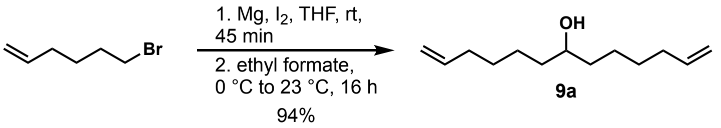

Synthesized following **General Alcohol Monomer Procedure** using Mg turnings (488 mg, 20.0 mmol, 2.5 equiv), iodine (102 mg, 0.4 mmol, 0.05 equiv), dry THF (40 mL), 6-bromo-1-hexene (2.6 mL, 20.0 mmol, 2.5 equiv), and ethyl formate (0.6 mL, 8.0 mmol, 1 equiv). Yield: 1.3 g, 80%.

^{1}H NMR (400 MHz, $CDCl_3$) δ 5.87–5.75 (m, 2 H), 5.04–4.91 (m, 4 H), 3.63–3.55 (m, 1 H), 2.11–2.02 (m, 4 H), 1.54–1.28 (m, 13 H) ppm.
^{13}C NMR (101 MHz, $CDCl_3$) δ 139.0, 114.5, 72.2, 37.4, 33.9, 29.1, 25.3 ppm.
HRMS-APCI: calc'd. for $C_{13}H_{24}O$ $[M+H]^+$ 197.1900, found 197.1896.

Monomer **9b**

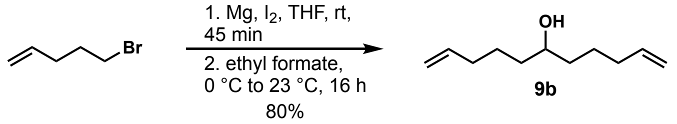

Synthesized following **General Alcohol Monomer Procedure** using Mg turnings (533 mg, 21.9 mmol, 2.5 equiv), iodine (111 mg, 0.4 mmol, 0.05 equiv), dry THF (43 mL), 5-bromo-1-pentene (2.6 mL, 21.9 mmol, 2.5 equiv), and ethyl formate (0.7 mL, 8.8 mmol, 1 equiv). Yield: 1.4 g, 94%.

The spectroscopic data for this compound were identical to those previously reported in the literature.[10]

^{1}H NMR (400 MHz, $CDCl_3$) δ 5.87–5.74 (m, 2H), 5.05–4.92 (m, 4H), 3.65–3.56 (m, 1H), 2.14–2.00 (m, 4H), 1.60–1.34 (m, 9 H) ppm.

HRMS-APCI: calc'd. for $C_{11}H_{20}O$ $[M+H]^+$ 169.1587, found 169.1583.

Polymerization Procedures

Optimization of the *cis*-Selective ADMET

In an N_2-filled glovebox, a solution of **1a** (0.20 mmol, 1 equiv) in TCB (2–5 M) was added to another vial containing catalyst (1–2 μmol, 0.005–0.01 equiv). The solution was then transferred to an oven-dried Schlenk flask through a red rubber septum secured with electrical tape. A Teflon stopper was then fitted into the sidearm. The flask was then removed from the glovebox and the flask was placed under vacuum using a Schlenk line. The flask was then either stirred at room temperature or lowered into a preheated oil bath for the entirety of the reaction time. After the polymerization was finished, the flask was placed under static vacuum and ethyl vinyl ether (~50 μL) was added at room temperature. After stirring for 5 min, the flask was opened to air and an aliquot was removed for NMR analysis. The polymer was dissolved in dichloromethane and precipitated by addition of cold methanol in a centrifuge tube. After centrifugation at 8500 RPM for 10 min, the supernatant was decanted. This process was repeated again and the polymer was dried under vacuum to afford a highly viscous brown oil.

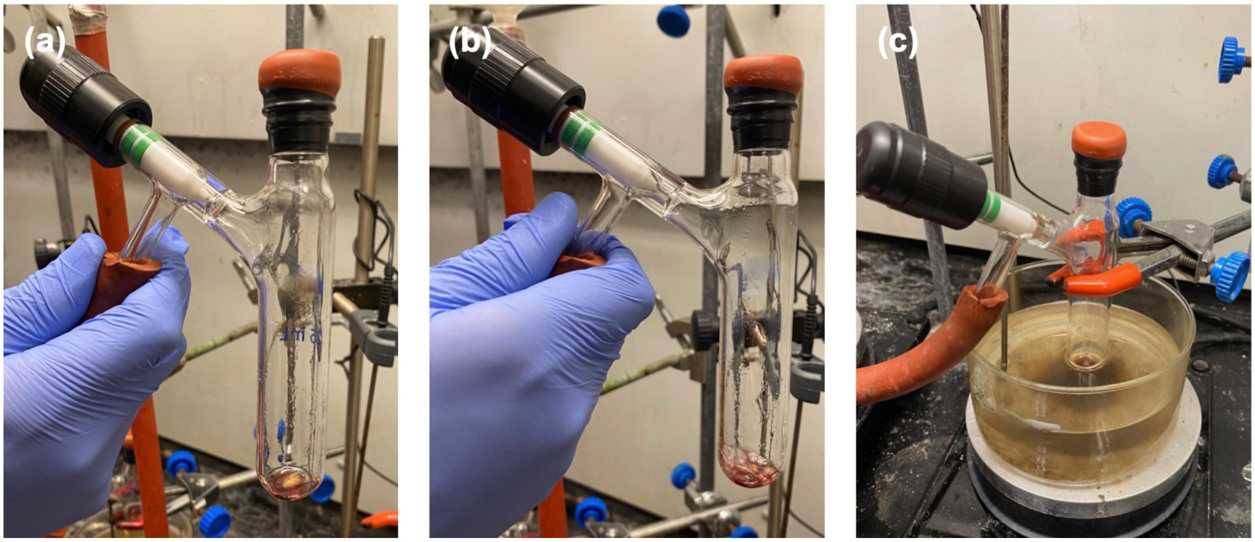

Figure S1. Typical ADMET reaction setup with **Ru-1**: (a) Schlenk flask charged with polymerization solution removed from the glovebox and attached to high vacuum line, (b) vacuum applied to the system, and (c) flask lowered into preheated oil bath.

Table S1. Polymerization Results for **P1a**

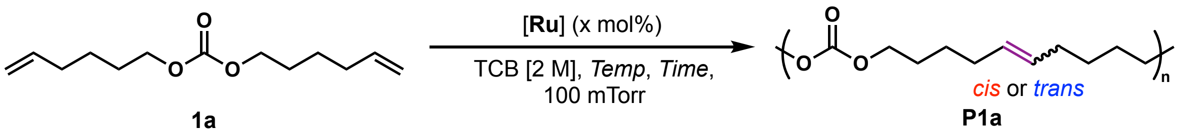

Entry	Catalyst (mol%)	Temp (°C)	t (h)	M_n (kg/mol)[a]	*Đ*	*cis* (%)[b]
**1**	**Ru-1** (1)	80	16	27.9	1.75	14
**2**	**Ru-3a** (1)	80	16	17.7	1.73	18
**3**	**Ru-3b** (1)	80	16	28.7	2.99	38
**4**	**Ru-3b** (1)	40	16	13.5	1.75	97
**5**	**Ru-3a** (1)	40	16	15.8	1.83	56
**6**	**Ru-3b** (1)	23	16	9.8	1.67	99
**7**	**Ru-3a** (1)	23	16	11.5	1.80	72
**8**	**Ru-3b** (0.5)	23	16	6.0	1.42	99
**9**	**Ru-3b** (1)	23	40	12.8	1.58	99
**10**	**Ru-3b** (1)	23	8	7.3	1.39	99
**11**	**Ru-3b** (1)	23	6	7.8	1.59	99
**12**	**Ru-3b** (1)	23	2	5.5	1.30	99
**13**[c]	**Ru-3b** (1)	23	16	9.9	1.47	99
**14**[c]	**Ru-3a** (1)	23	16	11.2	1.61	80
**15**[c]	**Ru-3c** (1)	23	16	6.8	1.52	89
**16**[d,e]	**Ru-3b** (1)	23	16	5.1	1.28	99
**17**[d,e]	**Ru-3b** (1)	80	16	14.1	1.97	58
**18**[c,d]	**Ru-4** (2)	23	4	–	–	–

[a]Determined through SEC in THF against polystyrene standards. [b]Calculated using ^{1}H NMR analysis [c]Polymerization carried out at a concentration of 5 M. [d]Reaction performed on 0.5 mmol scale with respect to the monomer. [e]Polymerization carried out in the bulk.

Comparison of Oxygen Sensitivity Between Ru-3b **and** Ru-4 **in ADMET**

In an N_2-filled glovebox, **Ru-3b** (3.4 mg, 0.05 mmol, 0.01 equiv) and **Ru-4** (2.1 mg, 0.0025 mmol, 0.005 equiv) were weighed into two separate, oven-dried vials equipped with stir bars and dissolved in TCB (0.1 mL). The vials were sealed with a screwcap fitted with a teflon septa. The vials were removed from the glovebox and the septa was pierced with an 18G needle. The vials were then stirred at room temperature (23 °C) for 20 min under air. Next, the catalyst solution was transferred to an oven-dried Schlenk flask under N_2 containing a stir bar and either monomer **1a** (113 mg, 0.5 mmol, 1 equiv) for **Ru-3b** or capped monomer **S2**[1] (113 mg, 0.5 mmol, 1 equiv) for **Ru-4**. The flask was then placed under vacuum (100 mTorr) and reactions were stirred at room temperature (23 °C) for either 4 h (**Ru-4**) or 16 h (**Ru-3b**). The flask was then placed under static vacuum and ethyl vinyl ether (~50 µL) was added at room temperature. After stirring for 5 min, the flask was opened to air and an aliquot was removed for NMR analysis. In the attempted polymerization of **S2** with **Ru-4**, ^{1}H NMR analysis revealed mostly recovery of the starting material with trace oligomer formation. In the case of the polymerization of **1a** with **Ru-3b**, the polymer was dissolved in dichloromethane and precipitated by addition of cold methanol in a centrifuge tube. After centrifugation at 8500 RPM for 10 min, the supernatant was decanted, and the polymer was dried under vacuum to afford a highly viscous brown oil.

Table S2. Effect of Oxygen on Stereoretentive (**Ru-4**) and *cis*-Selective ADMET (**Ru-3b**)

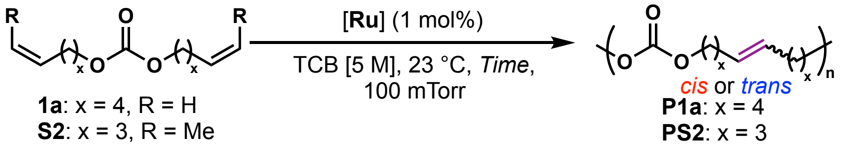

Entry	Monomer	Catalyst (mol%)	t (h)	M_n (kg/mol)	$Đ$	*cis* (%)
**1**	**1a**	**Ru-3b** (1)	16	8.9	1.51	99
**2**	**S2**	**Ru-4** (0.5)	4	–[a]	–	–

[a]No polymer isolated. Only trace oligomer formation observed in ^{1}H NMR

General *cis*-Selective ADMET Polymerization Procedure Using Vacuum (100 mTorr)

In an N_2 filled glovebox, a solution of monomer (0.50 mmol, 1 equiv) in 0.1 mL of TCB was added to another vial containing **Ru-3b** (3.4 mg, 5.0 µmol, 0.01 equiv). The solution was then transferred

to an oven-dried Schlenk flask through a red rubber septum secured with electrical tape. A Teflon stopper was then fitted into the sidearm. The flask was then removed from the glovebox and the flask was placed under vacuum using a Schlenk line. The flask was then stirred at room temperature for 16 h. After the polymerization was finished, the flask was placed under static vacuum and ethyl vinyl ether (~50 μL) was added. After stirring for 5 min, the flask was opened to air and an aliquot was removed for NMR analysis. The polymerization was then purified through dissolving the polymer in DCM and precipitating in cold methanol in a centrifuge tube. After centrifugation at 8500 RPM for 10 min, the methanol was decanted. The polymer was then dissolved in DCM and reprecipitated in MeOH before undergoing another centrifugation cycle. After decanting the MeOH, the polymer was dried for 24 h under vacuum.

Table S3. Polymerization Results for **P1a**

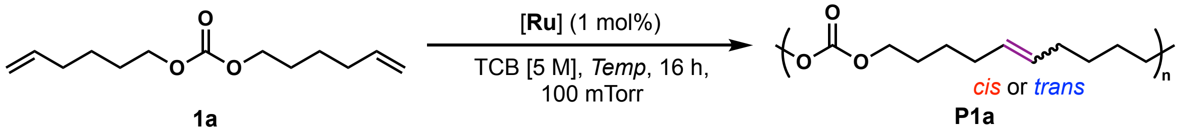

Entry	Catalyst	Temp (°C)	M_n (kg/mol)	$Đ$	*cis* (%)
**1**	**Ru-1**	80	20.9	1.81	15
**2**	**Ru-3b**	23	12.0	1.60	99

The spectroscopic data for the *cis*-rich **P1a** were identical to those previously reported in the literature.[1]

cis-rich **P1a**: ^{1}H NMR (400 MHz, $CDCl_3$) δ 5.39–5.28 (m, 2 H), 4.11 (t, J = 6.7 Hz, 4 H), 2.11–1.96 (m, 4 H), 1.72–1.60 (m, 4 H), 1.47–1.36 (m, 4 H) ppm.

The spectroscopic data for the *trans*-rich **P1a** were identical to those previously reported in the literature.[4]

trans-rich **P1a**: ^{1}H NMR (400 MHz, $CDCl_3$) δ 5.45–5.32 (m, 2 H), 4.12 (t, J = 6.7 Hz, 4 H), 2.11–1.95 (m, 4 H), 1.77–1.59 (m, 4 H), 1.48–1.34 (m, 4 H) ppm

Table S4. Polymerization Results for **P1b**

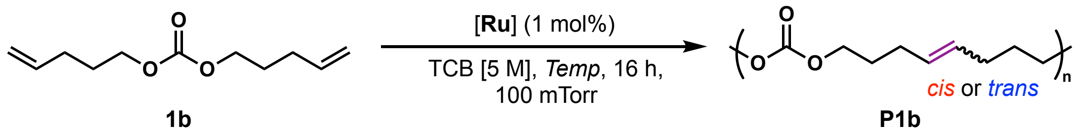

Entry	catalyst	Temp (°C)	M_n (kg/mol)	*Đ*	*cis* (%)
**1**	**Ru-1**	80	15.7	1.80	17
**2**	**Ru-3b**	23	9.1	1.38	99

The spectroscopic data for the *cis*-rich **P1b** were identical to those previously reported in the literature.[1]

cis-rich **P1b**: ^{1}H NMR (400 MHz, $CDCl_3$) δ 5.44–5.34 (m, 2 H), 4.11 (t, *J* = 6.6 Hz, 4 H), 2.18–2.04 (m, 4 H), 1.78–1.67 (m, 4 H) ppm.

The spectroscopic data for the *trans*-rich **P1b** were identical to those previously reported in the literature.[4]

trans-rich **P1b**: ^{1}H NMR (400 MHz, $CDCl_3$) δ 5.47–5.36 (m, 2 H), 4.12 (t, *J* = 6.6 Hz, 4 H), 2.18–1.99 (m, 4 H), 1.79–1.67 (m, 4 H) ppm.

Table S5. Polymerization Results for **P2a**

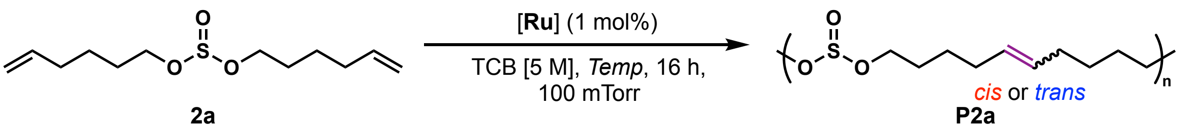

Entry	catalyst	Temp (°C)	M_n (kg/mol)	*Đ*	*cis* (%)
**1**	**Ru-1**	80	18.8	1.77	23
**2**	**Ru-3b**	23	8.4	1.42	99

The spectroscopic data for the *cis*-rich **P2a** were identical to those previously reported in the literature.[1]

cis-rich **P2a**: ^{1}H NMR (400 MHz, $CDCl_3$) δ 5.42–5.31 (m, 2 H), 4.09–3.87 (m, 4 H), 2.15–1.99 (m, 4 H), 1.74–1.62 (m, 4 H), 1.50–1.38 (m, 4 H) ppm.

trans-rich **P2a**: ^{1}H NMR (400 MHz, $CDCl_3$) δ 5.44–5.30 (m, 2 H), 4.07–3.84 (m, 4 H), 2.09–1.96 (m, 4 H), 1.70–1.60 (m, 4 H), 1.48–1.37 (m, 4 H) ppm.
^{13}C NMR (101 MHz, $CDCl_3$) δ 130.3, 129.7, 62.2, 32.0, 29.2, 29.0, 26.8, 25.9, 25.7 ppm

Table S6. Polymerization Results for **P2b**

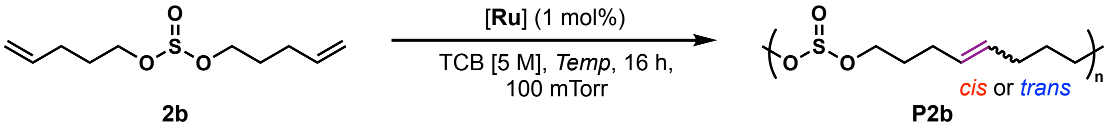

Entry	catalyst	Temp (°C)	M_n (kg/mol)	*Đ*	*cis* (%)
**1**	**Ru-1**	80	9.9	1.67	23
**2**	**Ru-3b**	23	9.5	1.56	99

The spectroscopic data for the *cis*-rich **P2b** were identical to those previously reported in the literature.[1]
cis-rich **P2b**: ^{1}H NMR (400 MHz, $CDCl_3$) δ 5.44–5.34 (m, 2 H), 4.10–3.86 (m, 4 H), 2.20–2.08 (m, 4 H), 1.79–1.69 (m, 4 H) ppm.

The spectroscopic data for the *trans*-rich **P2b** were identical to those previously reported in the literature.[11]
trans-rich **P2b**: ^{1}H NMR (400 MHz, CDCl3) δ 5.46–5.37 (m, 2 H), 4.08–3.86 (m, 4 H), 2.19–2.05 (m, 4 H), 1.78– 1.69 (m, 4 H) ppm.

Table S7. Polymerization Results for **P3a**

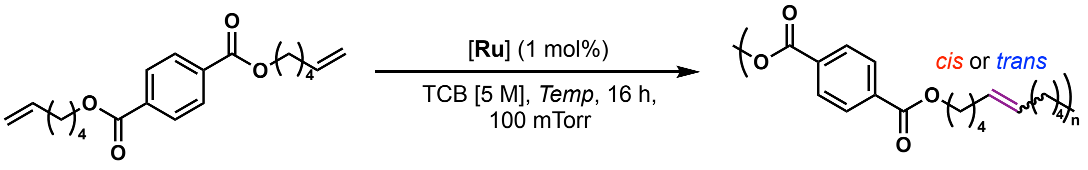

Entry	catalyst	Temp (°C)	M_n (kg/mol)	$Đ$	*cis* (%)
**1**	**Ru-3b**	23	8.3	1.52	91
**2**	**Ru-1**	80	7.3	3.28	21

The spectroscopic data for the *cis*-rich **P3a** were identical to those previously reported in the literature.[1]

cis-rich **P3a**: ^{1}H NMR (400 MHz, CDCl3) δ 8.08 (s, 4), 5.46–5.34 (m, 2 H), 4.34 (t, J = 6.6 Hz, 4 H), 2.17–2.05 (m, 4 H), 1.84–1.74 (m, 4 H), 1.55–1.46 (m, 4 H) ppm.

The spectroscopic data for the *trans*-rich **P3a** were identical to those previously reported in the literature.[5]

trans-rich **P3a**: ^{1}H NMR (400 MHz, CDCl3) δ 8.08 (s, 4 H), 5.49–5.36 (m, 2 H), 4.33 (t, J = 6.6 Hz, 4 H), 2.17– 2.03 (m, 4 H), 1.84–1.73 (m, 4 H), 1.56–1.46 (m, 4 H) ppm.

Table S8. Polymerization Results for **P3b**

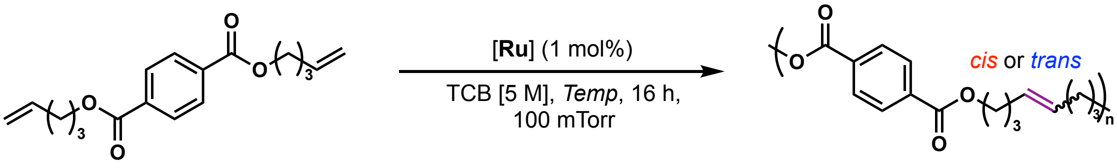

Entry	catalyst	Temp (°C)	M_n (kg/mol)	$Đ$	*cis* (%)
**1**	**Ru-1**	80	27.9	1.75	14
**2**	**Ru-3b**	23	5.0	1.69	99

The spectroscopic data for the *cis*-rich **P3b** were identical to those previously reported in the literature.[1]

cis-rich **P3b**: ^{1}H NMR (400 MHz, $CDCl_3$) δ 8.08–8.04 (s, 4 H), 5.52–5.41 (m, 2 H), 4.31 (t, *J* = 6.7 Hz, 4 H), 2.27–2.14 (m, 4 H), 1.89–1.78 (m, 4 H) ppm.

The spectroscopic data for the *trans*-rich **P3b** were identical to those previously reported in the literature.[5]

trans-rich **P3b**: ^{1}H NMR (400 MHz, CDCl3) δ 8.11–8.05 (m, 4 H), 5.53–5.44 (m, 2 H), 4.38–4.28 (t, *J* = 6.7 Hz, 4 H), 2.26–2.12 (m, 4 H), 1.90–1.79 (m, 4 H) ppm.

Table S9. Polymerization Results for **P4a**

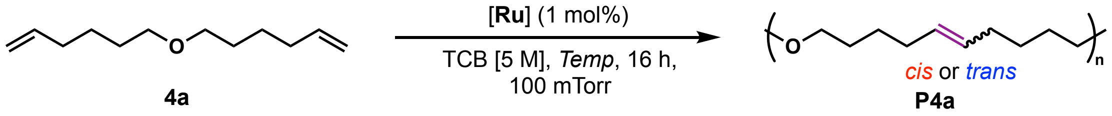

Entry	catalyst	T (°C)	M_n (kg/mol)	*Đ*	*cis* (%)
**1**	**Ru-1**	80	26.5	1.55	18
**2**	**Ru-3b**	23	10.0	1.49	99

The spectroscopic data for the *cis*-rich **P4a** were identical to those previously reported in the literature.[1]

cis-rich **P4a**: ^{1}H NMR (400 MHz, $CDCl_3$) δ 5.42–5.29 (m, 2 H), 3.39 (t, *J* = 6.6 Hz, 4 H), 2.10–1.95 (m, 4 H), 1.61–1.50 (m, 4 H), 1.44–1.34 (m, 4 H) ppm.

The spectroscopic data for the *trans*-rich **P4a** were identical to those previously reported in the literature.[7]

trans-rich **P4a**: ^{1}H NMR (400 MHz, $CDCl_3$) δ 5.45–5.32 (m, 2 H), 3.38 (t, *J* = 6.6 Hz, 4 H), 2.09–1.94 (m, 4 H), 1.63–1.48 (m, 4 H), 1.45–1.33 (m, 4 H) ppm.

Table S10. Polymerization Results for **P4b**

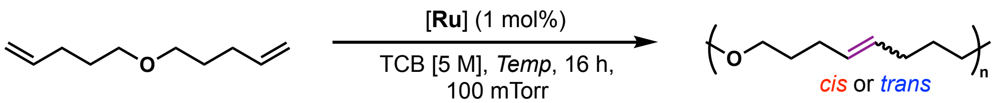

4b P4b

Entry	catalyst	T (°C)	M_n (kg/mol)	$Đ$	*cis* (%)
**1**[a]	**Ru-1**	50[b]	22.4	1.61	23
**2**[a]	**Ru-3b**	23	10.6	1.35	99

[a]Reaction performed at a scale of 1 mmol with respect to **4b**. [b]Reaction temperature lowered due to the volatility of **4b** under vacuum at high temperature.

The spectroscopic data for the *cis*-rich **P4b** were identical to those previously reported in the literature.[1]

cis-rich **P4b**: ^{1}H NMR (400 MHz, $CDCl_3$) δ 5.42–5.29 (m, 2 H), 3.39 (t, J = 6.6 Hz, 4 H), 2.10–1.95 (m, 4 H), 1.61–1.50 (m, 4 H), 1.44–1.34 (m, 4 H) ppm.

The spectroscopic data for the *trans*-rich **P4b** were identical to those previously reported in the literature.[7]

trans-rich **P4b**: ^{1}H NMR (400 MHz, $CDCl_3$) δ 5.45–5.35 (m, 2 H), 3.39 (t, J = 6.6 Hz, 4 H), 2.13–1.97 (m, 4 H), 1.67–1.58 (m, 4 H) ppm.

Table S11. Polymerization Results for **P6a**

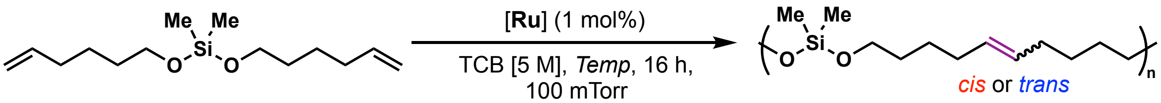

6a P6a

Entry	catalyst	T (°C)	M_n (kg/mol)	$Đ$	*cis* (%)
**1**	**Ru-1**	80	19.1	2.02	13
**2**	**Ru-3b**	23	10.3	1.45	99

cis-rich **P6a**: ^{1}H NMR (400 MHz, $CDCl_3$) δ 5.42–5.30 (m, 2 H), 3.67 (t, *J* = 6.6 Hz, 4 H), 2.10–1.95 (m, 4 H), 1.61–1.50 (m, 4 H), 1.44–1.33 (m, 4 H), 0.11 (s, 6 H) ppm.

^{13}C NMR (101 MHz, $CDCl_3$) δ 129.9, 62.6, 32.4, 27.1, 26.1, –3.0 ppm.

Basic hydrolysis of *cis*-rich **P6a** delivered the corresponding *cis*-dec-5-ene-1,10-diol.

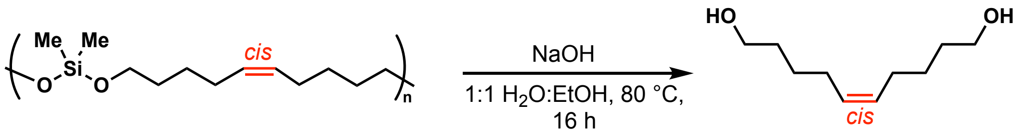

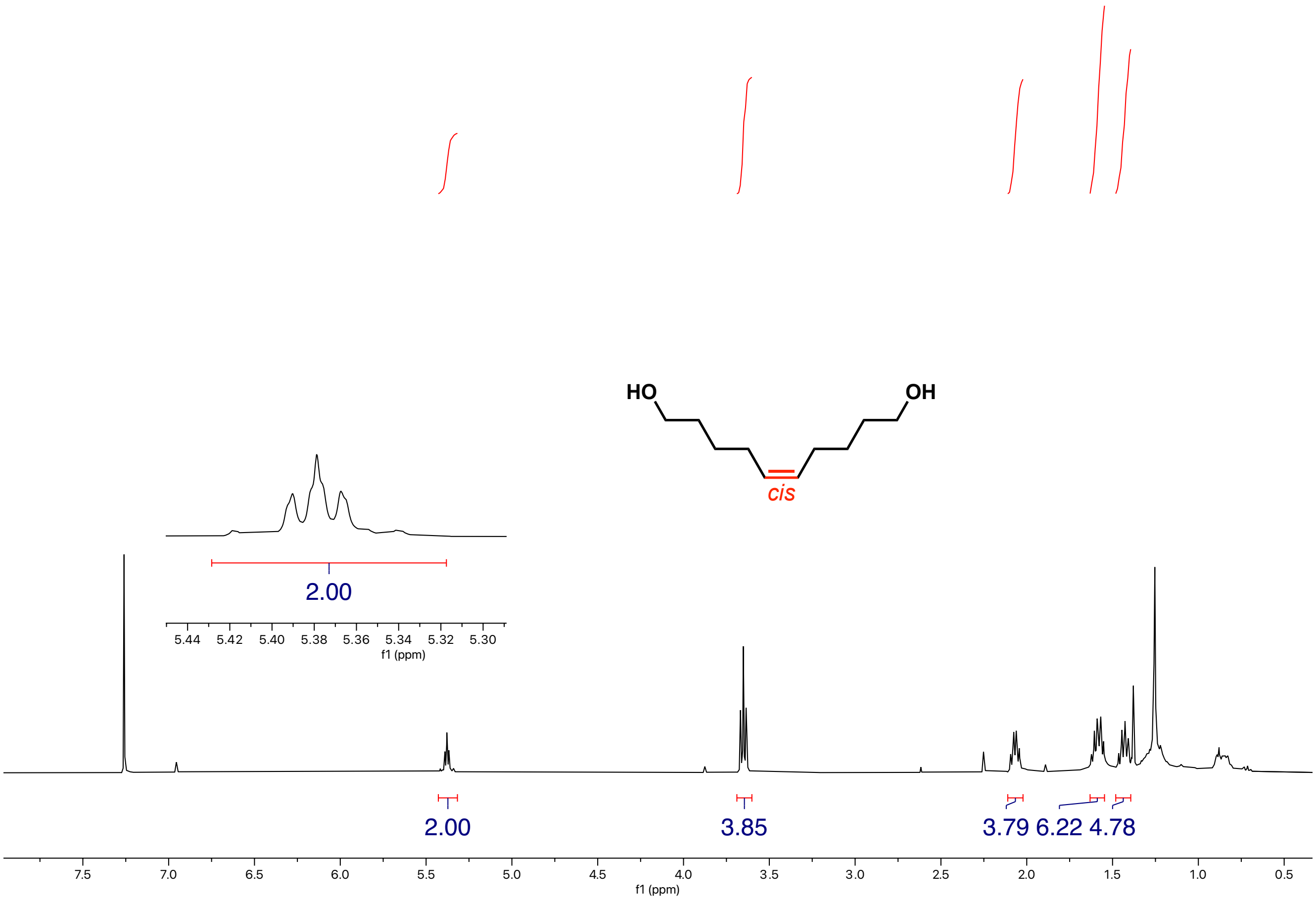

Figure S2. ^{1}H NMR (400 MHz) of dec-5-ene-1,10-diol obtained from hydrolysis of *cis* **P6a**.

trans-rich **P6a**: ^{1}H NMR (400 MHz, $CDCl_3$) δ 5.45–5.31 (m, 2 H), 3.66 (t, *J* = 6.6 Hz 4 H), 2.10–1.94 (m, 4 H), 1.64–1.49 (m, 4 H), 1.45–1.31 (m, 4 H), 0.11 (s, 6 H) ppm.

^{13}C NMR (101 MHz, $CDCl_3$) δ 130.3, 62.4, 32.3, 32.1, 25.8, –3.0 ppm

Table S12. Polymerization Results for **P6b**

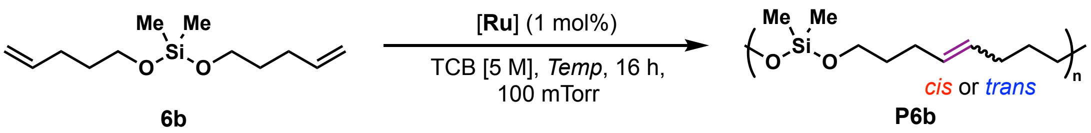

Entry	catalyst	T (°C)	M_n (kg/mol)	*Đ*	*cis* (%)
**1**	**Ru-1**	80	28.9	1.40	20
**2**	**Ru-3b**	23	17.6	1.29	99

cis-rich **P6b**: ^{1}H NMR (400 MHz, $CDCl_3$) δ 5.45–5.32 (m, 2 H), 3.67 (t, *J* = 6.7 Hz, 4 H), 2.14–2.00 (m, 4 H), 1.67–1.53 (m, 4 H), 0.11 (s, 6 H) ppm.

^{13}C NMR (101 MHz, $CDCl_3$) δ 129.7, 62.1, 32.7, 27.2, 23.6, –3.0 ppm.

Basic hydrolysis of *cis*-rich **P6b** delivered the corresponding *cis*-oct-4-ene-1,8-diol.

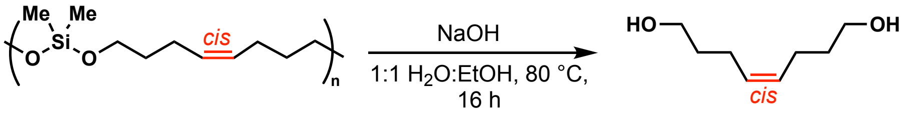

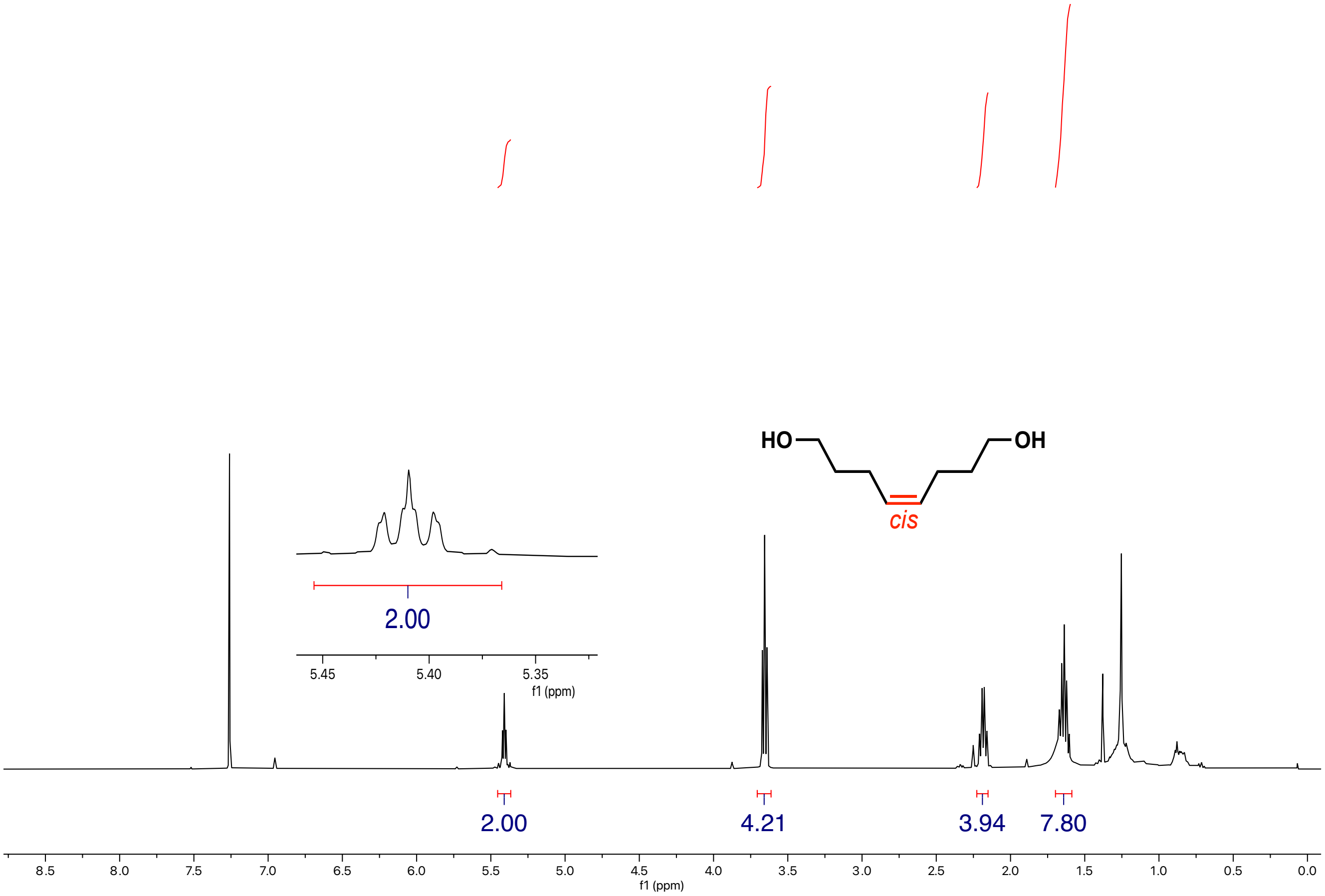

Figure S3 ^{1}H NMR (400 MHz) of oct-4-ene-1,8-diol obtained from hydrolysis of *cis* **P6b**.

trans-rich **P6b**: ^{1}H NMR (400 MHz, $CDCl_3$) δ 5.52–5.32 (m, 2 H), 3.66 (t, *J* = 6.7 Hz, 4 H), 2.34–1.95 (m, 4 H), 1.67–1.52 (m, 4 H), 0.11 (s, 6 H) ppm.

^{13}C NMR (101 MHz, $CDCl_3$) δ 130.2, 62.1, 32.7, 32.6, 29.0, –3.0 ppm

Table S13. Polymerization Results for **P7a**

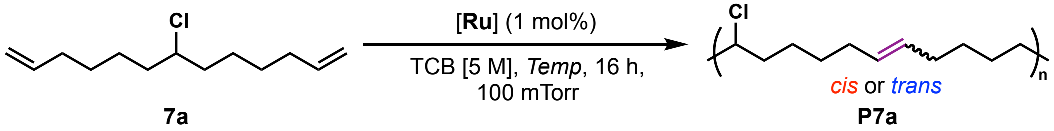

Entry	catalyst	T (°C)	M_n (kg/mol)	*Đ*	*cis* (%)
**1**	**Ru-1**	40[a]	16.2	1.87	19
**2**	**Ru-3b**	23	7.3	1.64	92

[a]Reaction temperature lowered due to the volatility of **7a** under vacuum at high temperature.

cis-rich **P7a**: ^{1}H NMR (400 MHz, $CDCl_3$) δ 5.43–5.31 (m, 2 H), 3.93–3.83 (p, 1 H), 2.10–1.95 (m, 4 H), 1.78–1.63 (m, 4 H), 1.63–1.48 (m, 2 H), 1.48–1.25 (m, 6 H) ppm.

^{13}C NMR (101 MHz, $CDCl_3$) δ 130.4*, 129.9, 64.3, 38.6, 38.5*, 32.6*, 29.4, 29.3*, 27.3, 26.4, 26.2* ppm.

* = peaks corresponding to the *trans* signals.

trans-rich **P7a**: ^{1}H NMR (400 MHz, $CDCl_3$): 5.45–5.31 (m, 2 H), 3.93–3.83 (p, 1 H), 2.08–1.93 (m, 4 H), 1.77–1.63 (m, 4 H), 1.61–1.48 (m, 2 H), 1.47–1.24 (m, 6 H) ppm.

^{13}C NMR (101 MHz, $CDCl_3$): 130.4, 129.9, 64.4, 64.3, 38.6, 38.5, 32.6, 29.4, 29.3, 27.3, 26.4, 26.2 ppm.

Table S14. Polymerization Results for **P7b**

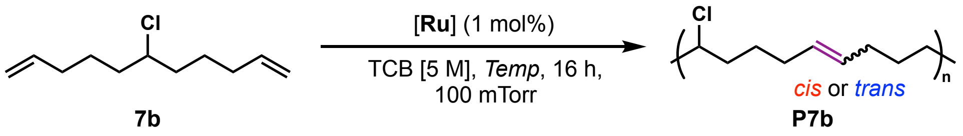

Entry	catalyst	T (°C)	M_n (kg/mol)	*Đ*	*cis* (%)
**1**	**Ru-1**	40[a]	12.7	1.76	12
**2**	**Ru-3b**	23	7.2	1.56	97

[a]Reaction temperature lowered due to the volatility of **7b** under vacuum at high temperature.

cis-rich **P7b**: ^{1}H NMR (400 MHz, $CDCl_3$) δ 5.42–5.32 (m, 2 H), 3.94–3.84 (p, 1 H), 2.13–1.96 (m, 4 H), 1.80–1.66 (m, 4 H), 1.66–1.53 (m, 2 H), 1.53–1.40 (m, 2 H) ppm.

^{13}C NMR (101 MHz, $CDCl_3$) δ 130.4*, 129.9, 64.1, 38.2, 38.1*, 32.2*, 26.8, 26.7, 26.5* ppm.

* = peaks corresponding to the *trans* signals.

The spectroscopic data for the *trans*-rich **P7b** were identical to those previously reported in the literature.[8]

trans-rich **P7b**: ^{1}H NMR (400 MHz, $CDCl_3$) δ 5.46–5.32 (m, 2 H), 3.94–3.84 (p, 1 H), 2.13–1.92 (m, 4 H), 1.80–1.66 (m, 4 H), 1.66-1.52 (m, 2 H), 1.52-1.38 (m, 2 H) ppm

Table S15. Polymerization Results for **P8a**

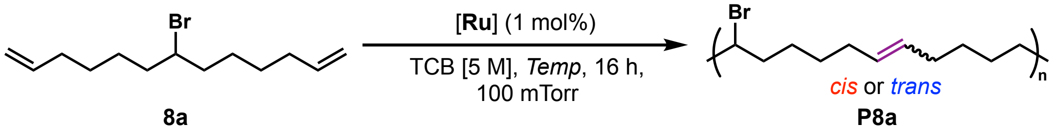

Entry	catalyst	T (°C)	M_n (kg/mol)	$Đ$	*cis* (%)
**1**	**Ru-1**	40[a]	13.4	2.30	9
**2**	**Ru-3b**	23	7.8	1.76	85

[a]Reaction temperature lowered due to the volatility of **8a** under vacuum at high temperature.

cis-rich **P8a**: ^{1}H NMR (400 MHz, $CDCl_3$) δ 5.42–5.32 (m, 2 H), 4.06–3.98 (p, 1 H), 2.11–1.94 (m, 4 H), 1.90–1.73 (m, 4 H), 1.66–1.50 (m, 2 H), 1.50–1.25 (m, 6 H) ppm.

^{13}C NMR (101 MHz, $CDCl_3$) δ 130.4*, 129.9, 58.9, 39.3, 39.2*, 32.5*, 29.3, 29.2*, 27.5, 27.2 ppm.

* = peaks corresponding to the *trans* signals.

trans-rich **P8a**: ^{1}H NMR (400 MHz, $CDCl_3$): 5.46–5.29 (m, 2 H), 4.08–3.97 (p, 1 H), 2.11–1.91 (m, 4 H), 1.90–1.72 (m, 4 H), 1.63–1.48 (m, 2 H), 1.48–1.26 (m, 6 H) ppm.

^{13}C NMR (101 MHz, $CDCl_3$): 130.4, 129.9, 59.0, 58.9, 39.3, 39.2, 32.5, 29.3, 29.2, 27.5, 27.2 ppm.

Table S16. Polymerization Results for **P8b**

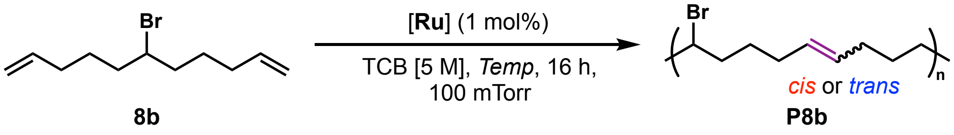

Entry	catalyst	T (°C)	M_n (kg/mol)	$Đ$	*cis* (%)
**1**	**Ru-1**	40[a]	8.5	1.96	14
**2**	**Ru-3b**	23	6.8	1.65	90

[a]Reaction temperature lowered due to the volatility of **8b** under vacuum at high temperature.

cis-rich **P8b**: ^{1}H NMR (400 MHz, $CDCl_3$) δ 5.45–5.32 (m, 2 H), 4.07–3.97 (p, 1 H), 2.16–1.93 (m, 4 H), 1.90–1.75 (m, 4 H), 1.70–1.55 (m, 2 H), 1.55–1.41 (m, 2 H) ppm.

^{13}C NMR (101 MHz, $CDCl_3$) δ 130.4*, 129.9, 58.6, 58.5, 38.8, 38.7*, 32.1*, 27.7, 27.6*, 26.7 ppm.

* = peaks corresponding to the *trans* signals.

The spectroscopic data for the *trans*-rich **P8b** were identical to those previously reported in the literature.[9]

trans-rich **P8b**: ^{1}H NMR (400 MHz, $CDCl_3$) δ 5.45–5.32 (m, 2 H), 4.07–3.97 (p, 1 H), 2.13–1.93 (m, 4 H), 1.89–1.74 (m, 4 H), 1.69–1.55 (m, 2 H), 1.55–1.40 (m, 2 H) ppm.

Table S17. Polymerization Results for **P9a**

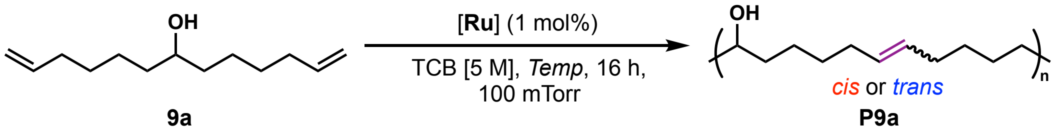

Entry	catalyst	T (°C)	M_n (kg/mol)	$Đ$	*cis* (%)
**1**	**Ru-1**	80	4.7	1.49	12
**2**	**Ru-3b**	23	4.8	1.47	93

NOTE: No antisolvent could be identified to purify *cis* and *trans* **P9a** through precipitation likely due to their low molar masses.

cis-rich **P9a**: ^{1}H NMR (400 MHz, CDC13): 5.43–5.29 (m, 2H), 3.65–3.49 (br, 1 H), 2.09–1.93 (m, 4H), 1.89–1.68 (br, 1 H), 1.54–1.25 (m, 12 H) ppm.

^{13}C NMR (101 MHz, CDCl3): 130.5*, 130.0, 71.9, 37.5, 37.4*, 32.6*, 29.9, 29.7*, 27.3, 25.4, 25.2* ppm.

* = peaks corresponding to the *trans* signals.

trans-rich **P9a**: ^{1}H NMR (400 MHz, CDCl3): 5.43–5.29 (m, 2 H), 3.65–3.49 (br, 1 H), 2.09–1.93 (m, 4 H), 1.89–1.66 (br, 1 H), 1.65–1.20 (m, 12 H) ppm.

^{13}C NMR (101 MHz, CDCl3): 130.5, 130.0, 71.9, 37.5, 37.4, 32.6, 29.9, 29.7, 27.3, 25.4, 25.2 ppm.

Table S18. Polymerization Results for **P9b**

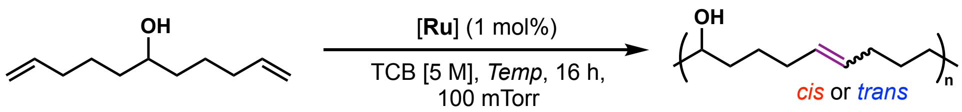

Entry	catalyst	T (°C)	M_n (kg/mol)	*Đ*	*cis* (%)
**1**	**Ru-3b**	23	2.4	1.40	96
**2**	**Ru-1**	80	6.5	1.43	15

NOTE: No antisolvent could be identified to purify *cis* and *trans* **P9a** through precipitation likely due to their low molar masses.

cis-rich **P9b**: ^{1}H NMR (400 MHz, $CDCl_3$) δ 5.48–5.31 (m, 2 H), 3.65–3.52 (br, 1 H), 2.16–1.94 (m, 4 H), 1.91–1.66 (br, 1 H), 1.63–1.22 (m, 8 H) ppm.

^{13}C NMR (101 MHz, $CDCl_3$): 130.5*, 130.0, 71.7, 71.6, 37.2, 37.0*, 32.6*, 27.3, 25.9, 25.7* ppm.

* = peaks corresponding to the *trans* signals.

The spectroscopic data for the *trans*-rich **P9b** were identical to those previously reported in the literature.[10]

trans-rich **P9b**: ^{1}H NMR (400 MHz, $CDCl_3$): 5.48–5.31 (m, 2 H), 3.65–3.52 (br, 1 H), 2.16–1.94 (m, 4 H), 1.91–1.66 (br, 1 H), 1.63–1.22 (m, 8 H) ppm.

***cis*-Selective ADMET Polymerization Procedure Using an N_2 Flow for Monomer P5**

In an N_2-filled glovebox, deca-1,9-diene (69 mg, 0.5 mmol, 1 equiv.) was weighed into a vial equipped with a stir bar. Then, **Ru-3b** (3.4 mg, 0.005 mmol, 0.01 equiv.) was weighed into a separate vial and dissolved in TCB (0.25 mL). The catalyst solution was taken up in the syringe and ejected back into the vial three times to ensure the catalyst dissolved. The catalyst solution was then transferred to the monomer vial where the solution was taken up in the syringe and ejected three times. The vial was then capped with a screw cap fitted with a Teflon septa and removed from the box. The vial was taken to a Schlenk line where it was pierced with a 3” 18 G pink needle with a positive N_2 flow until it made contact with the bottom of the vial. An 18 G needle was then pierced through the septa as a bleed needle and the reaction mixture was checked to make sure proper bubbling of N_2 was occurring. The reaction was stirred at 100 rpm at room temperature for 48 h. At this point, 50 μL of ethyl vinyl ether was added to quench the polymerization for 5 minutes. The mixture was then dissolved in the minimum amount of DCM (~ 1 mL) and precipitated in MeOH in a centrifuge tube. The mixture then underwent centrifugation at 8500 rpm for 10 minutes. The MeOH was decanted, the polymer was redissolved in DCM, and precipitated in MeOH again. After a second centrifugation cycle, the MeOH was decanted and **P5** was dried for 24 h under vacuum.

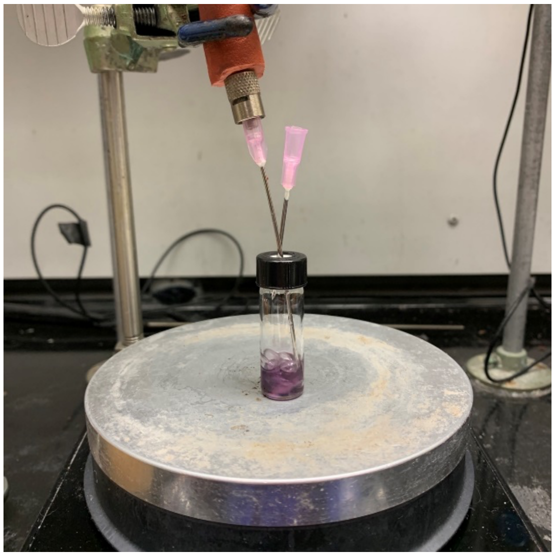

Figure S4. Purging reaction setup for polymer synthesis with **Ru-3b**.

Table S19. Polymerization Results for **P5**

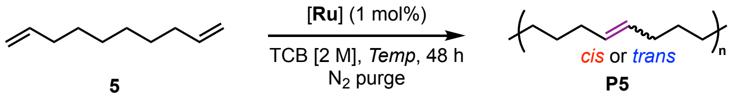

Entry	catalyst	T (°C)	M_n (kg/mol)	*Đ*	*cis* (%)
**1**	**Ru-1**	90	10.2	1.78	16
**2**	**Ru-3b**	23	8.5	1.80	99

The spectroscopic data for the *cis*-rich **P5** were identical to those previously reported in the literature.[12]

cis-rich **P5**: ^{1}H NMR (400 MHz, $CDCl_3$) δ 5.41–5.28 (m, 2 H), 2.10–1.93 (m, 4 H), 1.40–1.23 (m, 8 H) ppm.
^{13}C NMR (101 MHz, $CDCl_3$) δ 130.0, 29.9, 29.4, 27.4 ppm.

The spectroscopic data for the *trans*-rich **P5** were identical to those previously reported in the literature.[13]
trans-rich **P5**: ^{1}H NMR (400 MHz, $CDCl_3$) δ 5.53–5.27 (m, 2H), 2.06–1.90 (m, 4H), 1.40–1.20 (m, 8H) ppm.

Triblock Copolymer Synthesis

P5$_{cis}$-OAc (99% *cis*)

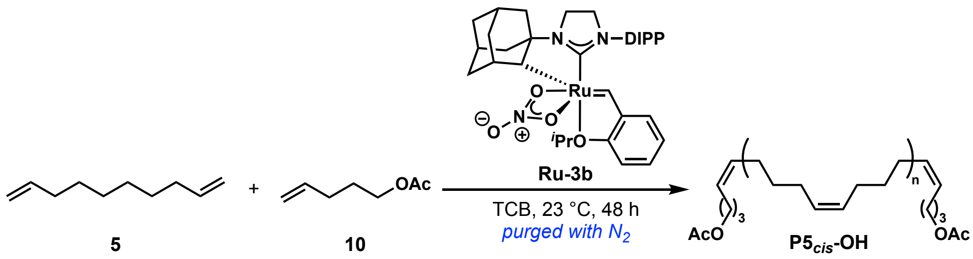

In a nitrogen-filled glove box, an oven-dried vial was charged with **Ru-3b** (5.0 µmol, 0.01 equiv) and TCB (0.25 mL). The solution of catalyst was added to another oven-dried vial containing pre-

weighed monomer **5** (0.50 mmol, 1 equiv), acetate reagent **10** (0.10 mmol, 0.2 equiv), and a stir bar. The vial capped with a Teflon cap was then removed from the glove box and stirred for 48 h under a constant nitrogen flow passing through a 3-inch, 18G needle with another 18G bleed needle (Figure S2). Polymer **P5$_{cis}$-OAc** was then precipitated with ice-cold methanol, isolated via centrifugation (8500 rpm, 10 min), and dried under high vacuum for 16–24 h.

^{1}H NMR (400 MHz, $CDCl_3$) δ 5.35 (t, J = 4.7 Hz, 44 H), 4.06 (t, J = 6.7 Hz, 4 H), 2.06–1.95 (m, 92 H), 1.32 (d, J = 10.4 Hz, 178 H) ppm.
^{13}C NMR (101 MHz, $CDCl_3$) δ 129.9, 29.7, 29.2, 27.2 ppm.

P5$_{trans}$-OAc (89% *trans*)

In a nitrogen-filled glove box, an oven-dried vial was charged with **Ru-1** (5.0 μmol, 0.01 equiv) and dissolved in TCB (0.25 mL). The solution of catalyst was added to another vial containing the pre-weighed monomer **5** (0.50 mmol, 1 equiv), acetate reagent **10** (0.10 mmol, 0.2 equiv) and a stir bar. The vial capped with a Teflon cap was then removed from the glove box. The reaction was placed in a pre-heated oil bath (90 °C) and stirred for 48 h under a constant nitrogen flow passing through one 3-inch, 18G needle and an 18G bleed needle (Figure S2). Polymer **P5$_{trans}$-OAc** was then precipitated with ice-cold methanol, isolated via centrifugation (8500 rpm, 10 min), and then dried under high vacuum for 16–24 h.

^{1}H NMR (400 MHz, $CDCl_3$) δ 5.44–5.29 (m, 50 H), 4.06 (t, J = 6.7 Hz, 4 H), 2.03–1.89 (m, 96 H), 1.38–1.24 (m, 195 H) ppm.
^{13}C NMR (101 MHz, $CDCl_3$) δ 130.3, 129.9, 32.6, 29.7, 29.1, 27.2 ppm.

Hydrolysis of **P5$_{cis}$-OAc** (99% *cis*) and **P5$_{trans}$-OAc** (89% *trans*)

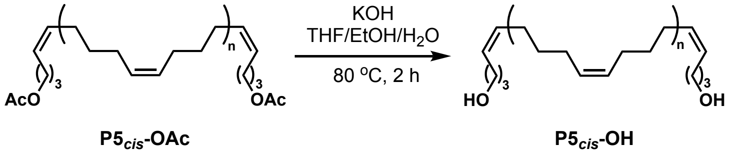

P5$_{cis}$-OAc (or **P5$_{trans}$-OAc**) (30 mg) was placed in a reaction vial equipped with a stir bar, and solution of KOH (100 mg) in a THF:EtOH:H_2O mixture (1:1:1, 6 mL) was added to the vial. The polymer suspension was heated to 80 °C for 2 h. The reaction was cooled to room temperature and diluted with dichloromethane (20 mL). The organic layer was washed with H_2O (10 mL), dried

over sodium sulfate, and then the solvent was removed *in vacuo* affording **P5$_{cis}$-OH** (or **P5$_{trans}$-OH**).

P5$_{cis}$-OH (99% *cis*)

^{1}H NMR (400 MHz, $CDCl_3$) δ 5.35 (t, *J* = 4.7 Hz, 50 H), 3.66 (t, *J* = 6.5 Hz, 4 H), 2.09–1.92 (m, 101 H), 1.39–1.24 (m, 211 H) ppm.

^{13}C NMR (101 MHz, $CDCl_3$) δ 129.9, 29.7, 29.2, 27.2 ppm.

P5$_{trans}$-OH (89% *trans*)

^{1}H NMR (400 MHz, $CDCl_3$) δ 5.45–5.30 (m, 54 H), 3.65 (t, *J* = 6.3 Hz, 4 H), 2.12–1.87 (m, 110 H), 1.44–1.20 (m, 216 H) ppm.

^{13}C NMR (101 MHz, $CDCl_3$) δ 130.3, 129.9, 32.6, 29.7, 29.6, 29.1, 29.1, 27.2 ppm.

Synthesis of **P11**-*b*-**P5$_{cis}$**-*b*-**P11** (99% *cis*) and **P11**-*b*-**P5$_{trans}$**-*b*-**P11** (89% *trans*)

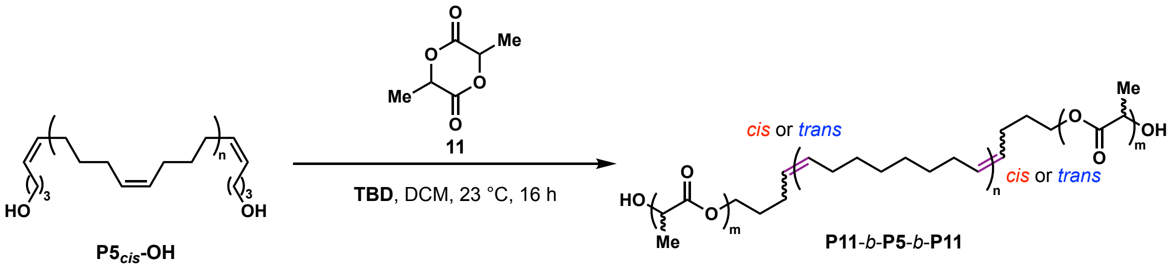

In a nitrogen-filled glove box, D,L-lactide (59.2 mg, 411 µmol, 120 equiv) was added to vial containing anhydrous DCM (0.3 mL). The solution was then transferred to a vial containing pre-weighed **P5-OH** (3.4 µmol, 1 equiv) and a stir bar. The mixture was stirred for 5 min followed by rapid addition of a solution of triazabicyclodecene (TBD, 0.376 mg, 2.7 µmol, 0.8 equiv) in anhydrous DCM (0.1 mL) with a syringe. The vial was then removed from the glovebox, and parafilm was wrapped around the cap to ensure an optimum seal. The mixture was stirred for 16 h at room temperature, then quenched by addition of an excess of acetic acid (0.1 mL). The polymer solution was precipitated by addition into methanol, and the solid was isolated by centrifugation and decantation. The resulting ABA triblock copolymer was dried under high vacuum.

P11-*b*-P5$_{cis}$-*b*-P11 (99% *cis*)

^{1}H NMR (400 MHz, $CDCl_3$) δ 5.40–5.29 (m, 44 H), 5.29–5.09 (m, 231 H), 2.11–1.90 (m, 82 H), 1.68–1.47 (m, 736 H), 1.38–1.25 (m, 209 H) ppm.

^{13}C NMR (101 MHz, $CDCl_3$) δ 169.3, 129.9, 69.0, 29.7, 29.2, 27.2, 16.6 ppm.

P11-*b*-P5*trans*-*b*-P11 (89% *trans*)

^{1}H NMR (400 MHz, $CDCl_3$) δ 5.40–5.22 (m, 44 H), 5.20–5.02 (m, 242 H), 2.03–1.81 (m, 83 H), 1.56–1.42 (m, 757 H), 1.32–1.16 (m, 171 H) ppm.

^{13}C NMR (101 MHz, $CDCl_3$) δ 169.4, 130.4, 69.4, 69.1, 32.6, 29.6, 16.7 ppm.

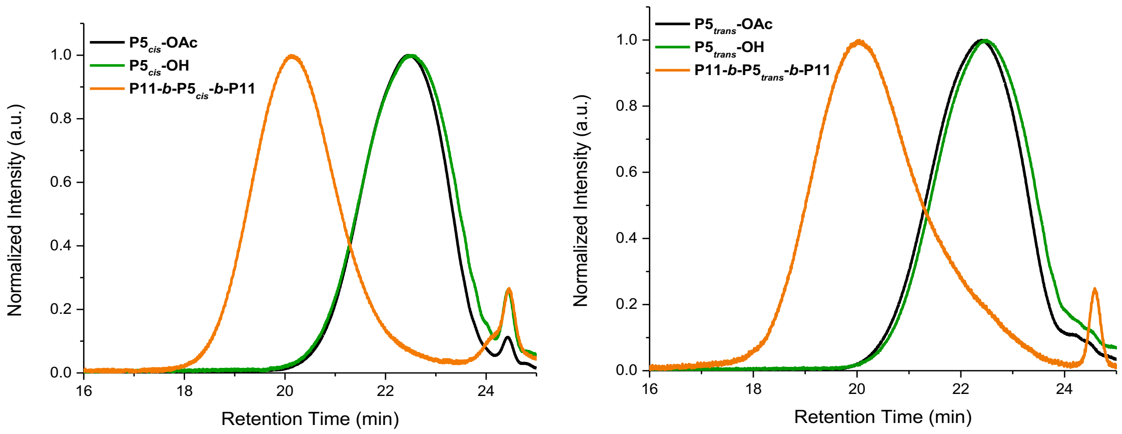

Figure S5. SEC traces showing formation of **P11-*b*-P5*cis*-*b*-P11** (left, orange) from **P5*cis*-OH** (green) and formation of **P11-*b*-P5*trans*-*b*-P11** (right, orange) from **P5*trans*-OH** (green).

NMR Deconvolution

Due to overlap in the ^{1}H NMR spectrum between the *cis* and *trans* alkene signals, the built-in MestReNova Global Spectra Deconvolution (GSD) software was used to calculate the *cis* content. The following equation was used where the letter refers to the area under the specified curve:

$$cis\% = \left[1 - \left(\frac{\sum A:C}{\sum A:F}\right)\right] * 100 \qquad (1)$$

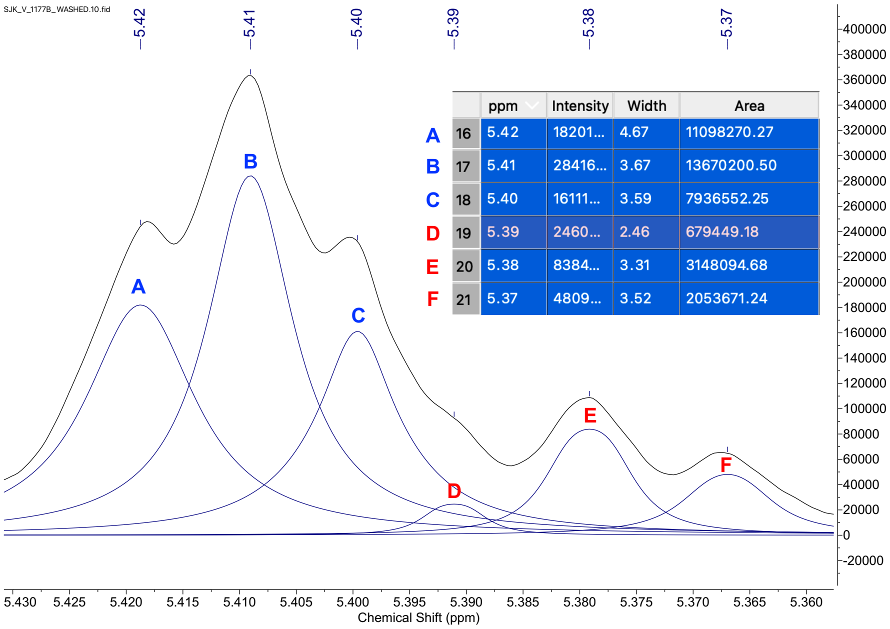

Figure S6. ^{1}H NMR spectral deconvolution of **P1a** formed by **Ru-1** at 80 °C (15% *cis*).

Thermal Characterization Data

Table S20. Summary of DSC and TGA Data for **P-1–P-8**

**Entry**	**Polymer**	M_n (kg/mol)	*Đ*	*cis* (%)	T_d (°C)	T_g (°C)	T_m (°C)	T_c (°C)
**1**	**P1a**	12.0	1.60	99	334	–73	–	–
**2**	**P1a**	20.9	1.81	15	298	–68	11	–17
**3**	**P1b**	9.1	1.38	99	314	–75	–	–
**4**	**P1b**	15.7	1.80	17	253	–64	–	–
**5**	**P2a**	8.4	1.42	99	280	–	–	–
**6**	**P2a**	18.8	1.77	23	274	–	–	–
**7**	**P2b**	9.5	1.56	99	277	–	–	–
**8**	**P2b**	9.9	1.67	23	227	–	–	–

**9**	**P3a**	8.3	1.52	91	317	–29	–	–
**10**	**P3a**	7.9	2.22	25	304	–27	83	26
**11**	**P3b**	5.0	1.69	99	309	–24	–	–
**12**	**P3b**	11.2	3.23	21	273	–15	–	–
**13**	**P4a**	10.0	1.49	99	406	–	–	–
**14**	**P4a**	26.5	1.55	18	401	–	–9	–27
**15**	**P4b**	10.6	1.35	99	301	–	–	–
**16**	**P4b**	22.4	1.61	23	286	–	–	–
**17**	**P5**	8.5	1.80	99	380	–	32	16
**18**	**P5**	10.2	1.78	16	406	–	24	12
**19**	**P5$_{cis}$-OAc**	3.3	1.41	99	349	–	–9	–30, –8
**20**	**P5$_{trans}$-OAc**	3.6	1.40	11	333	–	–3, 8, 17	–6, 5
**21**	**P5$_{cis}$-OH**	3.3	1.40	99	247	–	23	–39
**22**	**P5$_{trans}$-OH**	3.1	1.52	11	295	–	45, 51	36
**23**	**P11-*b*- P5$_{cis}$-*b*-P11**	17.3	1.32	99	278	32	–	–
**24**	**P11-*b*- P5$_{trans}$-*b*-P11**	15.7	1.46	11	273	43	19	–19
**19**	**P6a**	10.3	1.45	99	340	–	–	–
**20**	**P6a**	19.1	2.02	13	344	–	–	–
**21**	**P6b**	17.6	1.29	99	310	–	–	–

**22**	**P6b**	28.9	1.40	20	307	–	–	–
**23**	**P7a**	7.3	1.64	92	329	–73	–	–
**24**	**P7a**	16.2	1.87	19	238	–67	–	–
**25**	**P7b**	7.2	1.56	97	293	–69	–	–
**26**	**P7b**	12.7	1.76	12	246	–62	–	–
**27**	**P8a**	7.8	1.76	85	250	–70	–	–
**28**	**P8a**	13.4	2.30	9	236	–63	–	–
**28**	**P8b**	6.8	1.65	90	241	–64	–	–
**29**	**P8b**	8.5	1.96	14	237	–57	–	–
**30**	**P11**	19.9	1.72	–	249	49	–	–

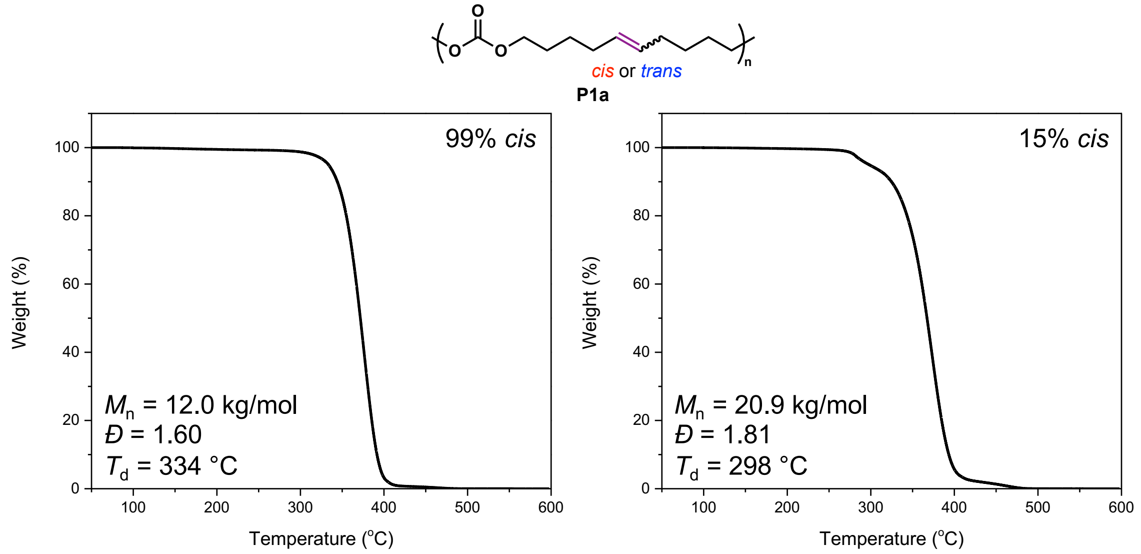

Figure S7. TGA thermograms of 99% *cis* **P1a** (left) and 15% *cis* **P1a** (right).

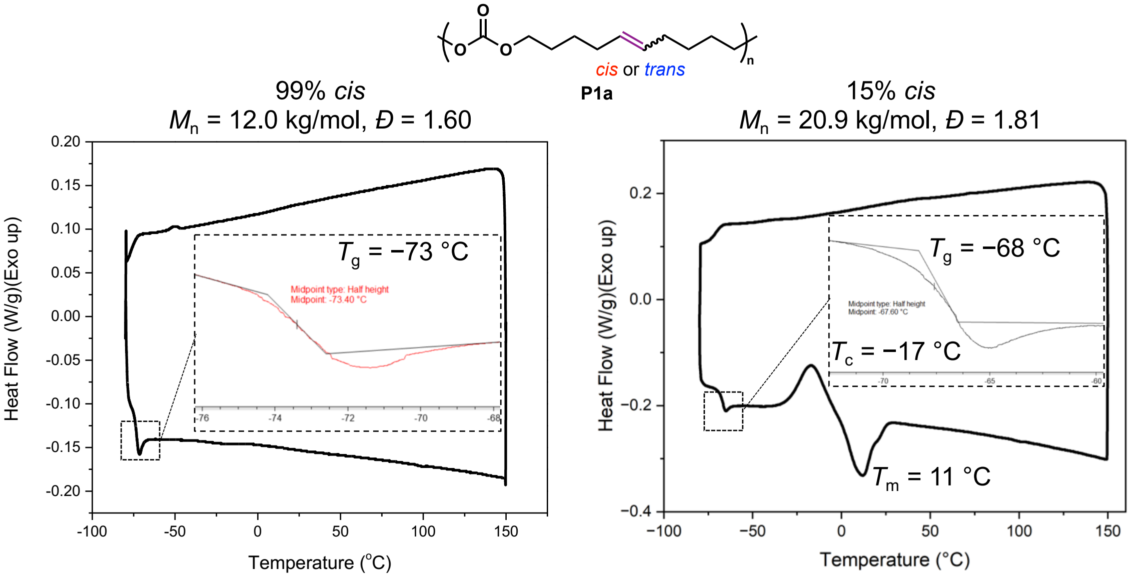

Figure S8. DSC thermograms of 99% *cis* **P1a** (left) and 15% *cis* **P1a** (right).

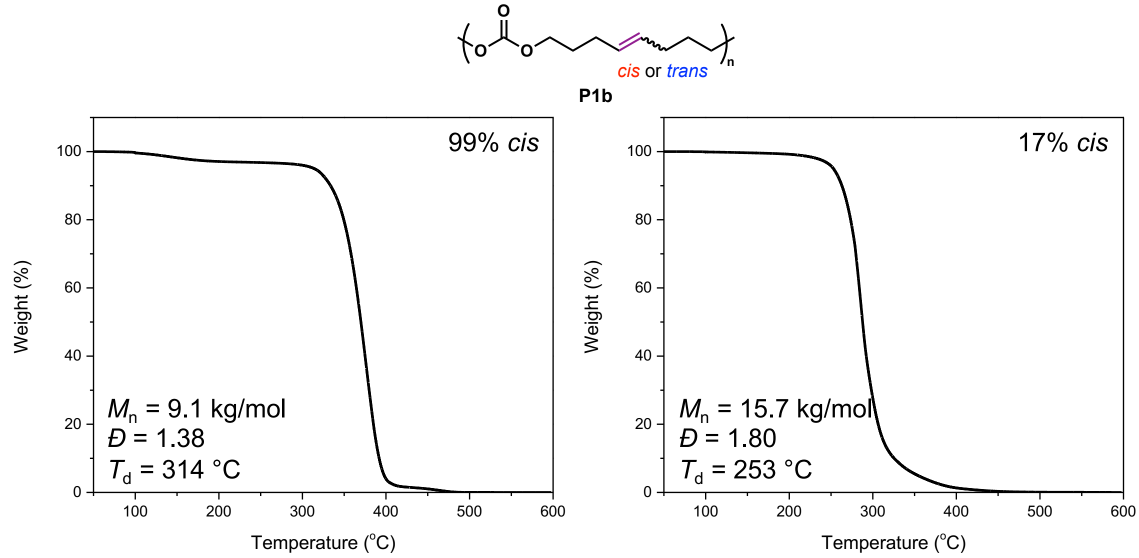

Figure S9. TGA plots of 99% *cis* **P1b** (left) and 17% *cis* **P1b** (right).

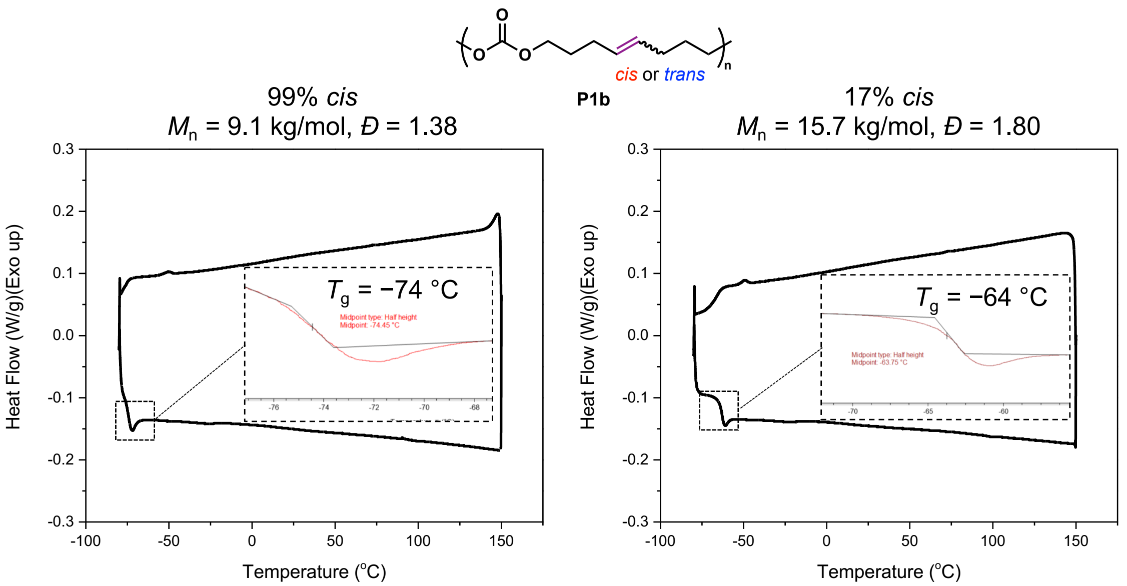

Figure S10. DSC plots of 99% *cis* **P1b** (left) and 17% *cis* **P1b** (right).

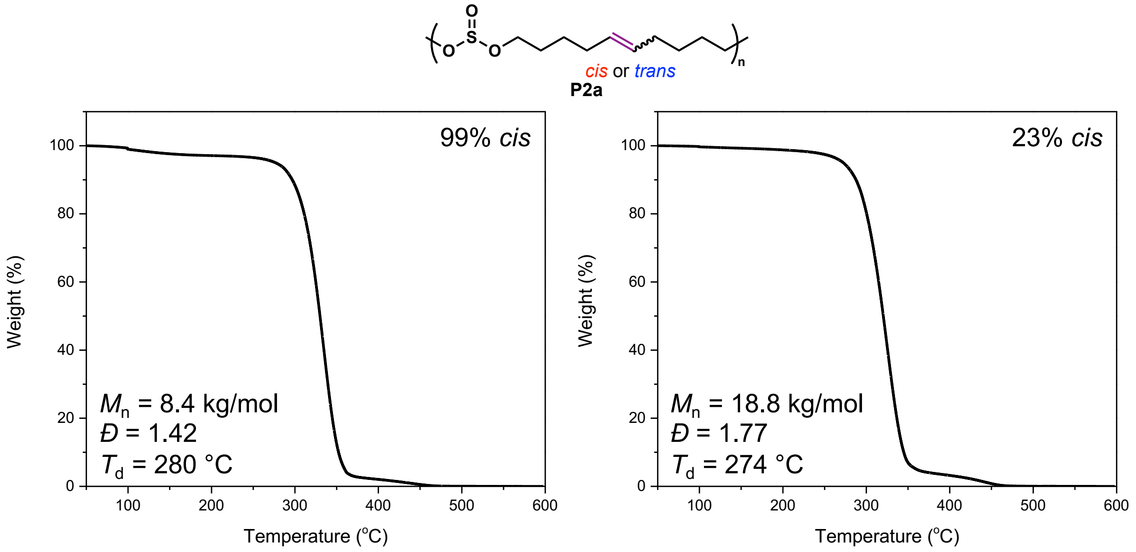

Figure S11. TGA plots of 99% *cis* **P2a** (left) and 23% *cis* **P2a** (right).

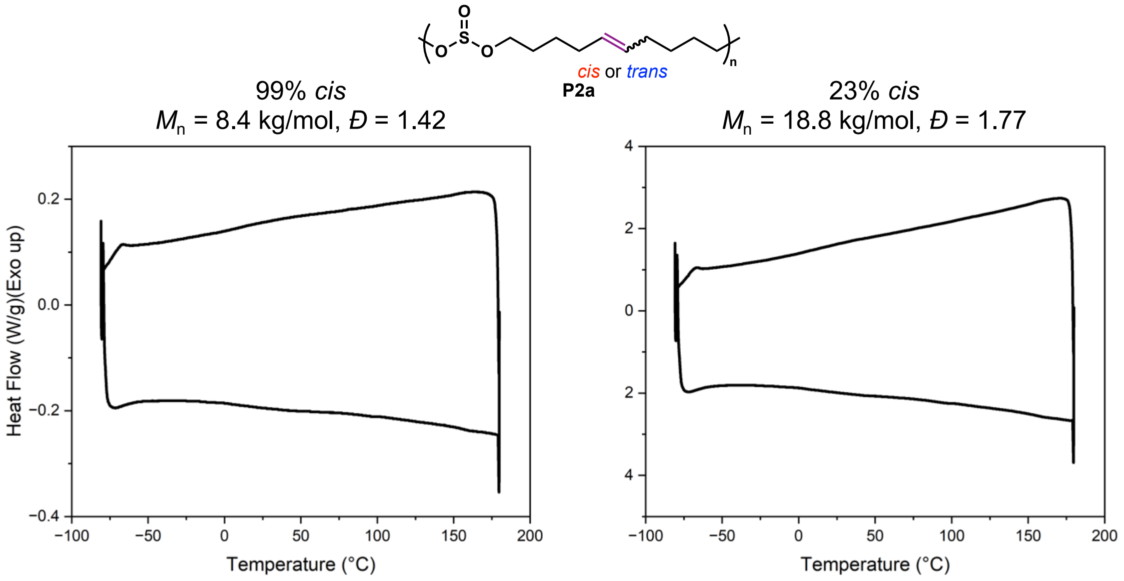

Figure S12. DSC plots of 99% *cis* **P2a** (left) and 23% *cis* **P2a** (right).

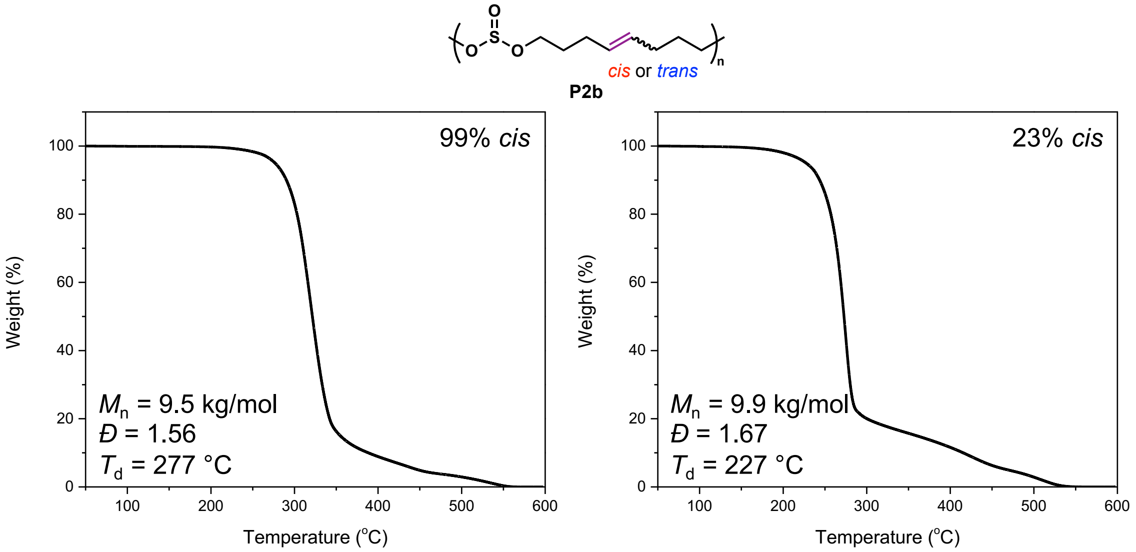

Figure S13. TGA plots of 99% *cis* **P2b** (left) and 23% *cis* **P2b** (right).

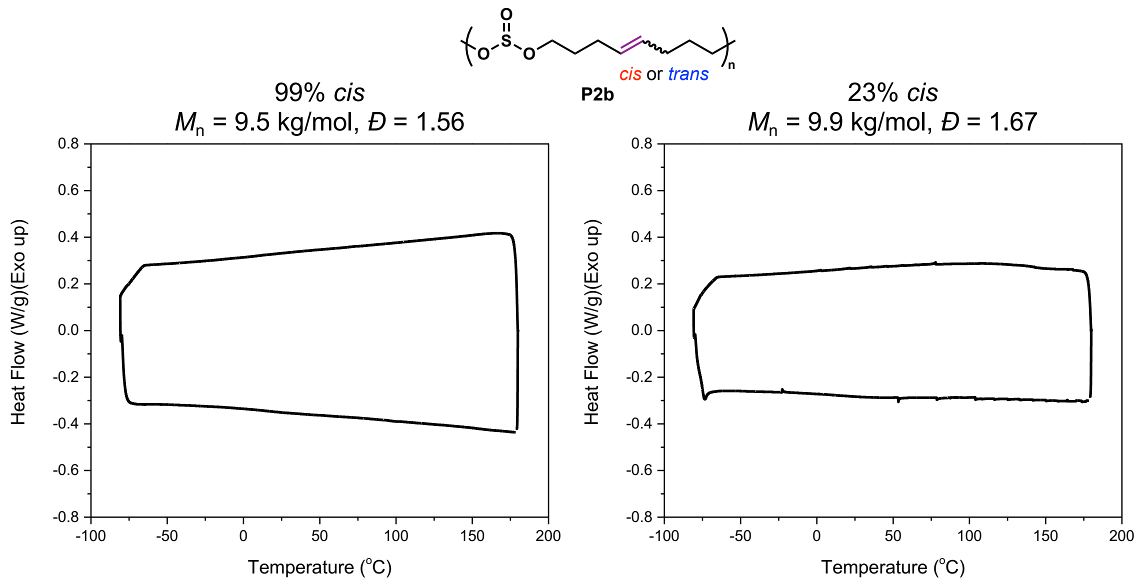

Figure S14. DSC plots of 99% *cis* **P2b** (left) and 23% *cis* **P2b** (right).

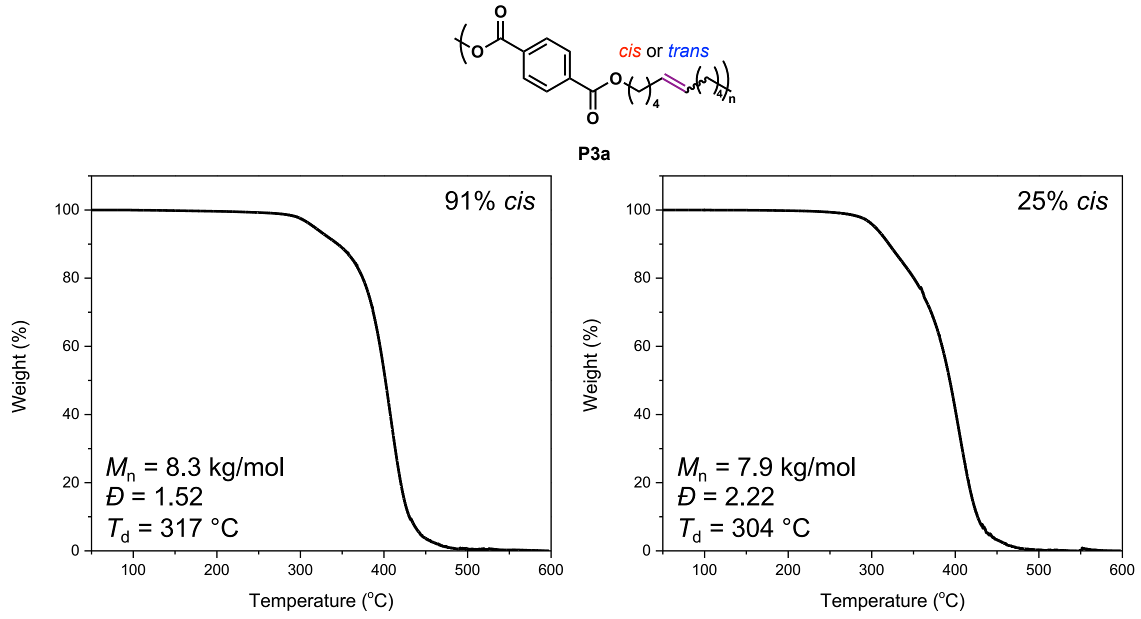

Figure S15. TGA plots of 91% *cis* **P3a** (left) and 25% *cis* **P3a** (right).

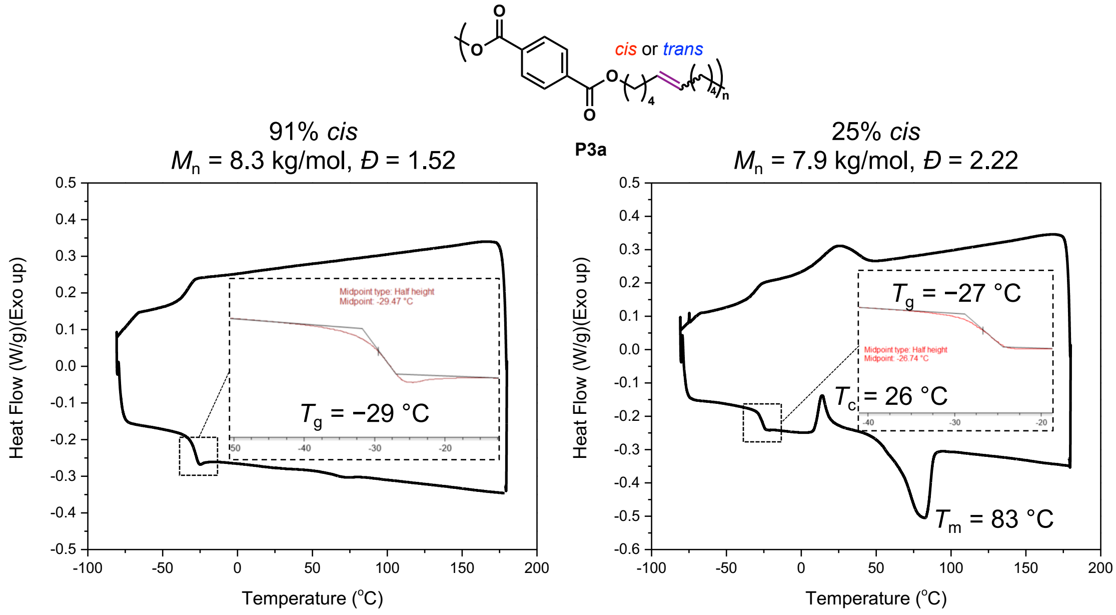

Figure S16. DSC plots of 91% *cis* **P3a** (left) and 25% *cis* **P3a** (right).

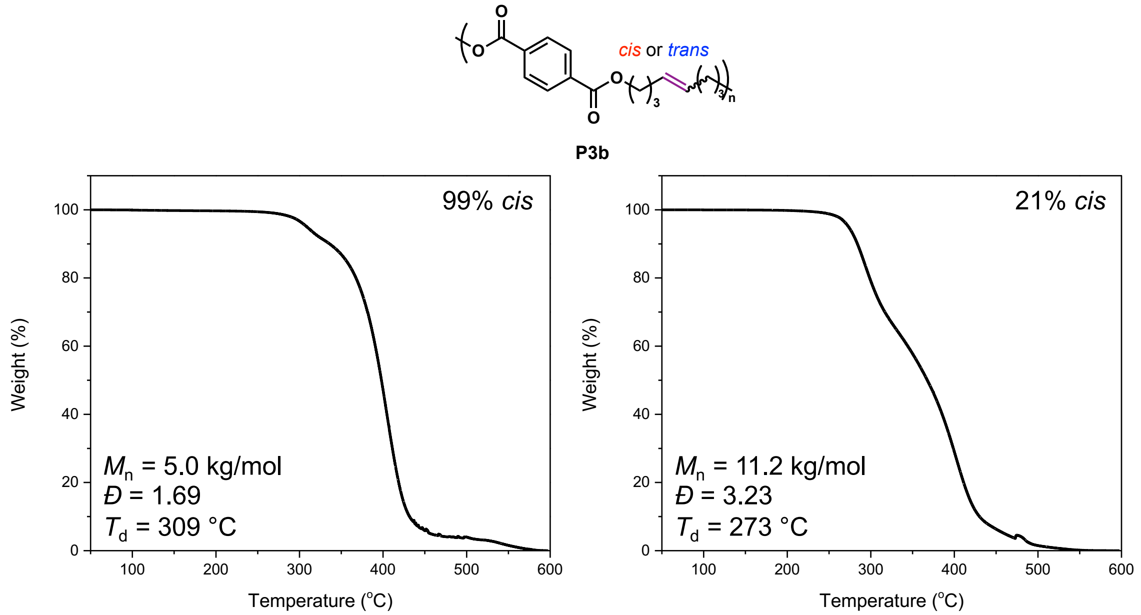

Figure S17. TGA plots of 99% *cis* **P3b** (left) and 21% *cis* **P3b** (right).

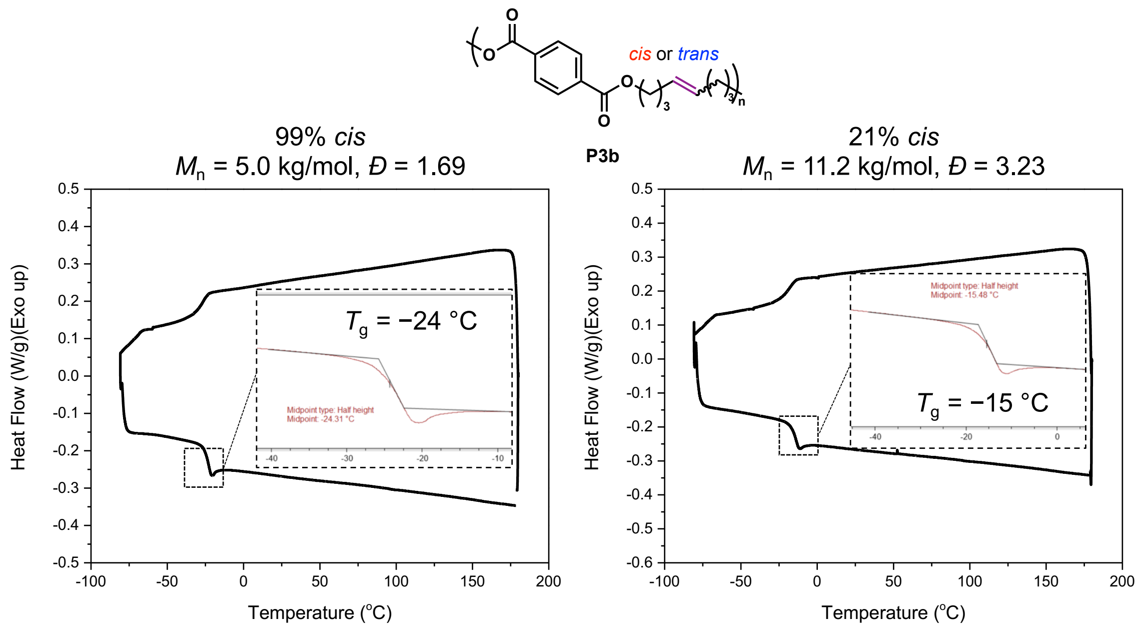

Figure S18. DSC plots of 99% *cis* **P3b** (left) and 21% *cis* **P3b** (right).

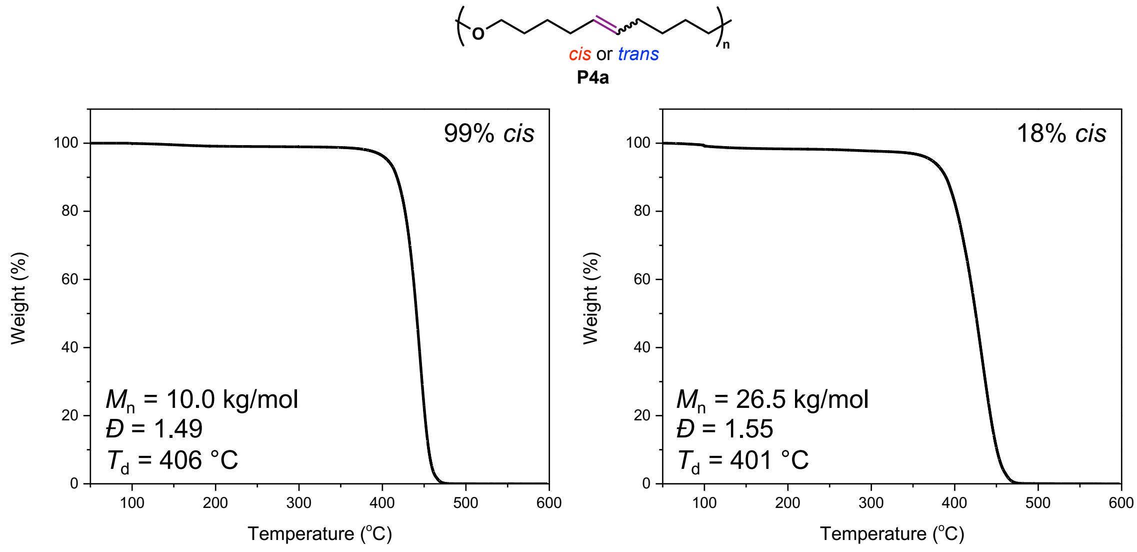

Figure S19. TGA plots of 99% *cis* **P4a** (left) and 18% *cis* **P4a** (right).

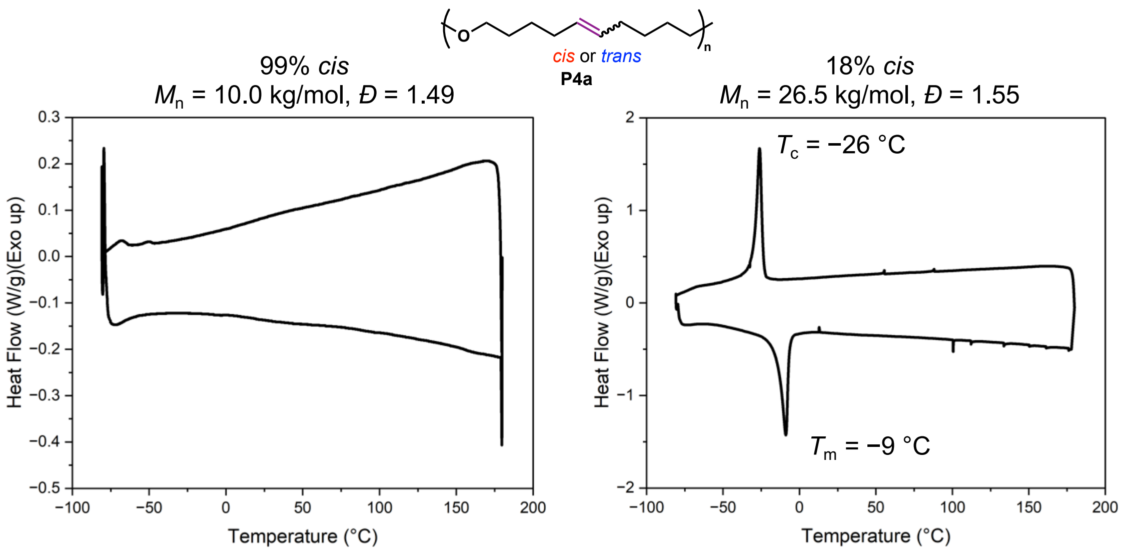

Figure S20. DSC plots of 99% *cis* **P4a** (left) and 18% *cis* **P4a** (right).

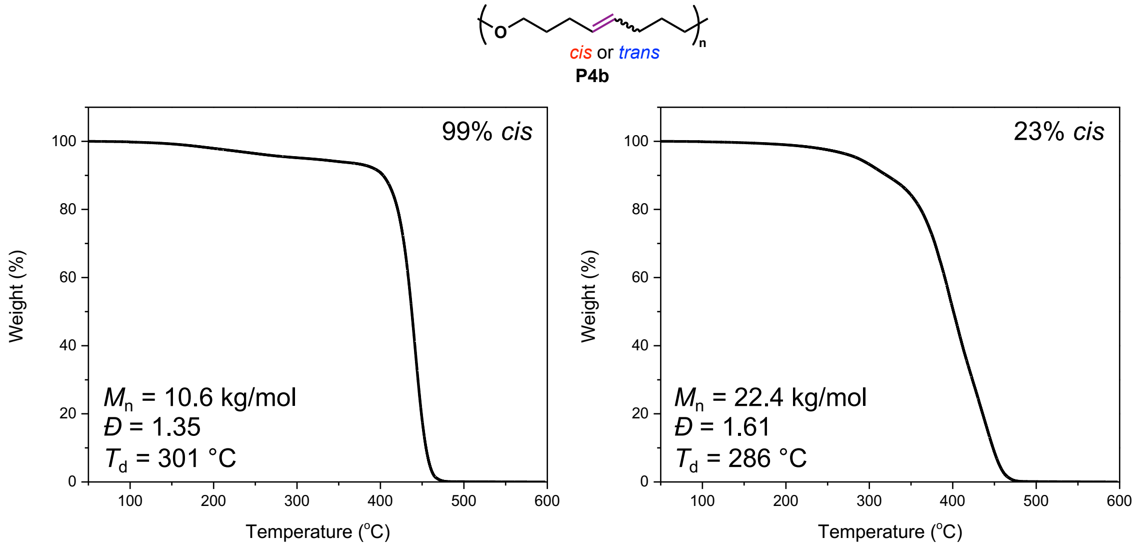

Figure S21. TGA plots of 99% *cis* **P4b** (left) and 23% *cis* **P4b** (right).

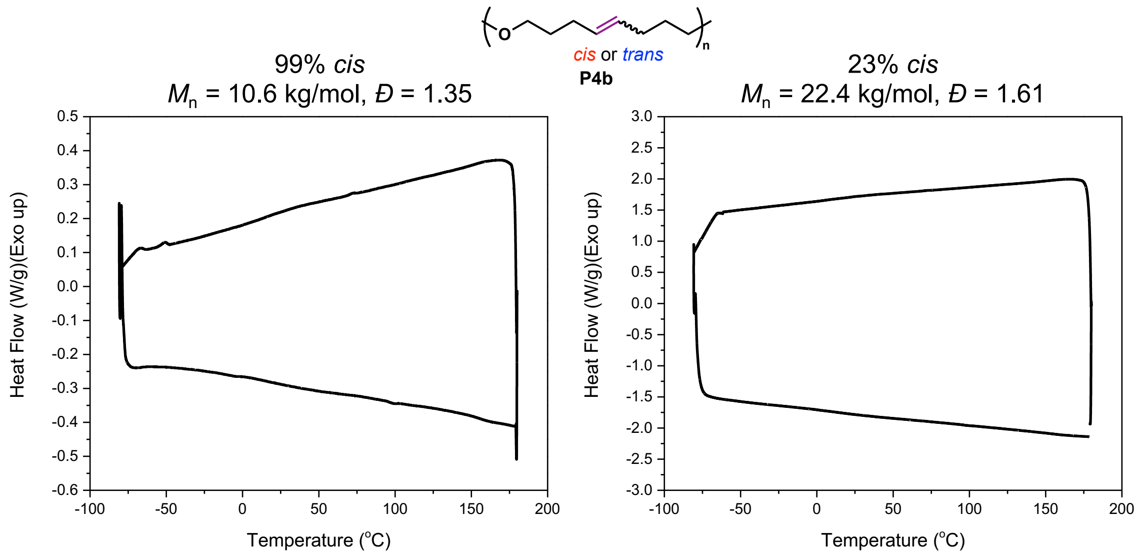

Figure S22. DSC plots of 99% *cis* **P4b** (left) and 23% *cis* **P4b** (right).

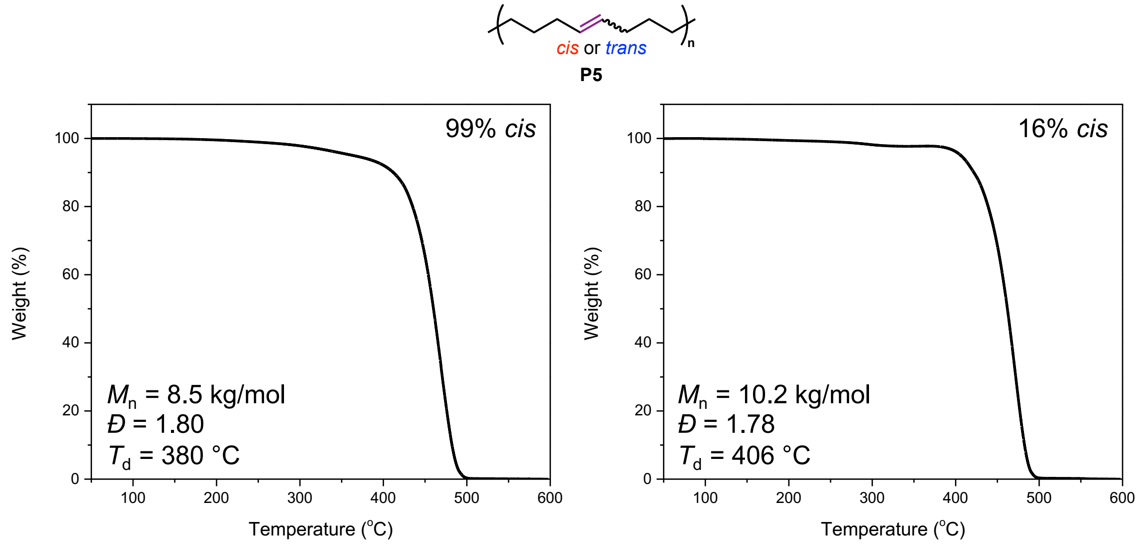

Figure S23. TGA plots of 99% *cis* **P5** (left) and 16% *cis* **P5** (right).

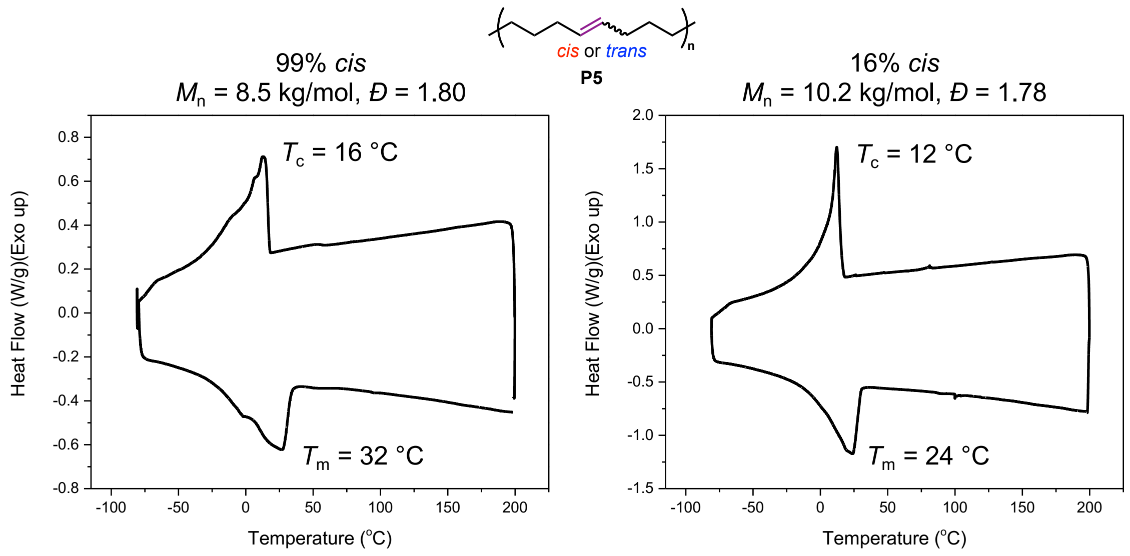

Figure S24. DSC plots of 99% *cis* **P5** (left) and 16% *cis* **P5** (right).

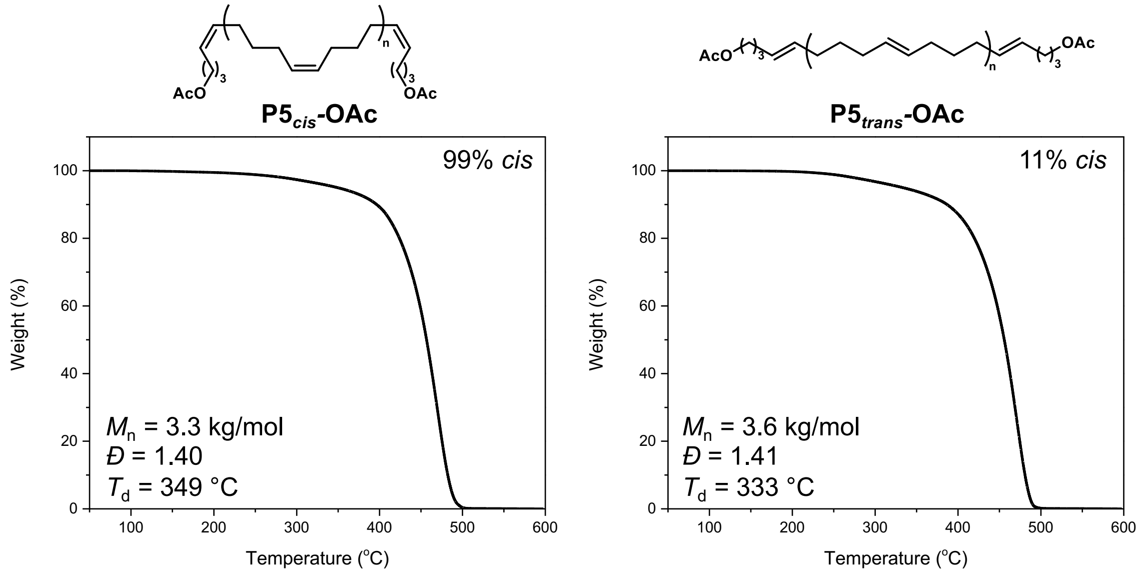

Figure S25. TGA plots of **P5$_{cis}$-OAc** (left) and **P5$_{trans}$-OAc** (right).

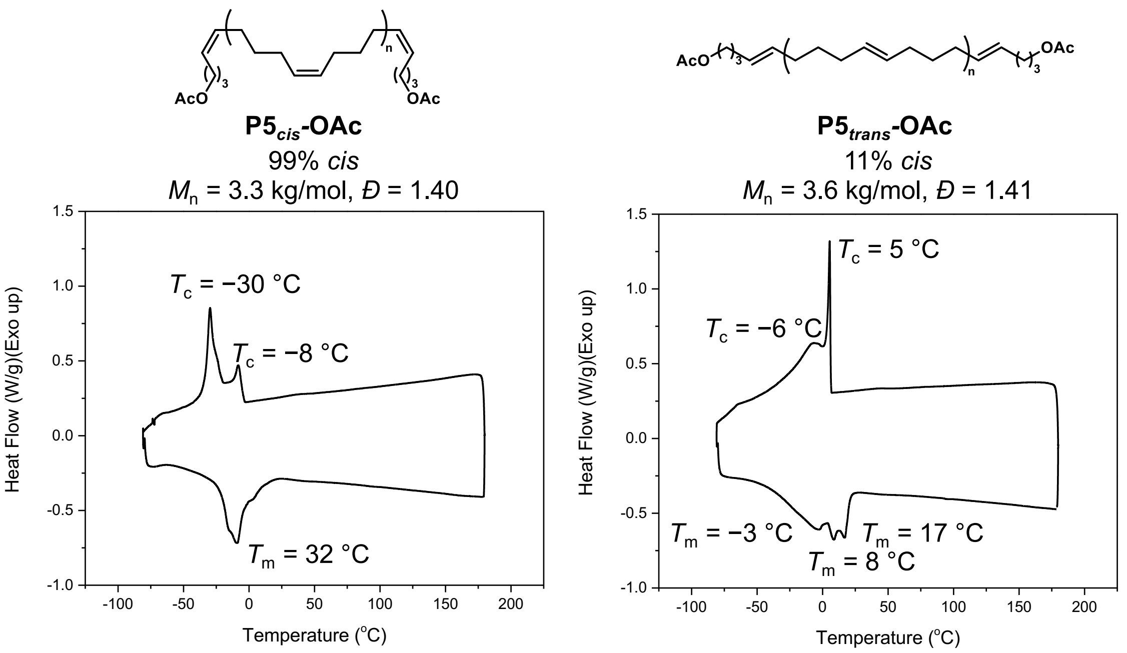

Figure S26. DSC plots of **P5$_{cis}$-OAc** (left) and **P5$_{trans}$-OAc** (right).

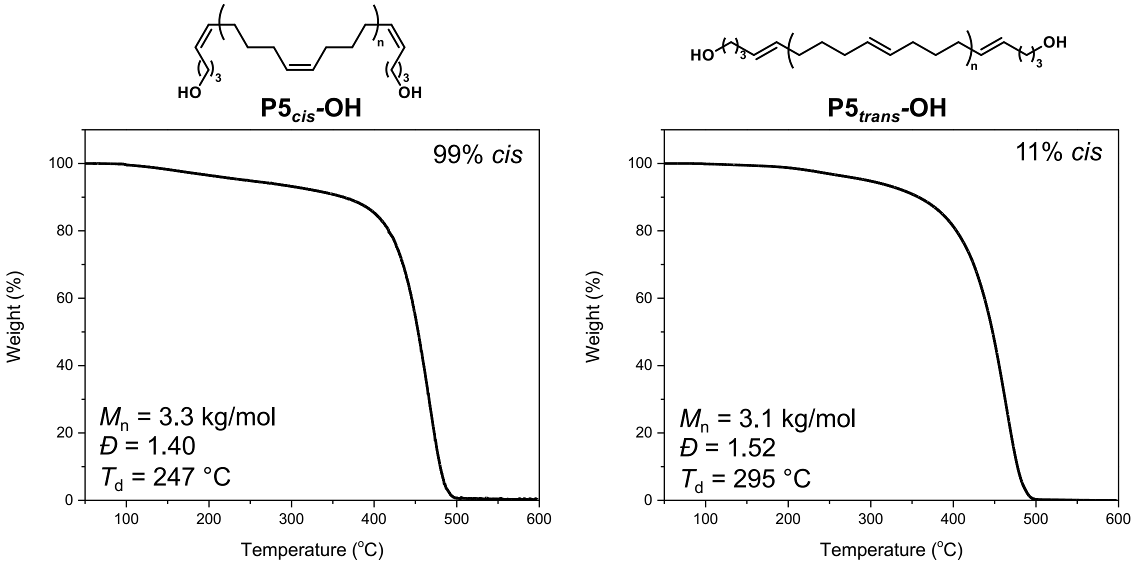

Figure S27. TGA plots of **P5$_{cis}$-OH** (left) and **P5$_{trans}$-OH** (right).

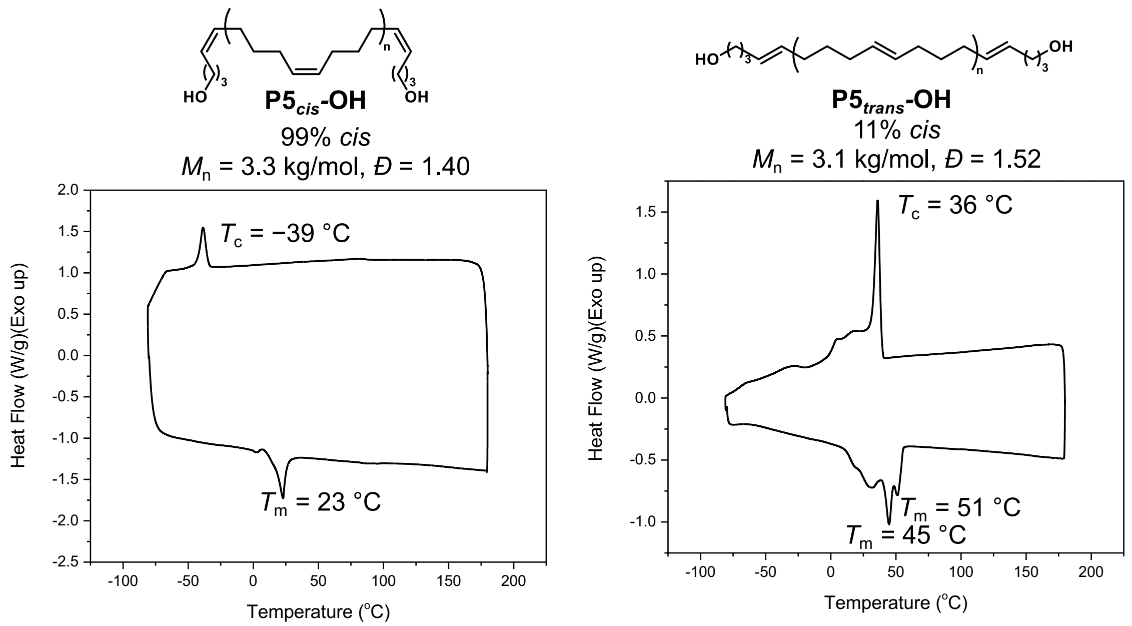

Figure S28. DSC plots of **P5$_{cis}$-OH** (left) and **P5$_{trans}$-OH** (right).

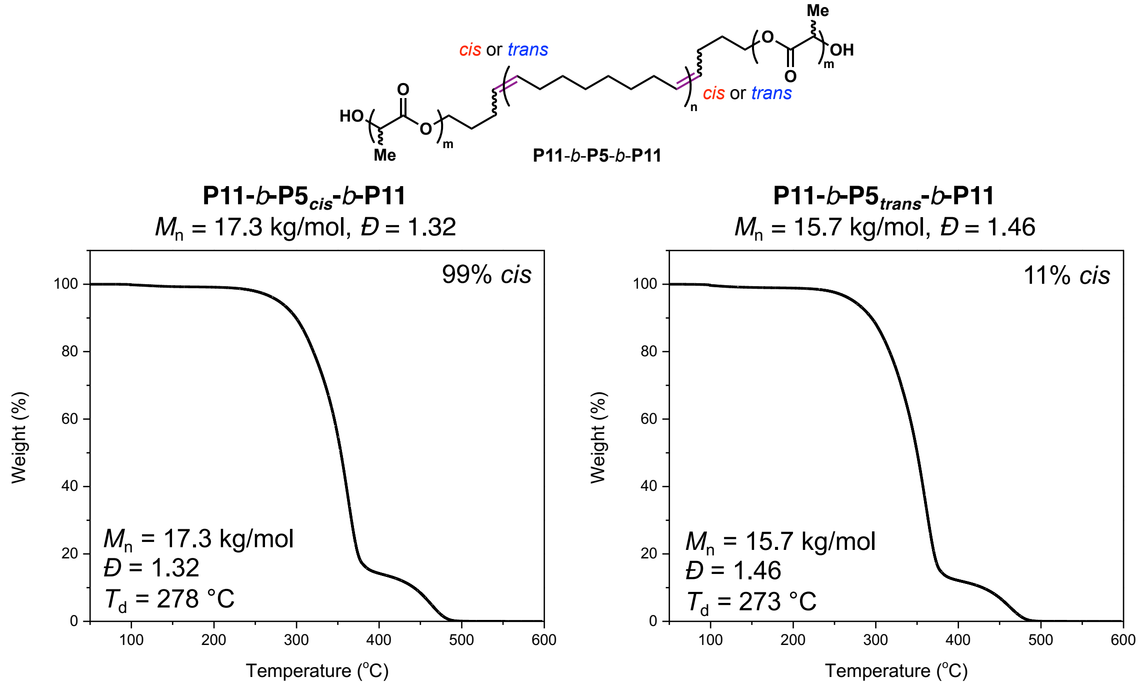

Figure S29. TGA plots of **P11-*b*-P5$_{cis}$-*b*-P11** (left) and **P11-*b*-P5$_{trans}$-*b*-P11** (right).

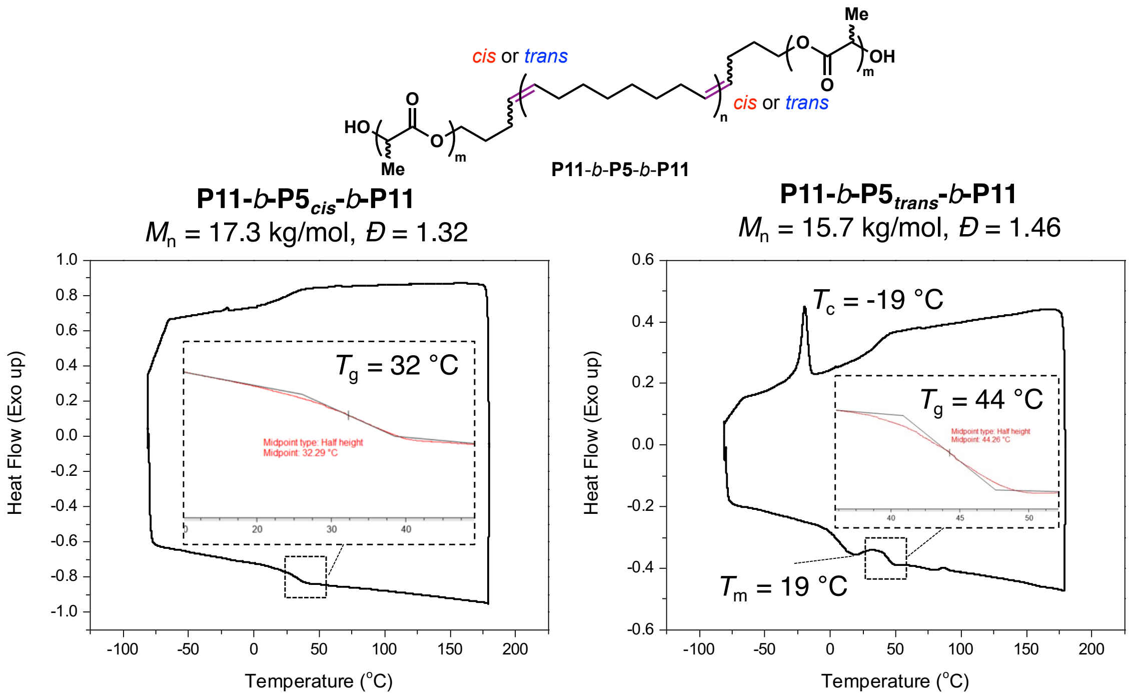

Figure S30. DSC plots of **P11**-*b*-**P5**$_{cis}$-*b*-**P11** (99% *cis*, left) and **P11**-*b*-**P5**$_{trans}$-*b*-**P11** (89% *trans*, right).

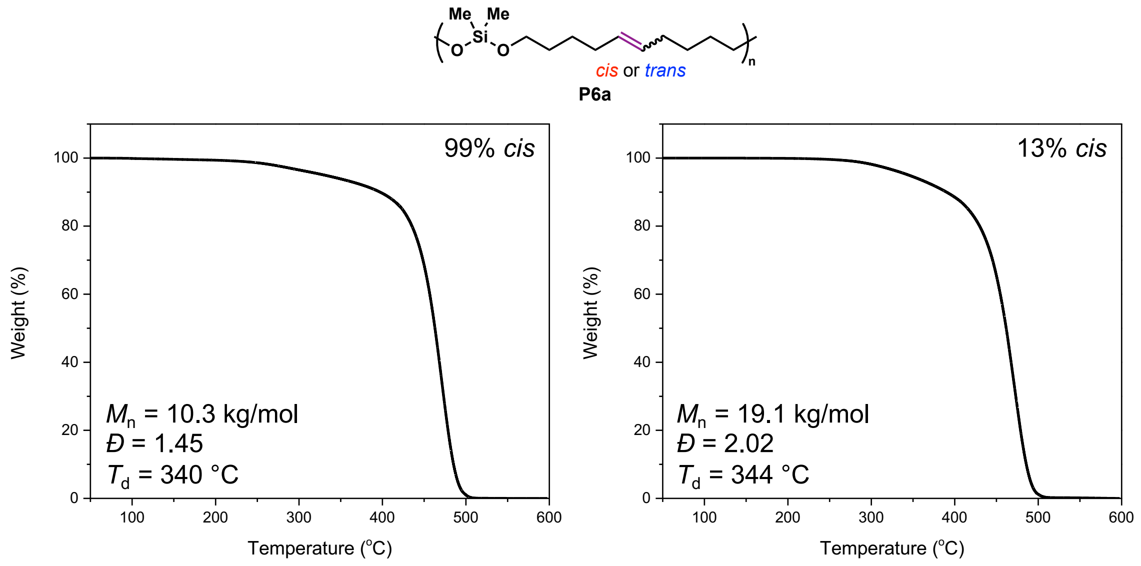

Figure S31. TGA thermograms of 99% *cis* **P6a** (left) and 13% *cis* **P6a** (right).

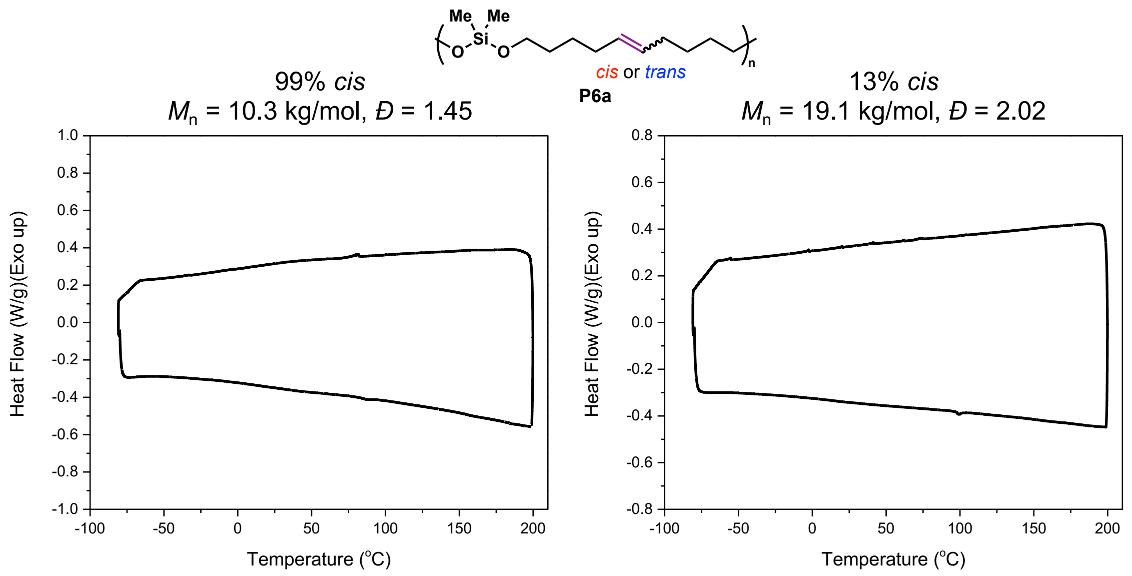

Figure S32. DSC plots of 99% *cis* **P6a** (left) and 13% *cis* **P6a** (right).

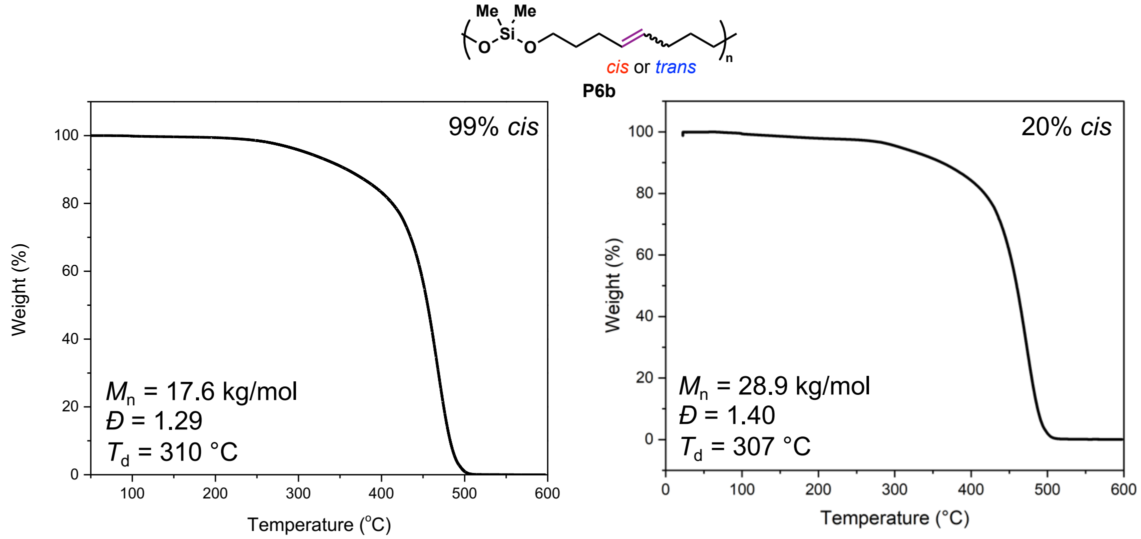

Figure S33. TGA thermograms of 99% *cis* **P6b** (left) and 20% *cis* **P6b** (right).

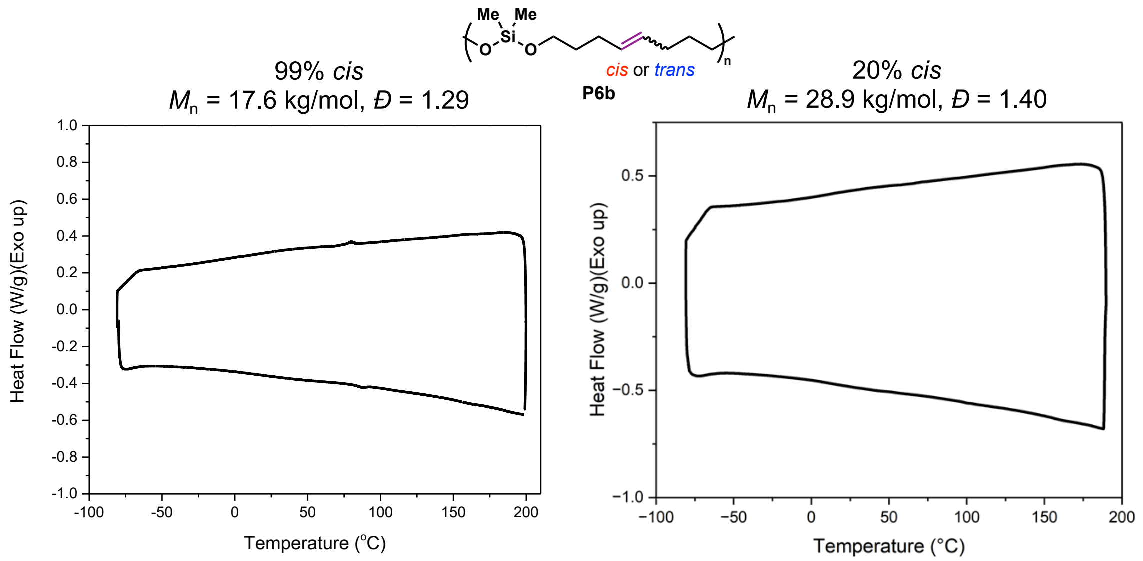

Figure S34. DSC plots of 99% *cis* **P6b** (left) and 20% *cis* **P6b** (right).

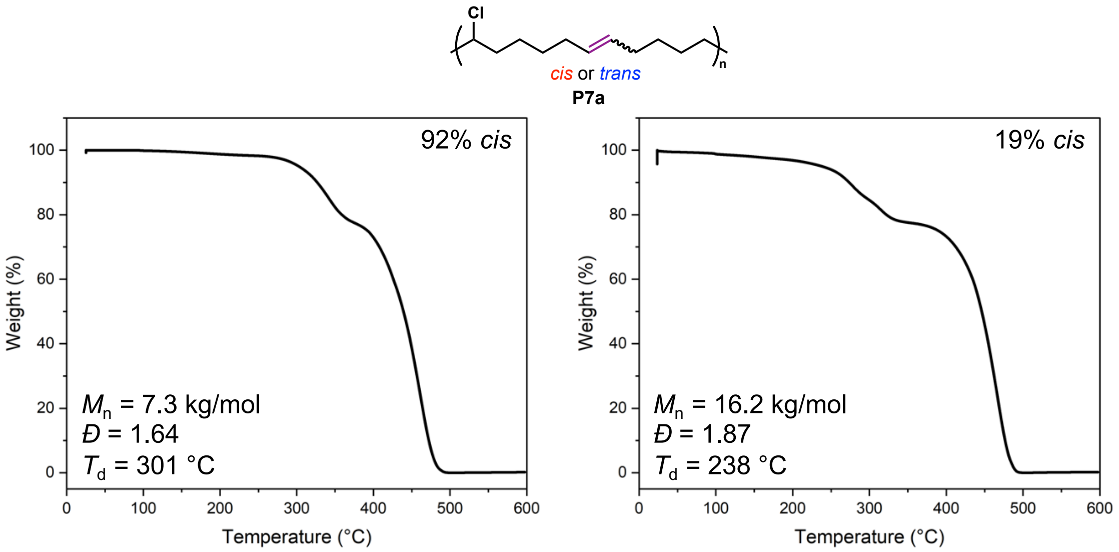

Figure S35. TGA thermograms of **P7a** with 92% (left) and 19% (right) *cis* content.

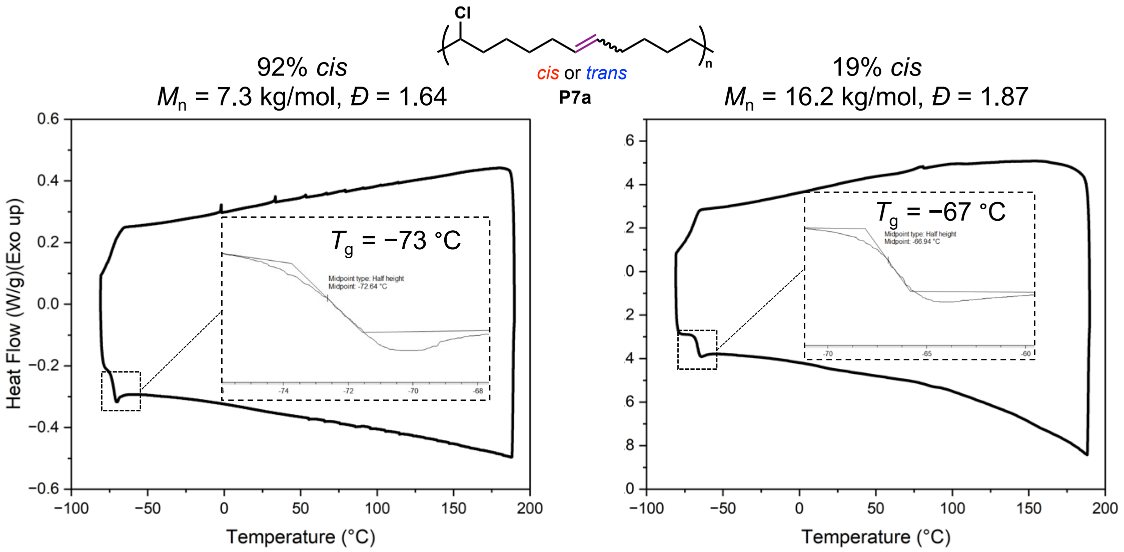

Figure S36. DSC plots of 99% *cis* **P7a** (left) and 19% *cis* **P7b** (right).

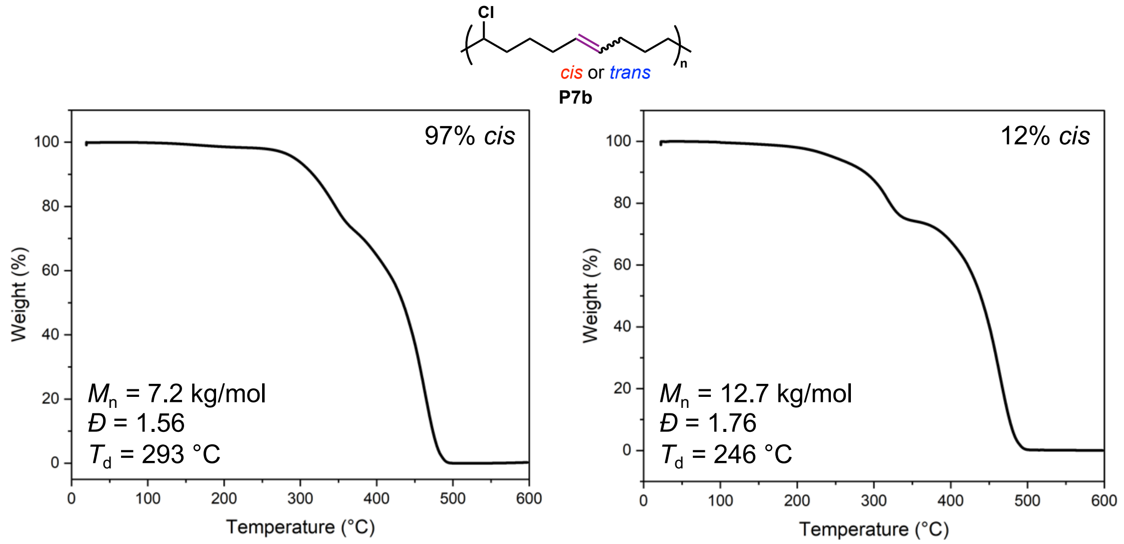

Figure S37. TGA thermograms of **P7b** with 97% (left) and 12% (right) *cis* content.

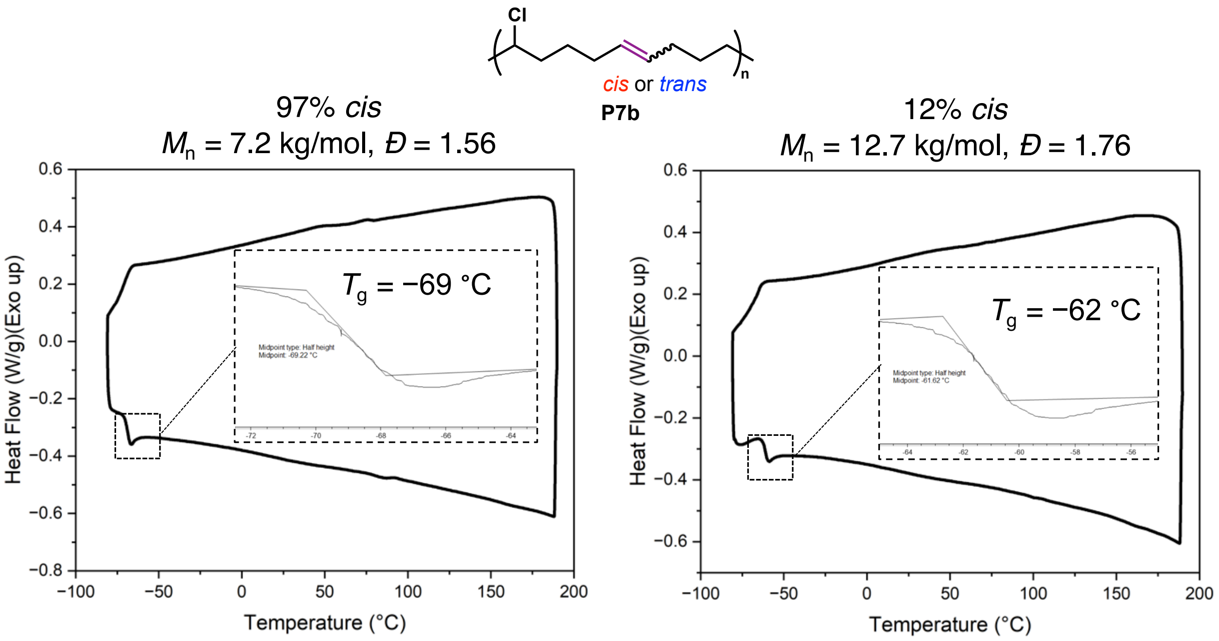

Figure S38. DSC plots of 97% *cis* **P7b** (left) and 12% *cis* **P7b** (right).

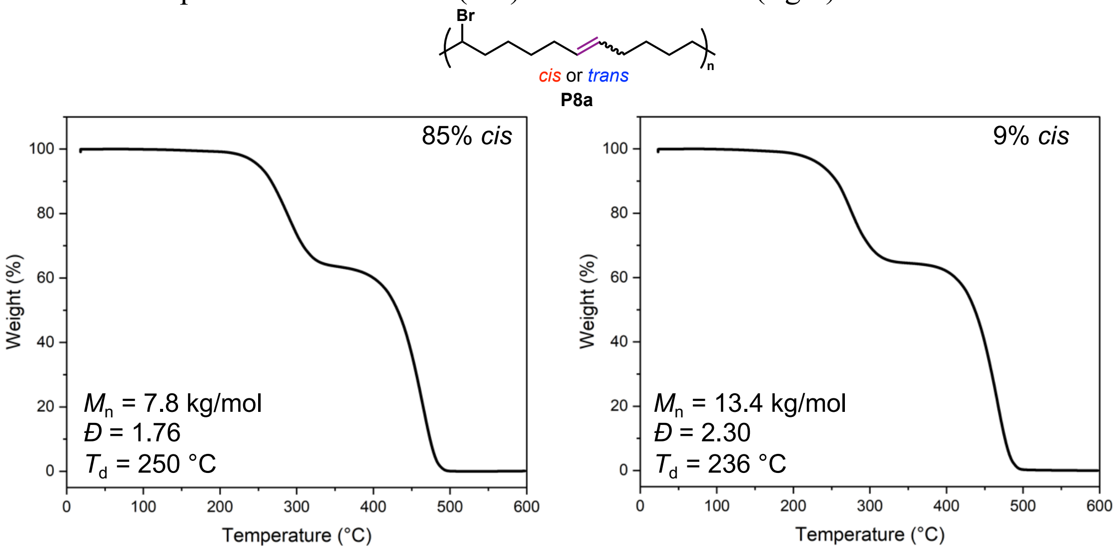

Figure S39. TGA thermograms of **P8a** with 85% (left) and 9% (right) *cis* content.

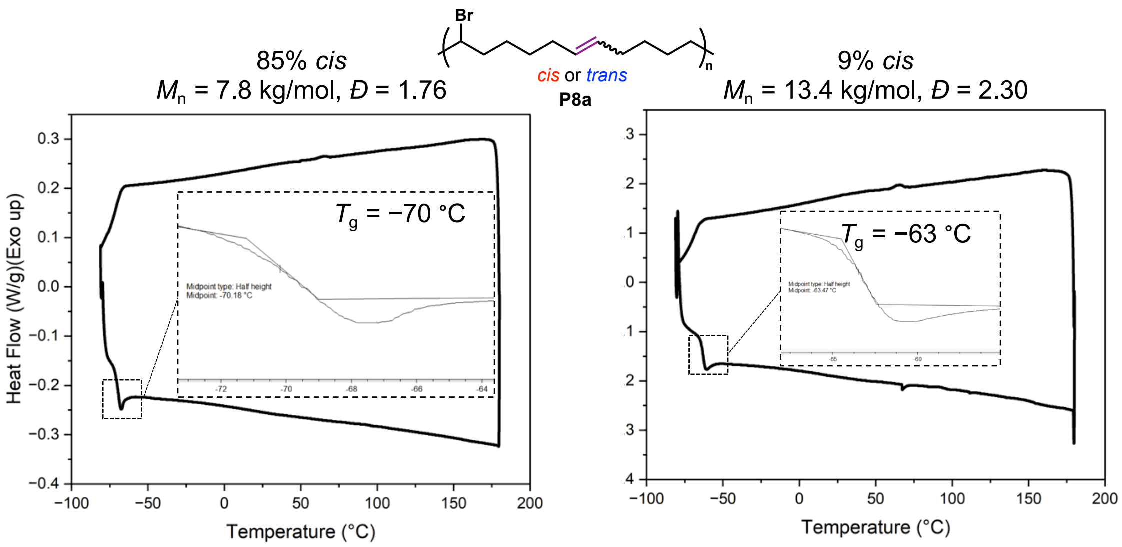

Figure S40. DSC plots of 85% *cis* **P8a** (left) and 9% *cis* **P8a** (right).

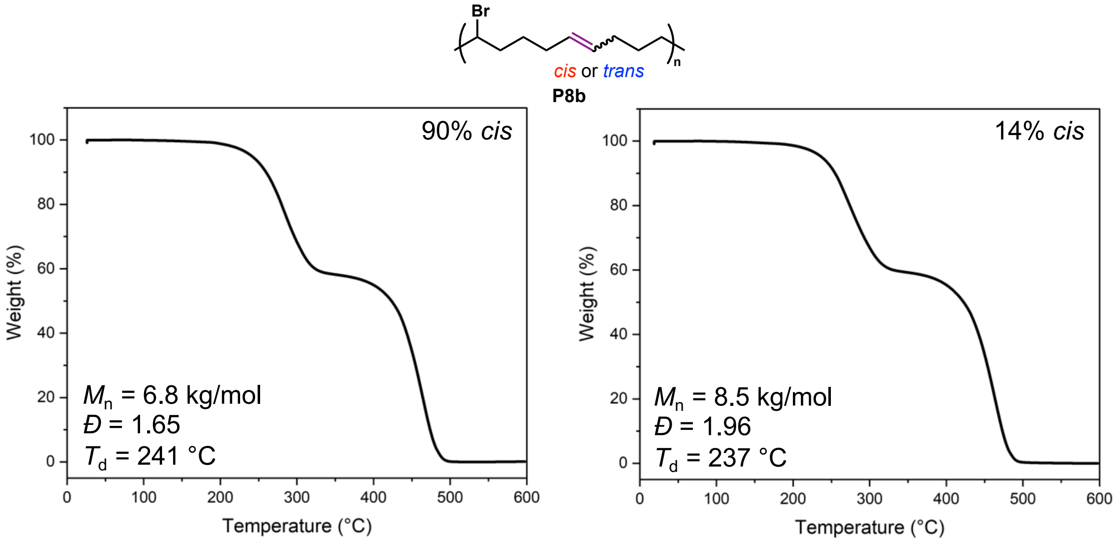

Figure S41. TGA thermograms of **P8b** with 90% (left) and 14% (right) *cis* content.

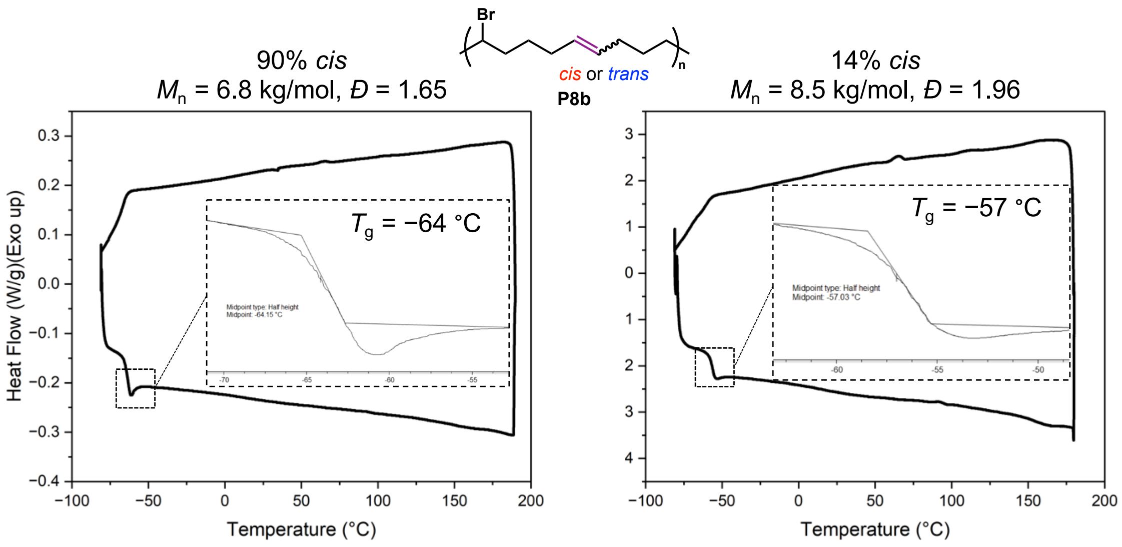

Figure S42. DSC thermograms of **P8b** with 90% and 14% *cis* content.

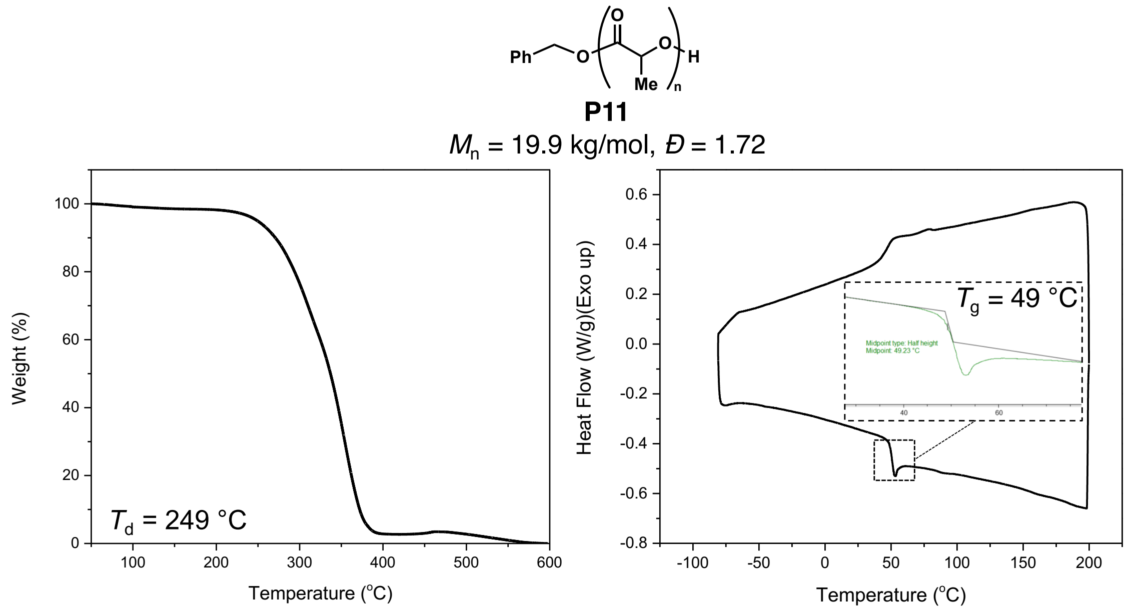

Figure S43. TGA (left) and DSC (right) thermograms of **P11**.

Nanoindentation Data

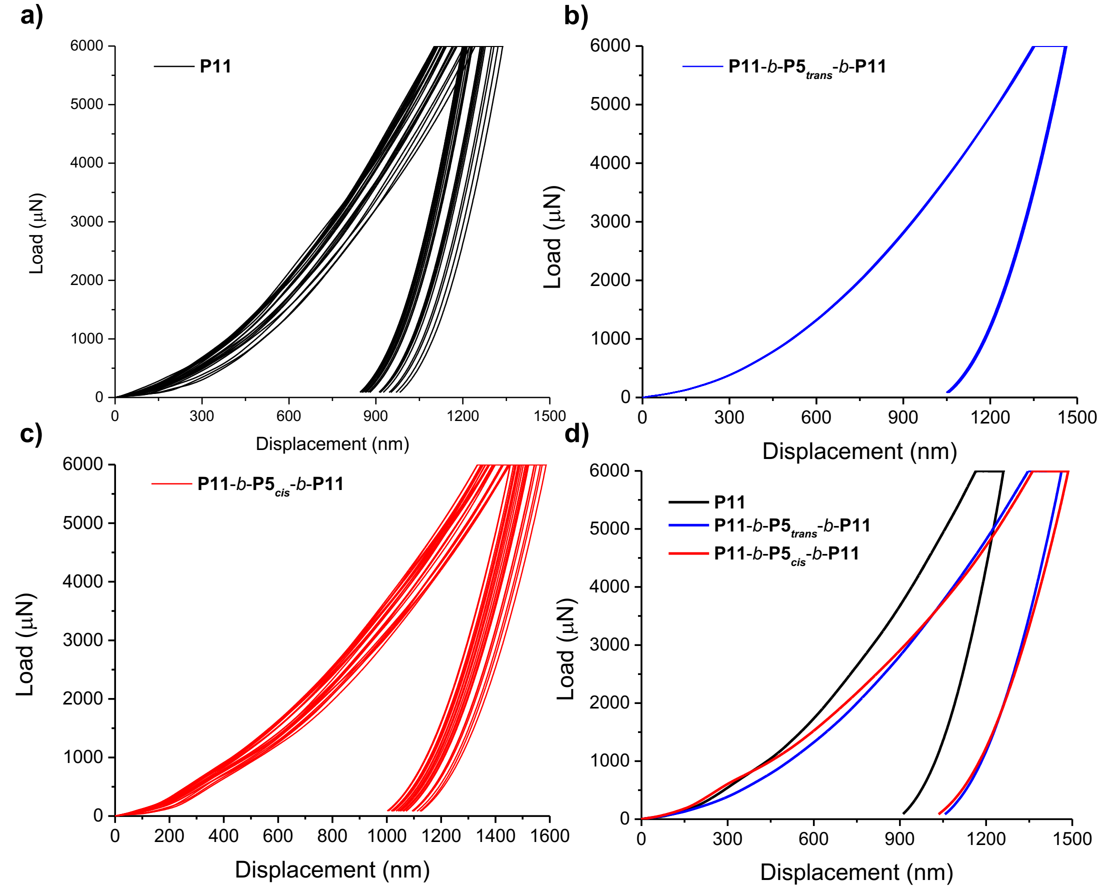

Figure S44. Load-displacement curves from 25 indents of **a) P11**, **b) P11**-*b*-**P5**$_{trans}$-*b*-**P11**, and **c) P11**-*b*-**P5**$_{cis}$-*b*-**P11** and **d)** representative comparison of load-displacement curves of polymers from one indent.

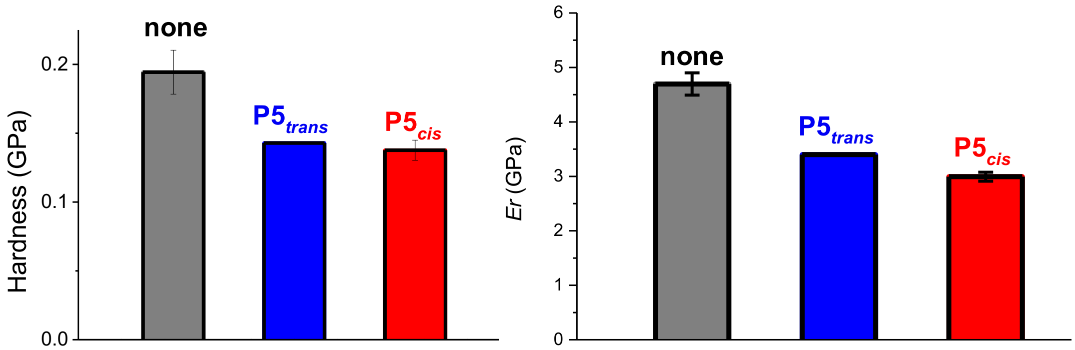

Figure S45. (Left) Bar graph representing hardness of **P11** (gray, 0.19 ± 0.02 GPA), **P11**-*b*-**P5**$_{trans}$-*b*-**P11** (blue, 0.1429 ± 0.0004 GPA), and **P11**-*b*-**P5**$_{cis}$-*b*-**P11**(red, 0.138 ± 0.007 GPA) and (right) bar graph representing E_r of **P11** (gray, 4.7 ± 0.2 GPA), **P5**$_{trans}$-*b*-**P11** (blue, 3.400 ± 0.007 GPA), and **P11**-*b*-**P5**$_{cis}$-*b*-**P11**(red, 2.99 ± 0.08 GPA).

NMR Spectra

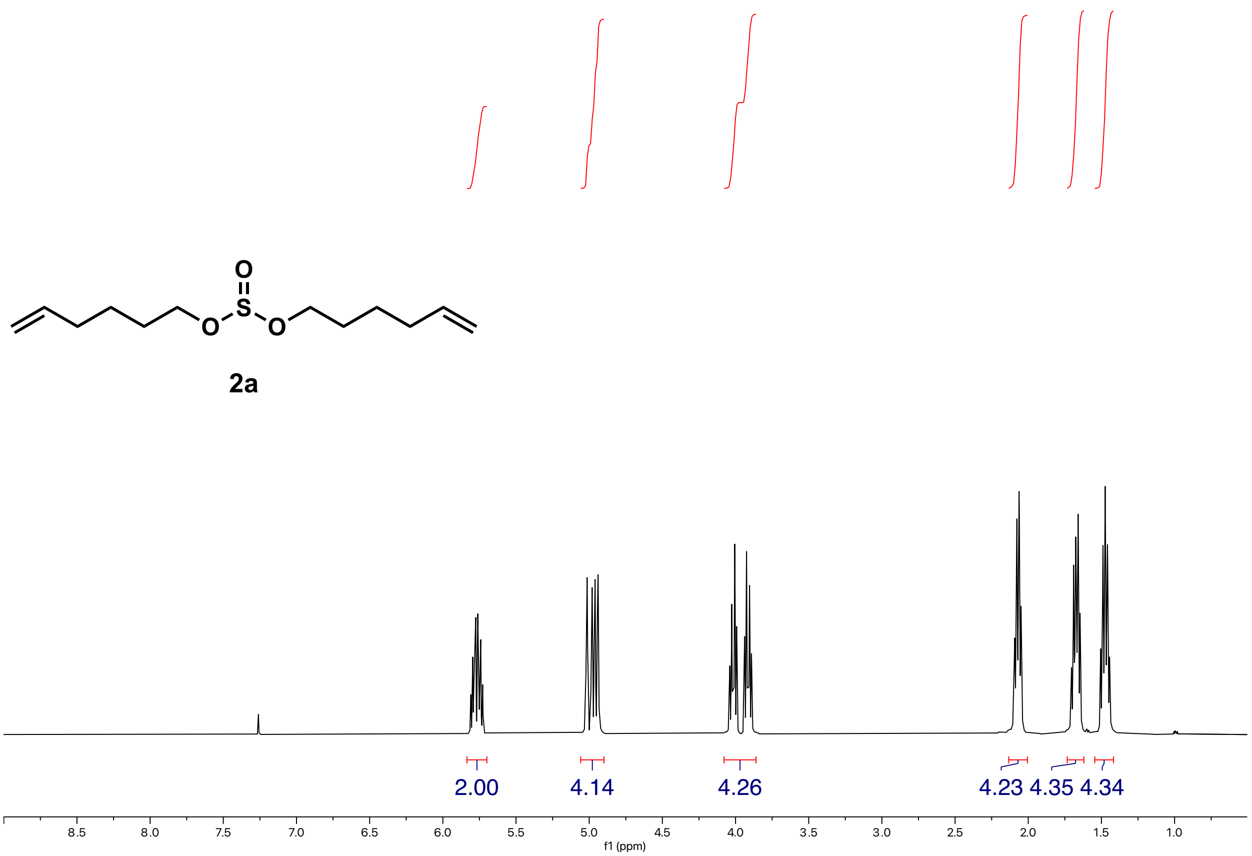

Figure S46. ^{1}H NMR (500 MHz, $CDCl_3$) spectrum of compound **2a**.

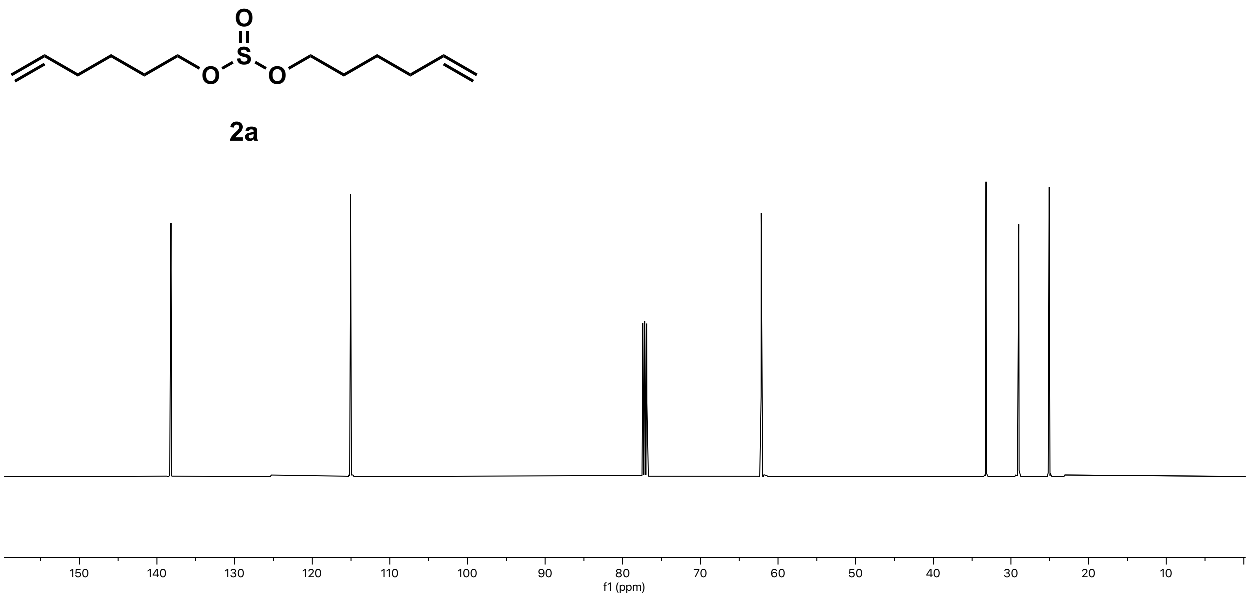

Figure S47. ^{13}C NMR (125 MHz, $CDCl_3$) spectrum of compound **2a**.

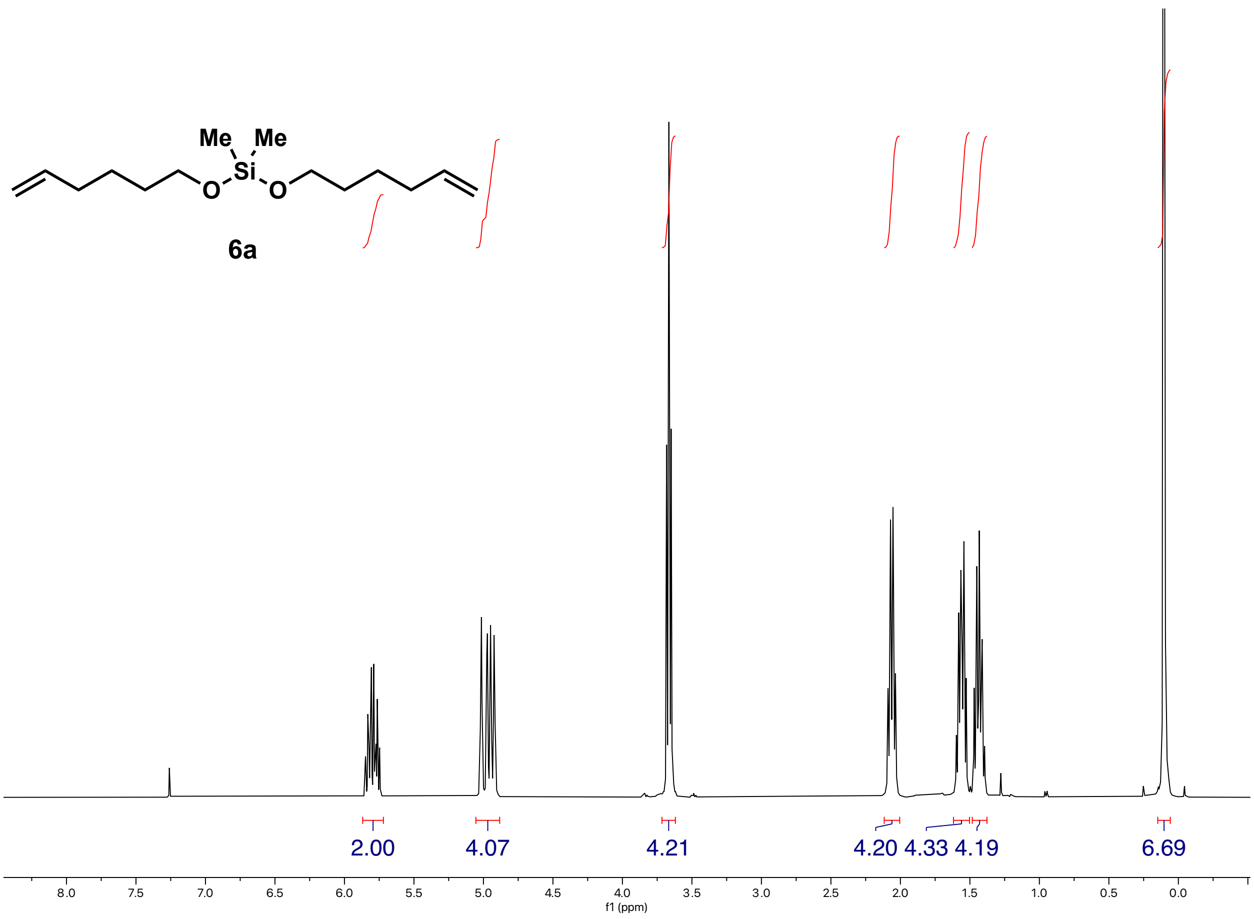

Figure S48. ^{1}H NMR (400 MHz, $CDCl_3$) spectrum of compound **6a**.

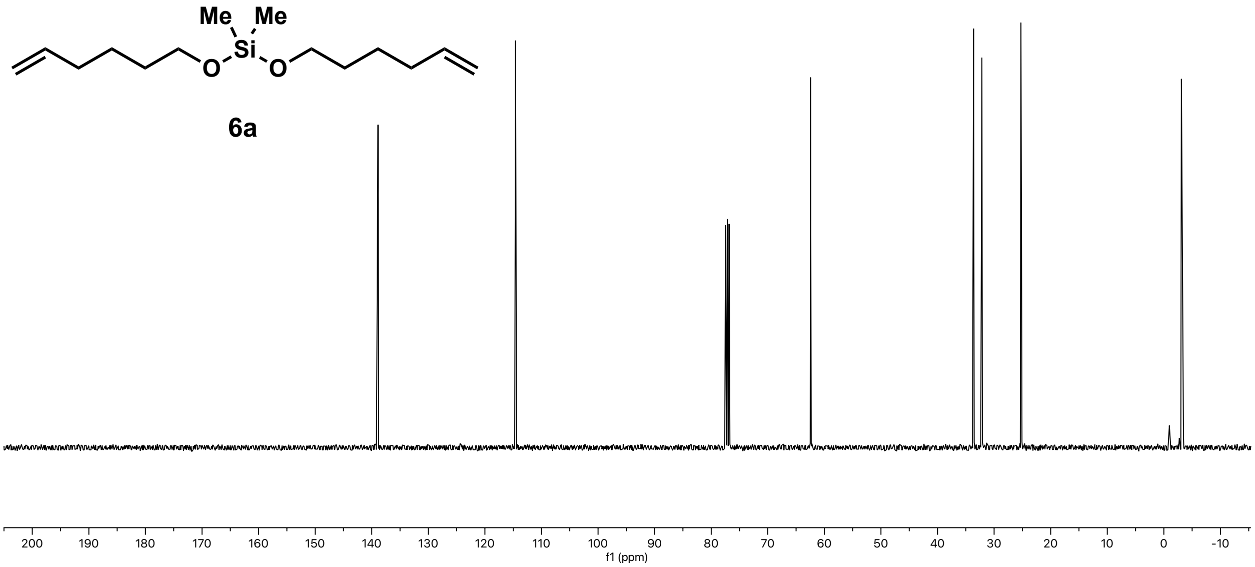

Figure S49. ^{13}C NMR (101 MHz, $CDCl_3$) spectrum of compound **6a**.

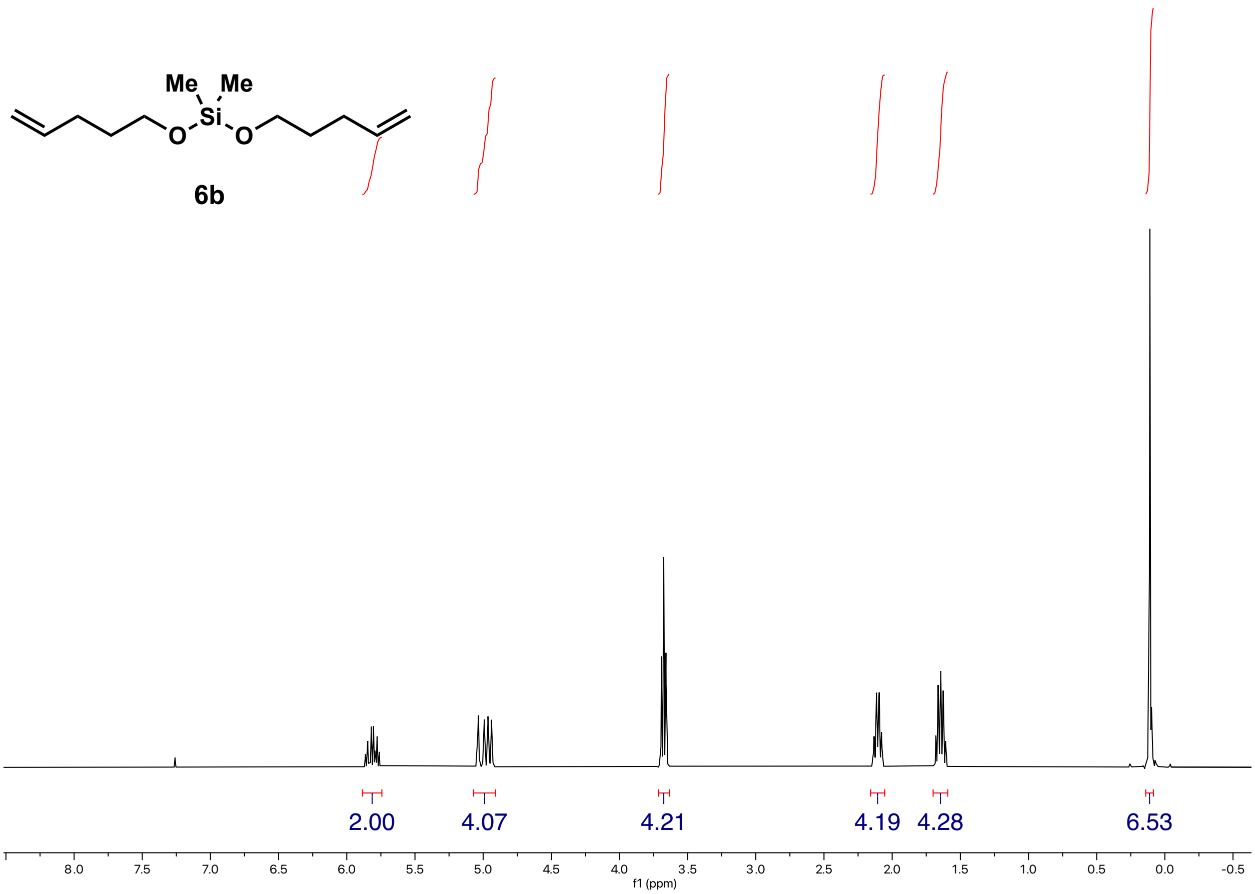

Figure S50. ^{1}H NMR (400 MHz, $CDCl_3$) spectrum of compound **6b**.

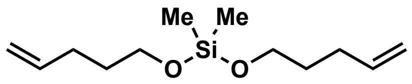

6b

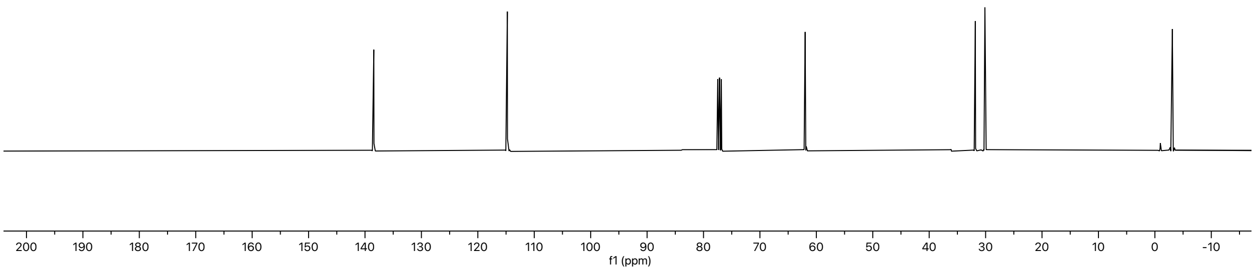

Figure S51. ^{13}C NMR (101 MHz, $CDCl_3$) spectrum of compound **6b**.

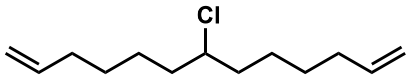

7a

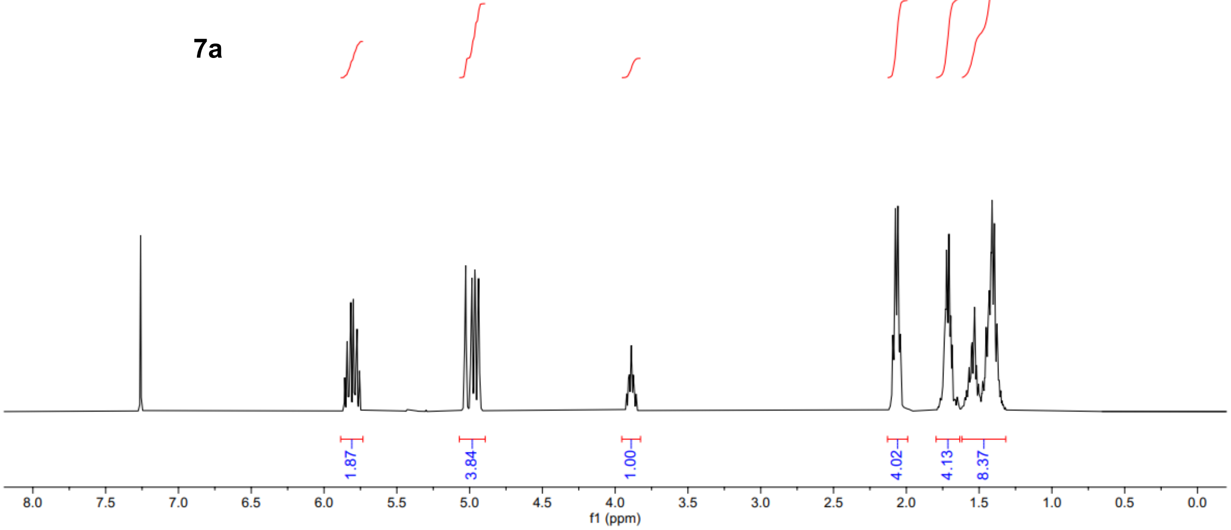

Figure S52. ^{1}H NMR (400 MHz, $CDCl_3$) spectrum of compound **7a**.

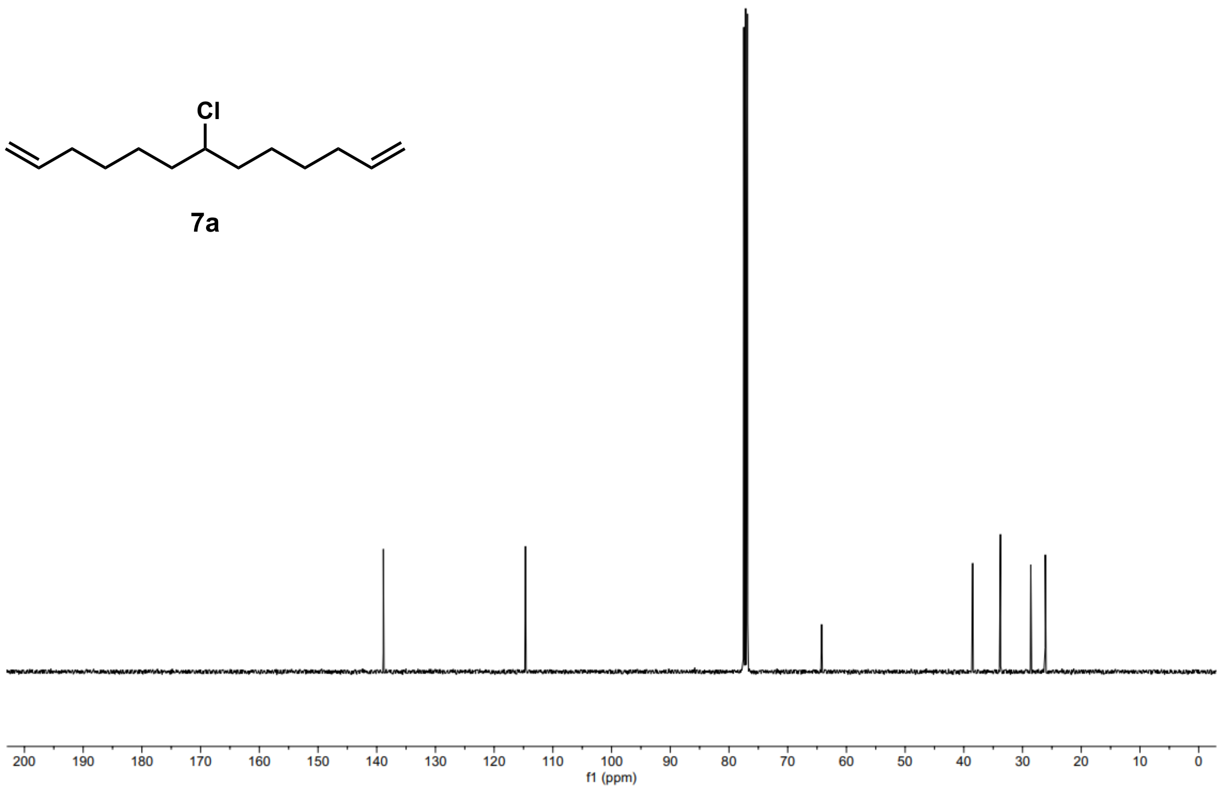

Figure S53. ^{13}C NMR (101 MHz, $CDCl_3$) spectrum of compound **7a**.

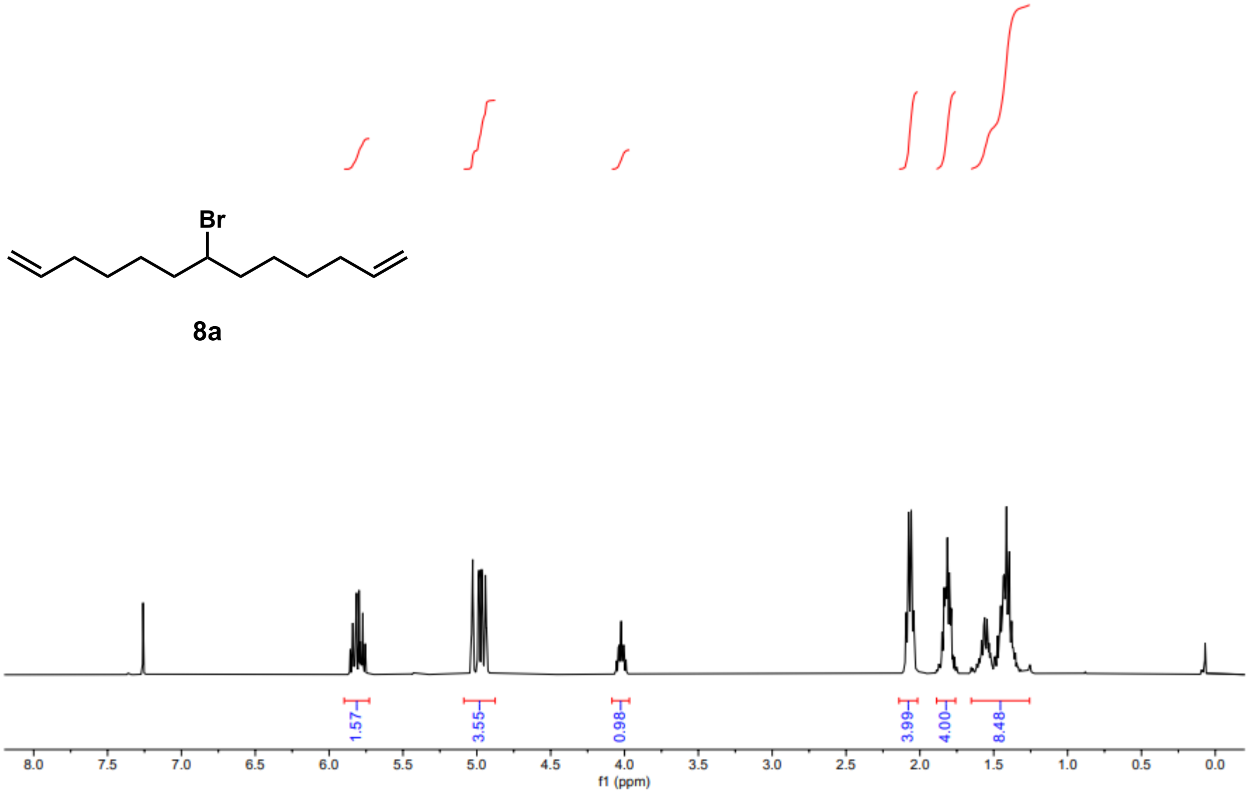

Figure S54. ^{1}H NMR (400 MHz, $CDCl_3$) spectrum of compound **8a**.

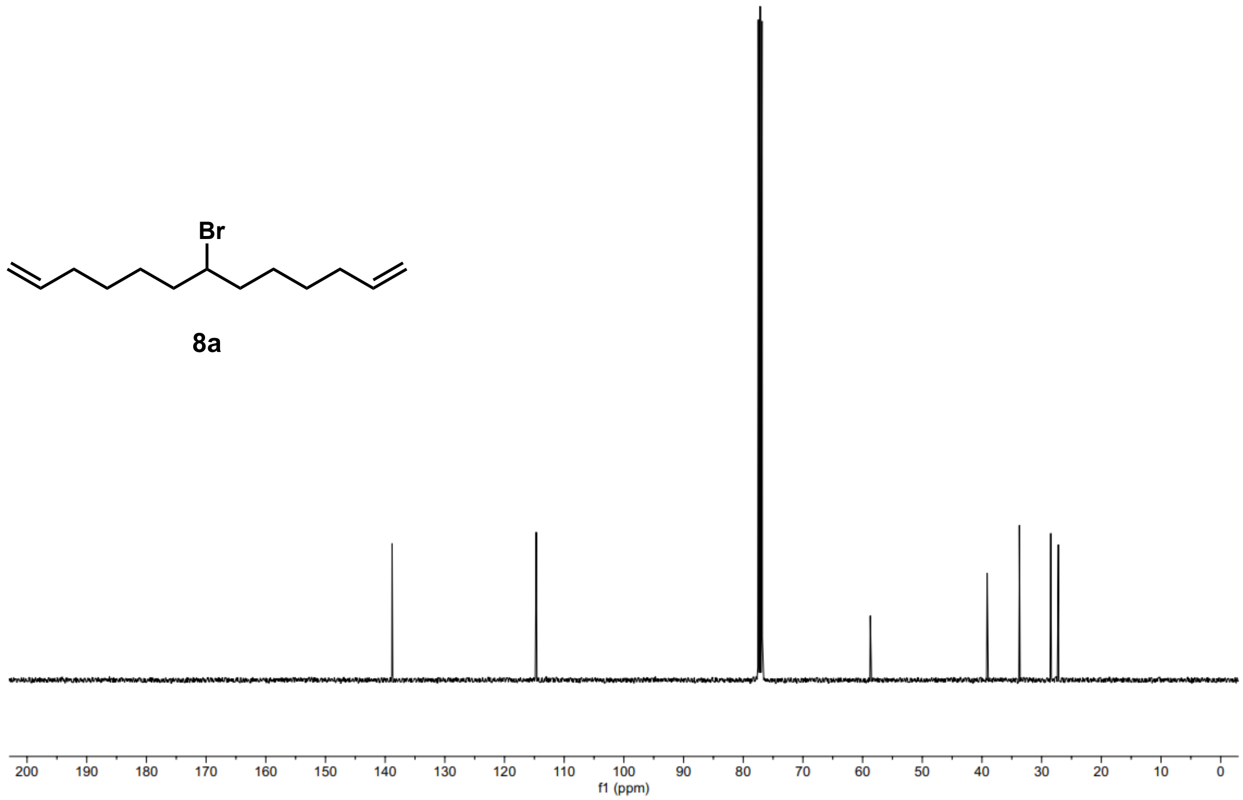

Figure S55. ^{13}C NMR (101 MHz, $CDCl_3$) spectrum of compound **8a**.

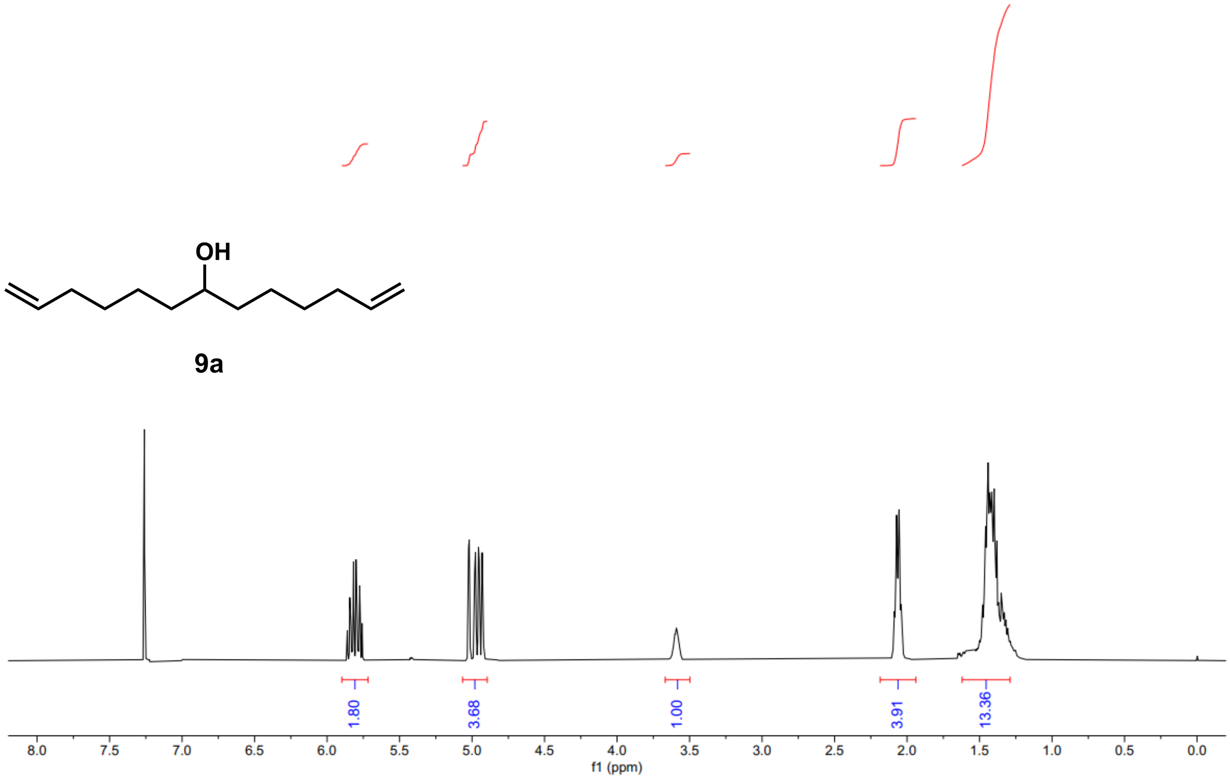

Figure S56 ^{1}H NMR (400 MHz, $CDCl_3$) spectrum of compound **9a**.

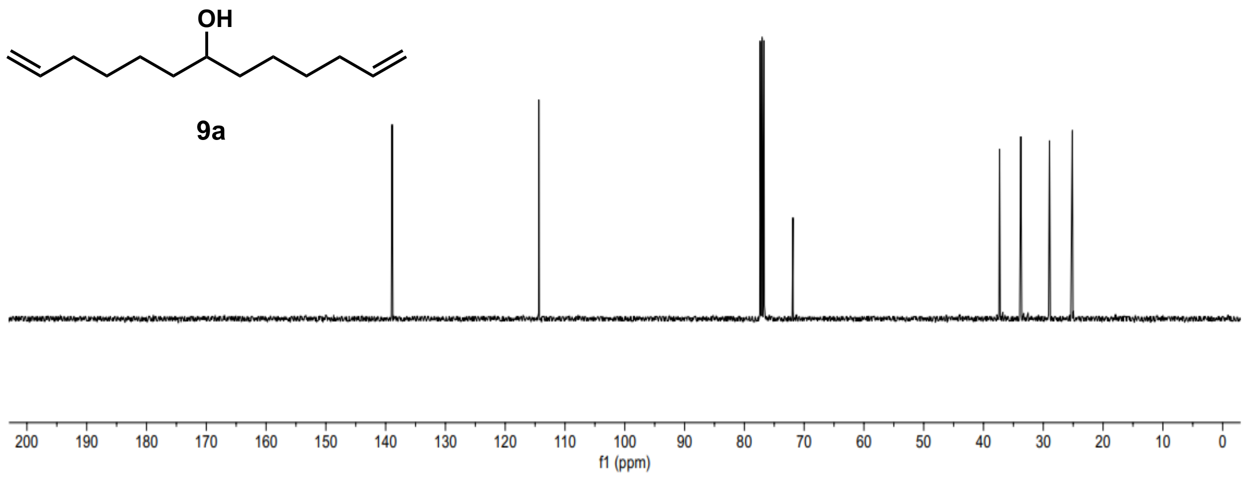

Figure S57. ^{13}C NMR (101 MHz, $CDCl_3$) spectrum of compound **9a**.

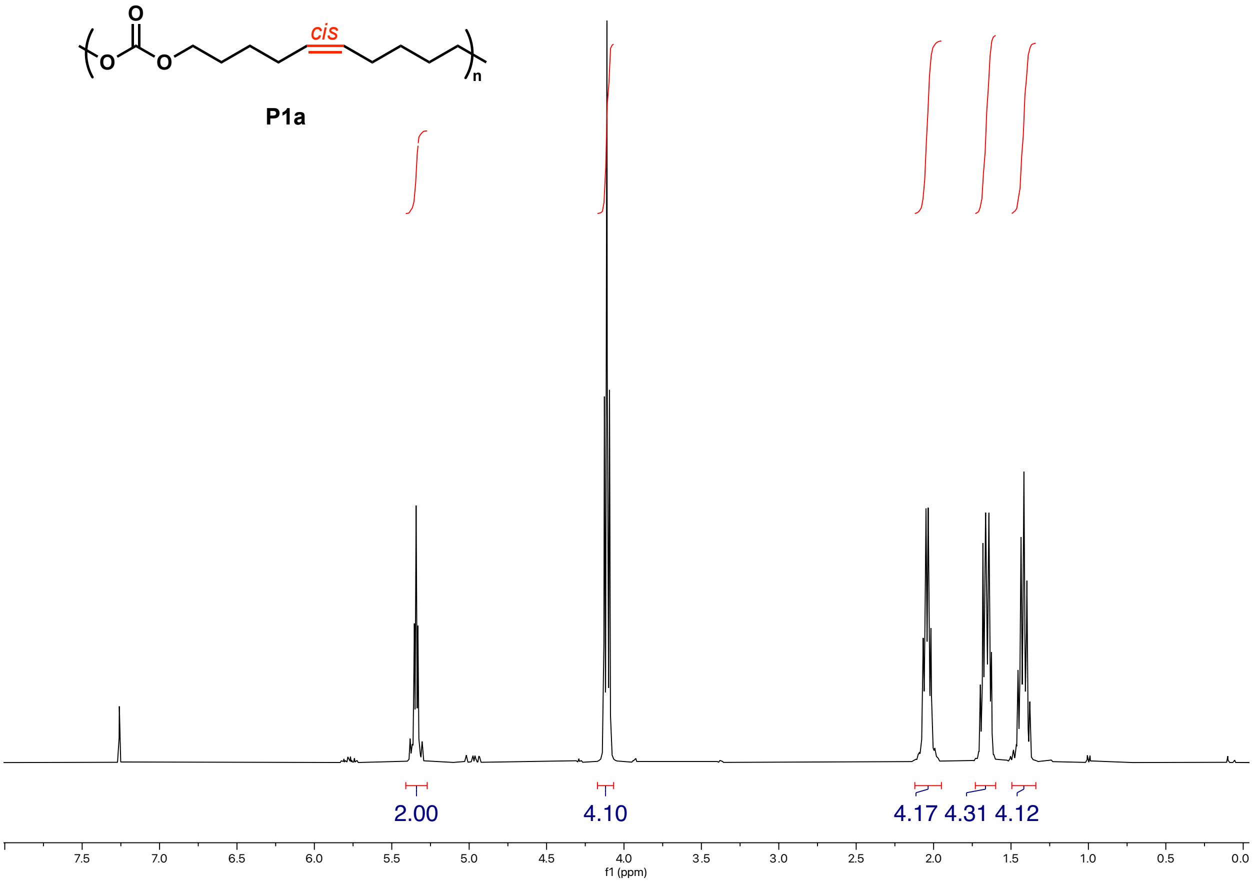

Figure S58. ^{1}H NMR (400 MHz, $CDCl_3$) spectrum of **P1a**.

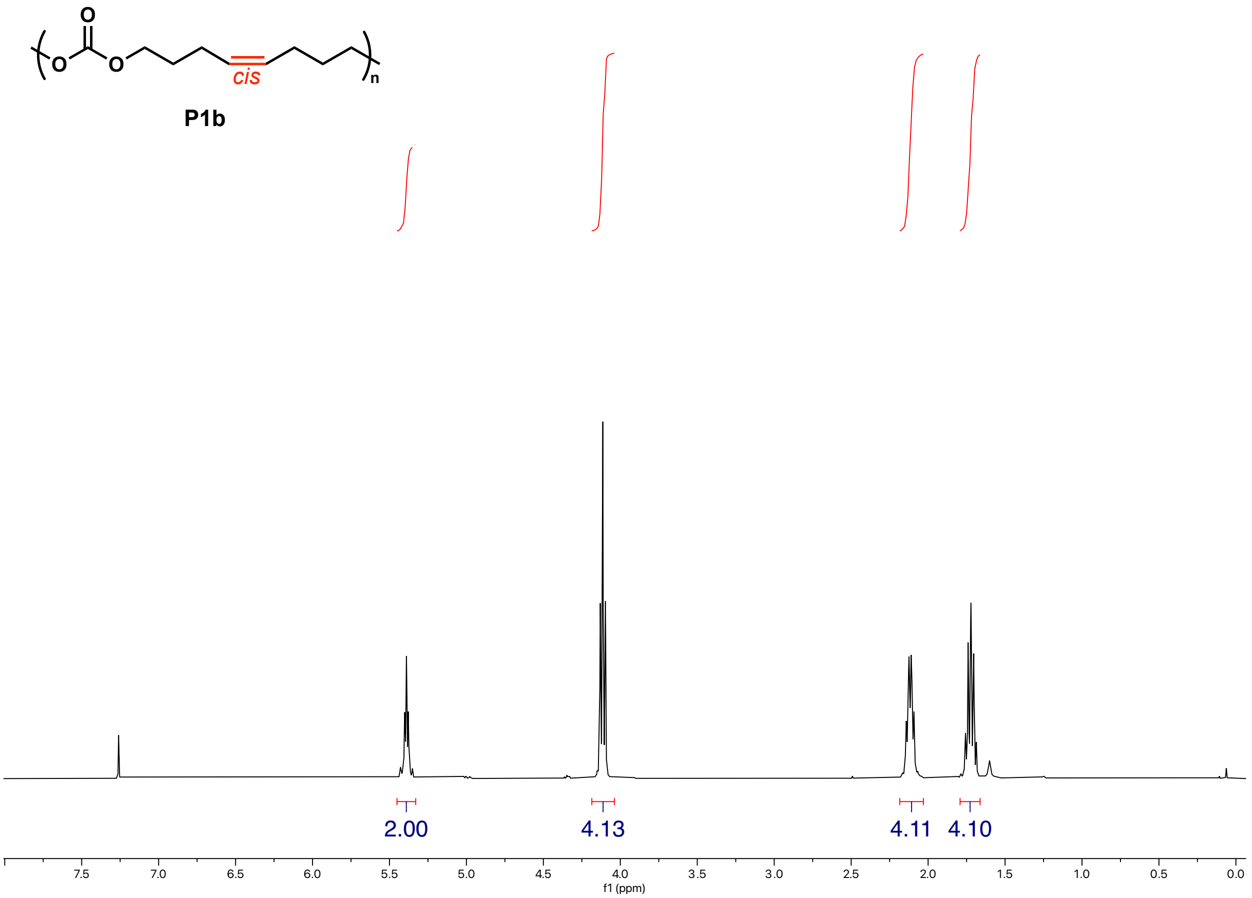

Figure S59. ^1H NMR (400 MHz, $CDCl_3$) spectrum of *cis*-rich **P1b**.

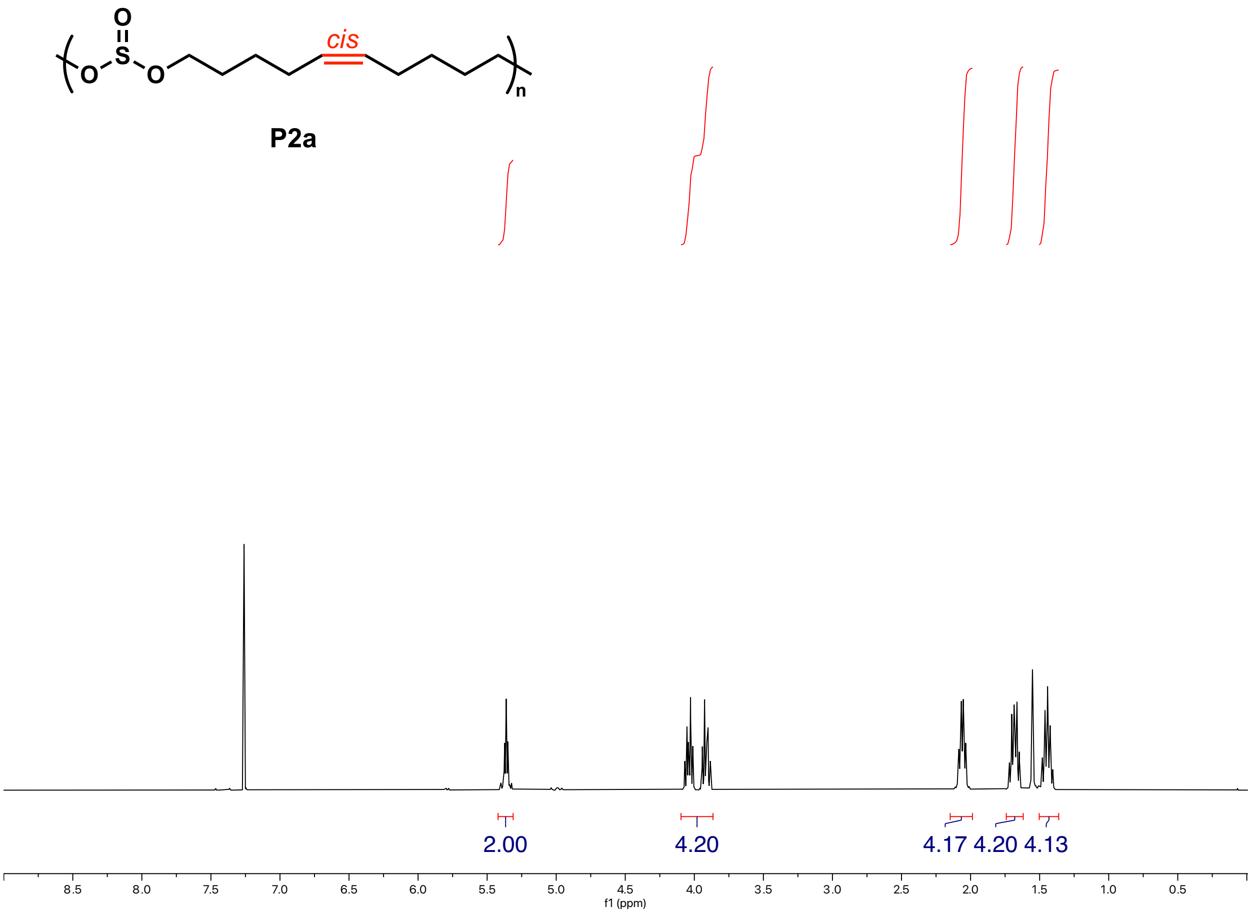

Figure S60. ^{1}H NMR (400 MHz, $CDCl_3$) spectrum of *cis*-rich **P2a**.

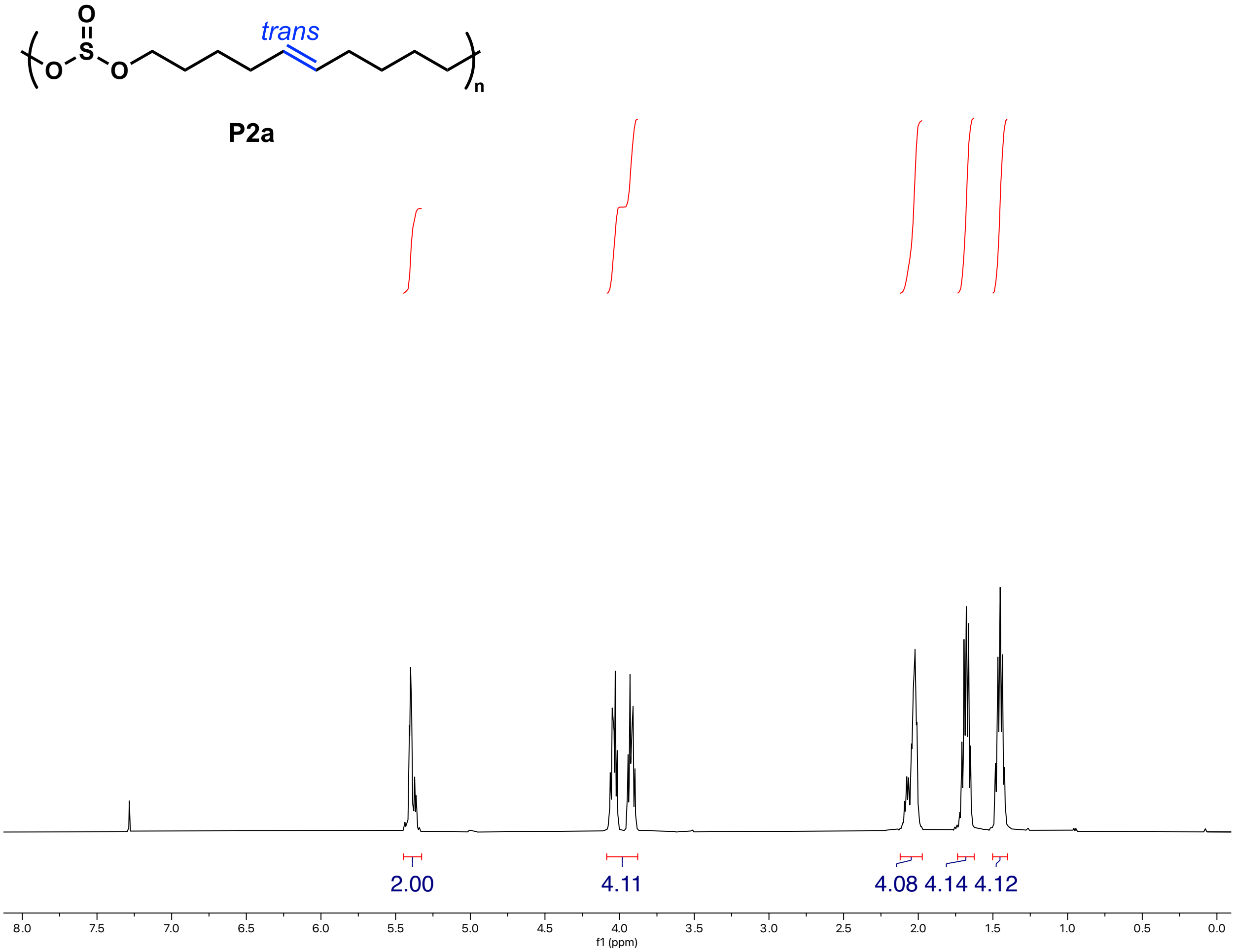

Figure S61. ^{1}H NMR (400 MHz, $CDCl_3$) spectrum of *trans*-rich **P2a**.

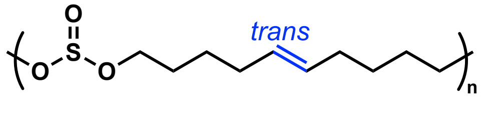

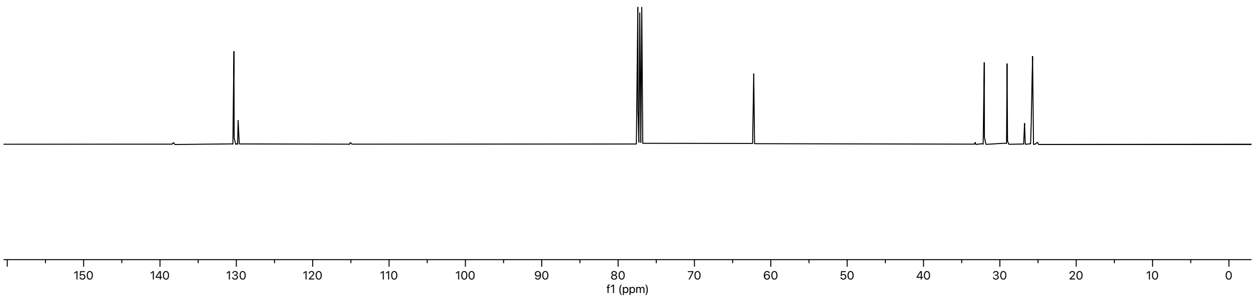

Figure S62. ^{13}C NMR (125 MHz, $CDCl_3$) spectrum of *trans*-rich **P2a**.

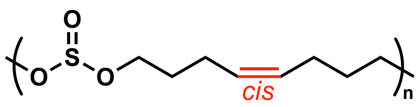

P2b

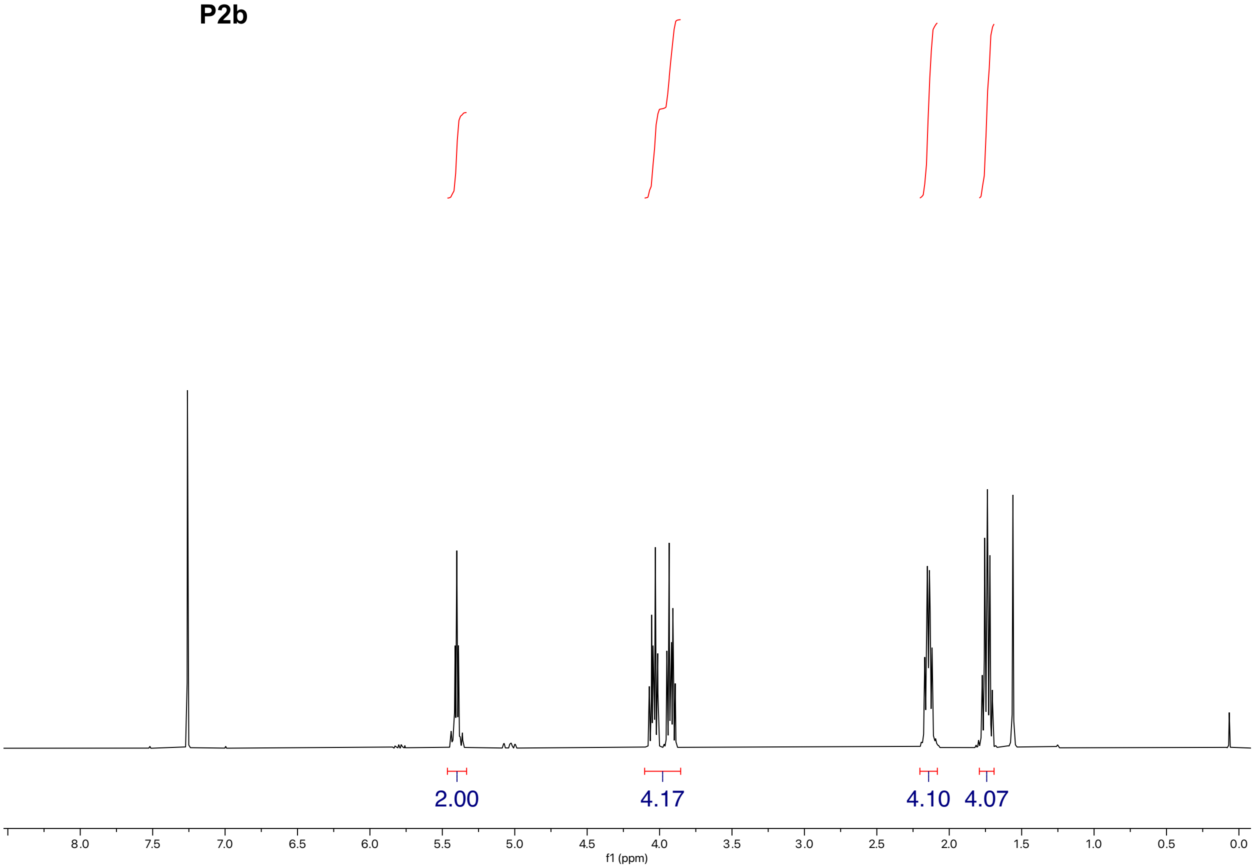

Figure S63. ^{1}H NMR (400 MHz, $CDCl_3$) spectrum of *cis*-rich **P2b**.

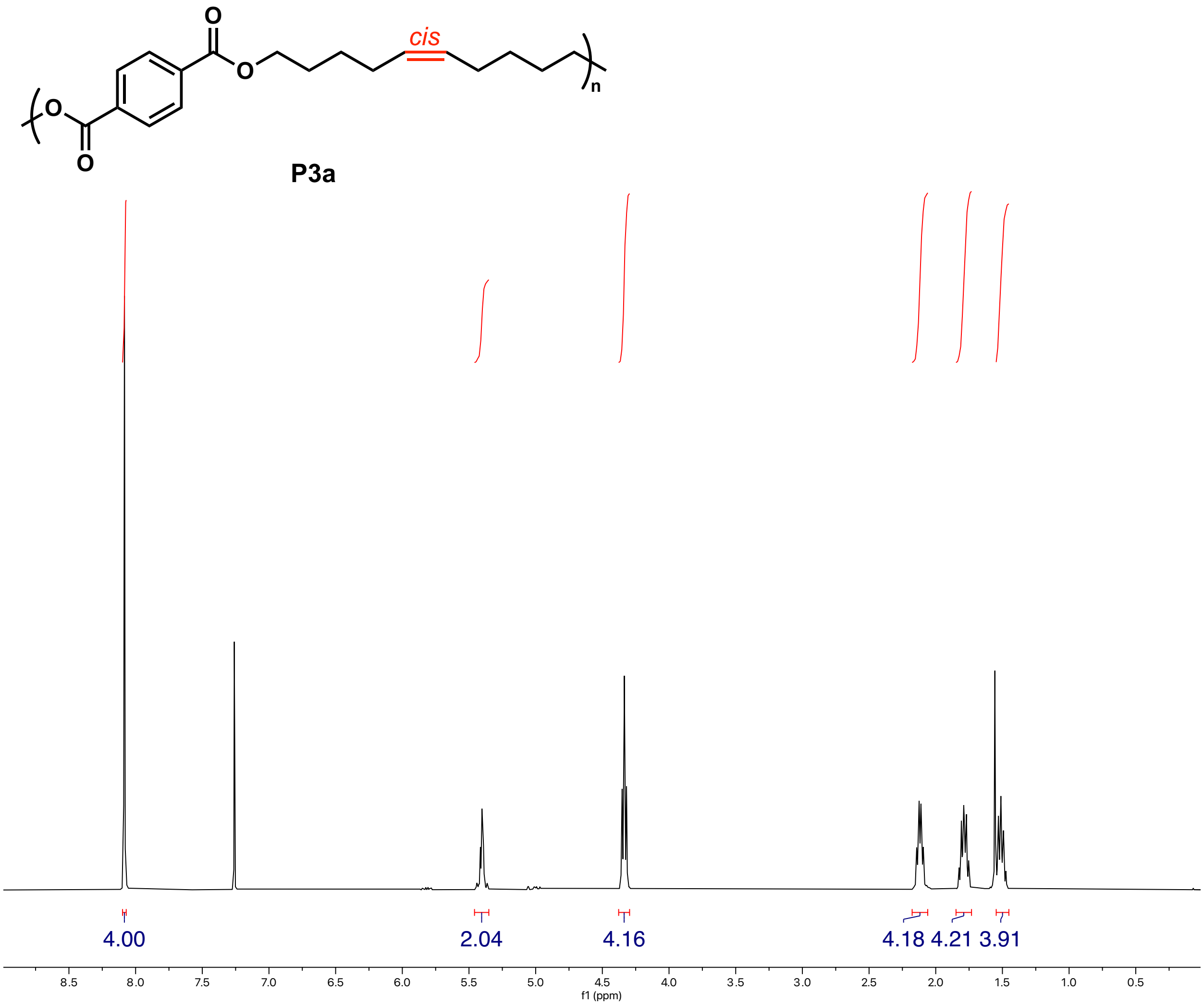

Figure S64. ^{1}H NMR (400 MHz, $CDCl_3$) spectrum of *cis*-rich **P3a**.

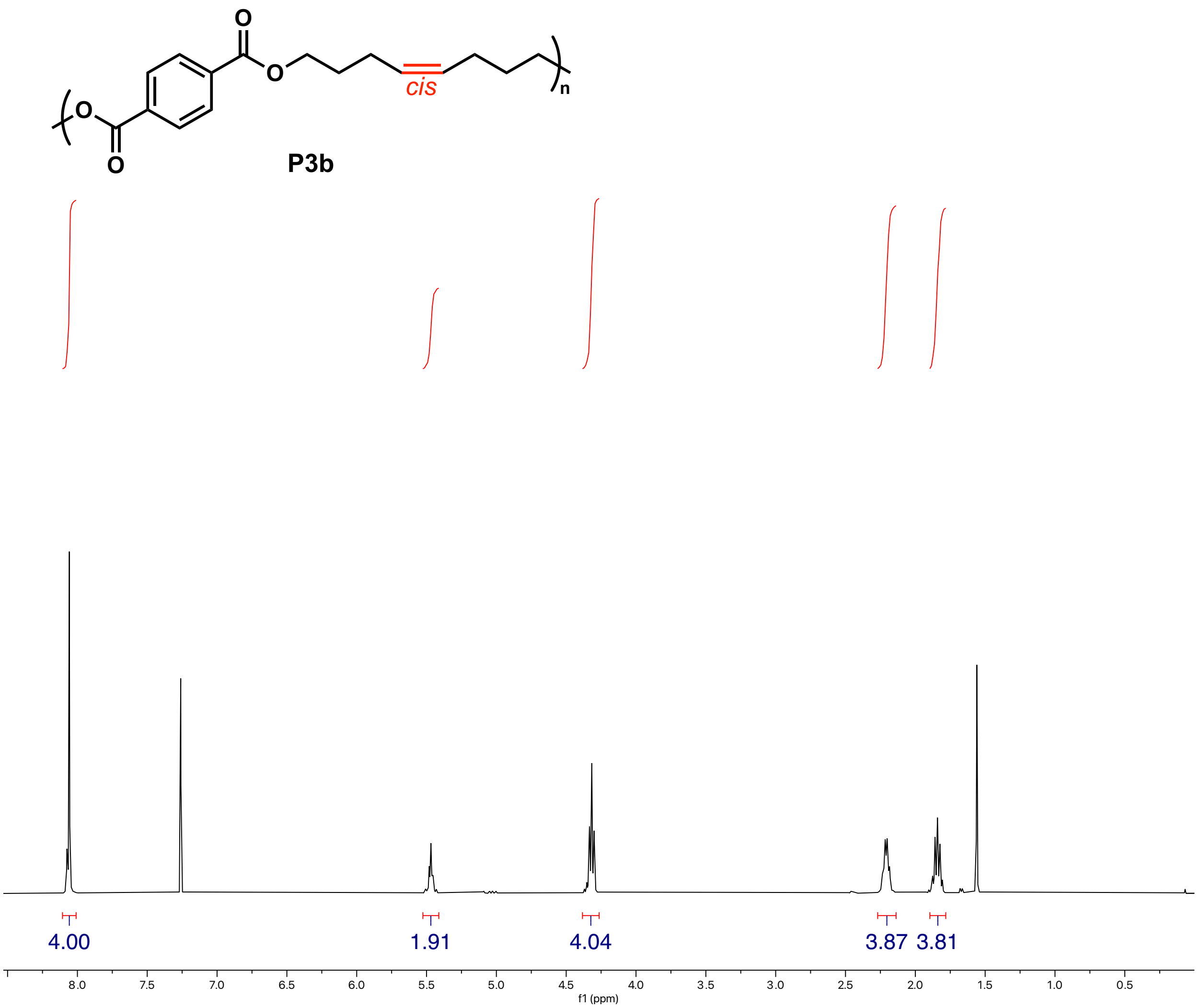

Figure S65. ^{1}H NMR (400 MHz, $CDCl_3$) spectrum of *cis*-rich **P3b**.

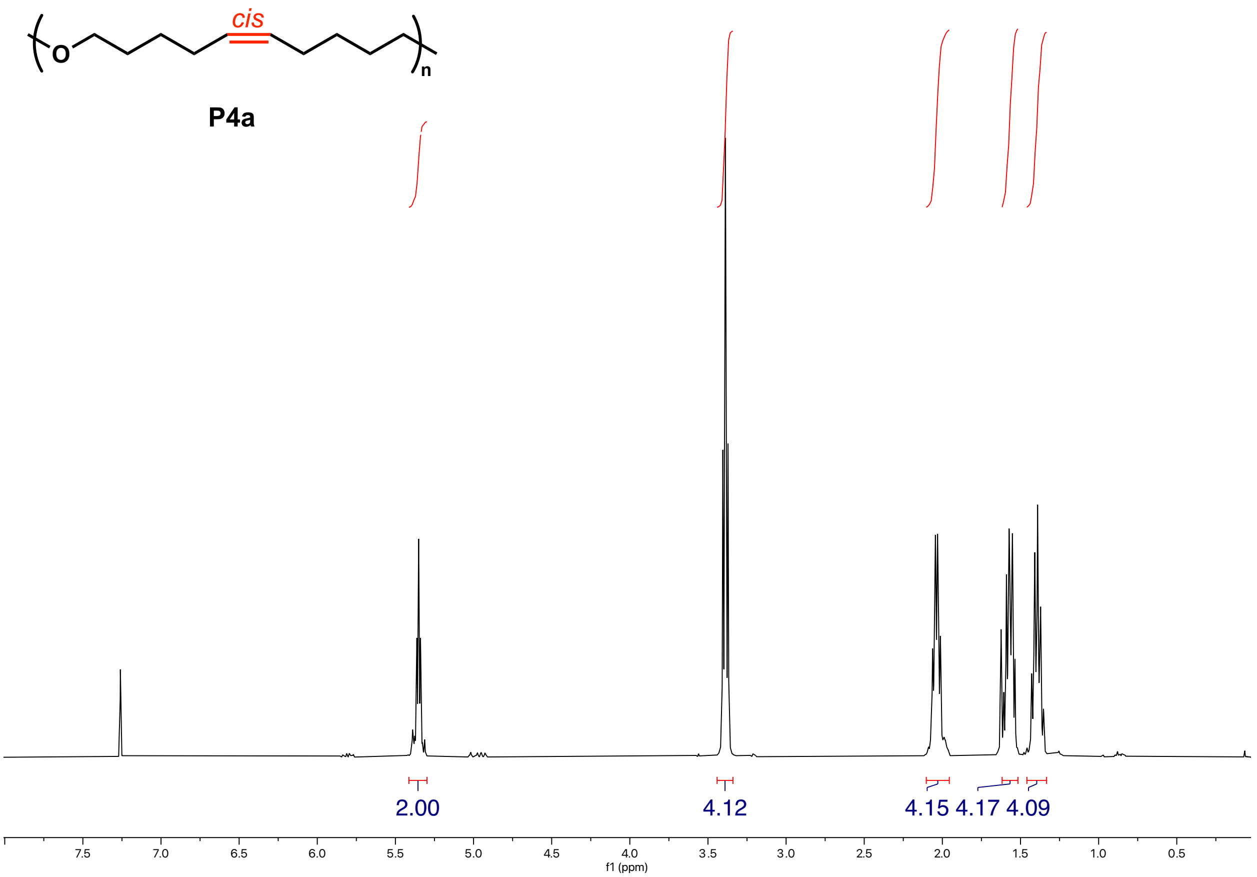

Figure S66. ^{1}H NMR (400 MHz, $CDCl_3$) spectrum of *cis*-rich **P4a**.

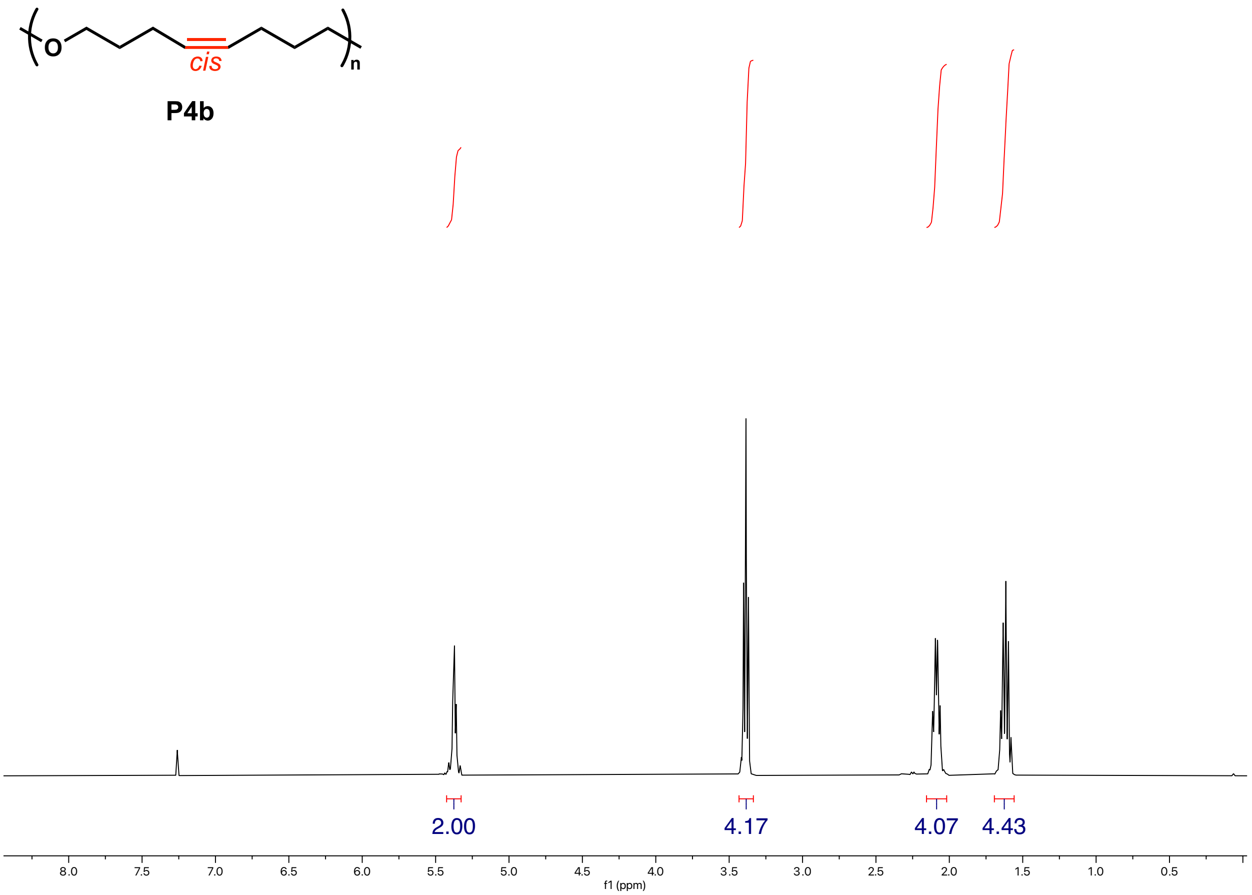

Figure S67. ^{1}H NMR (400 MHz, $CDCl_3$) spectrum of *cis*-rich **P4b**.

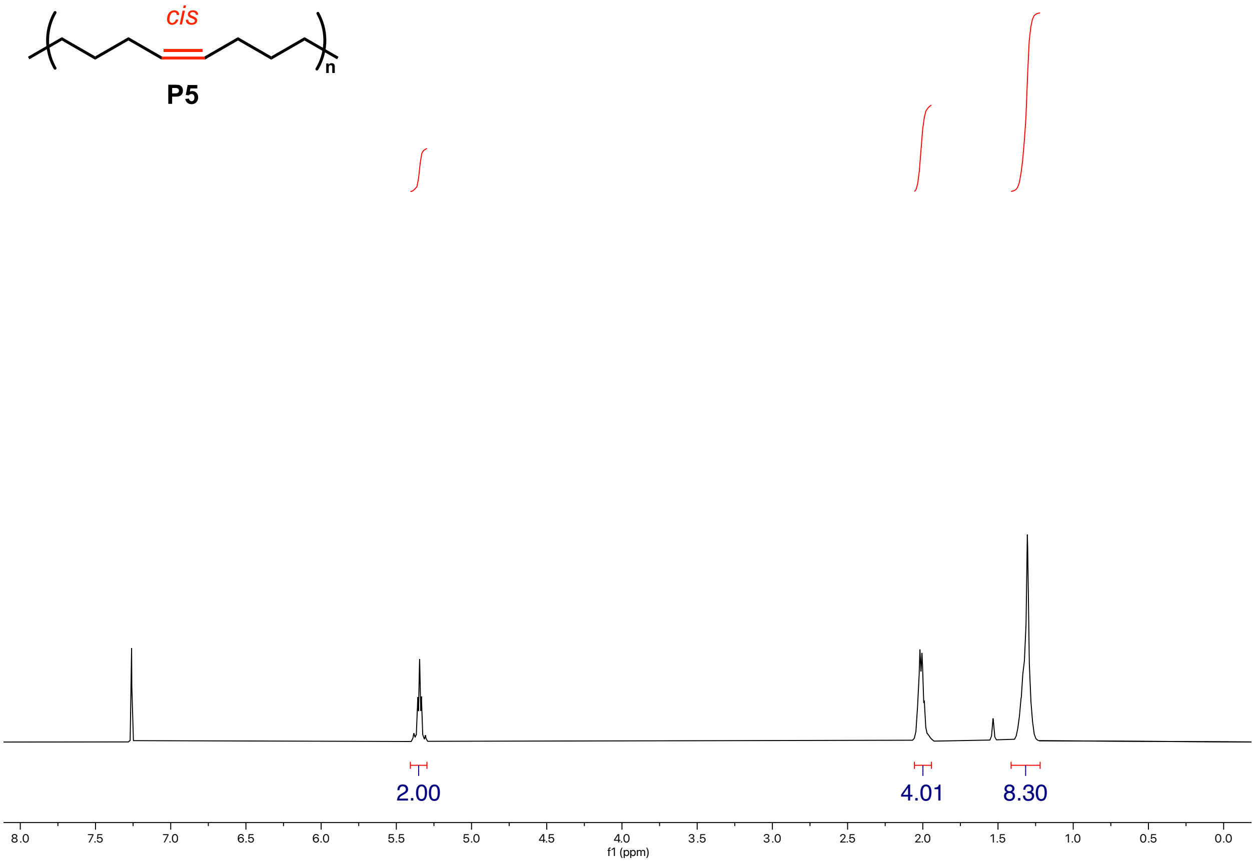

Figure S68. ^{1}H NMR (400 MHz, $CDCl_3$) spectrum of *cis*-rich compound **P5**.

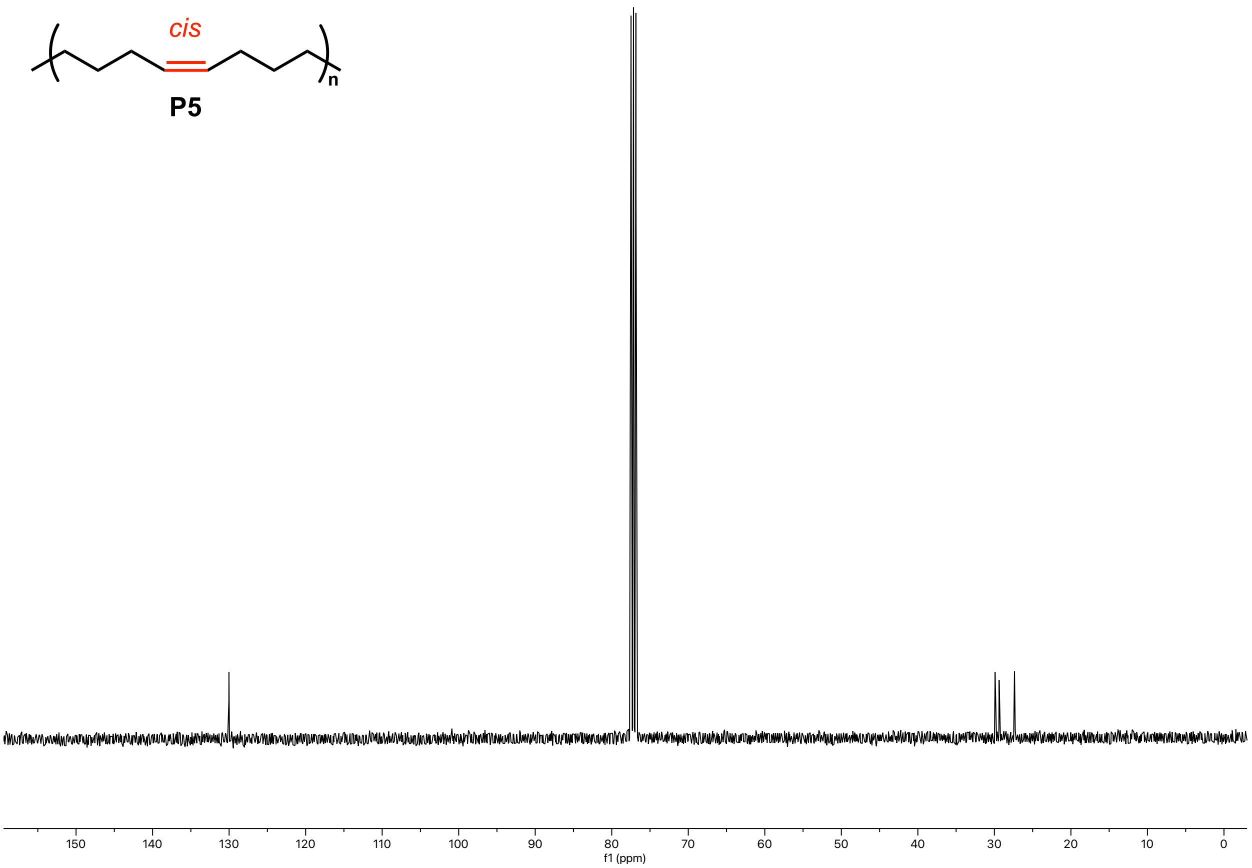

Figure S69. ^{13}C NMR (101 MHz, $CDCl_3$) spectrum of *cis*-rich compound **P5**.

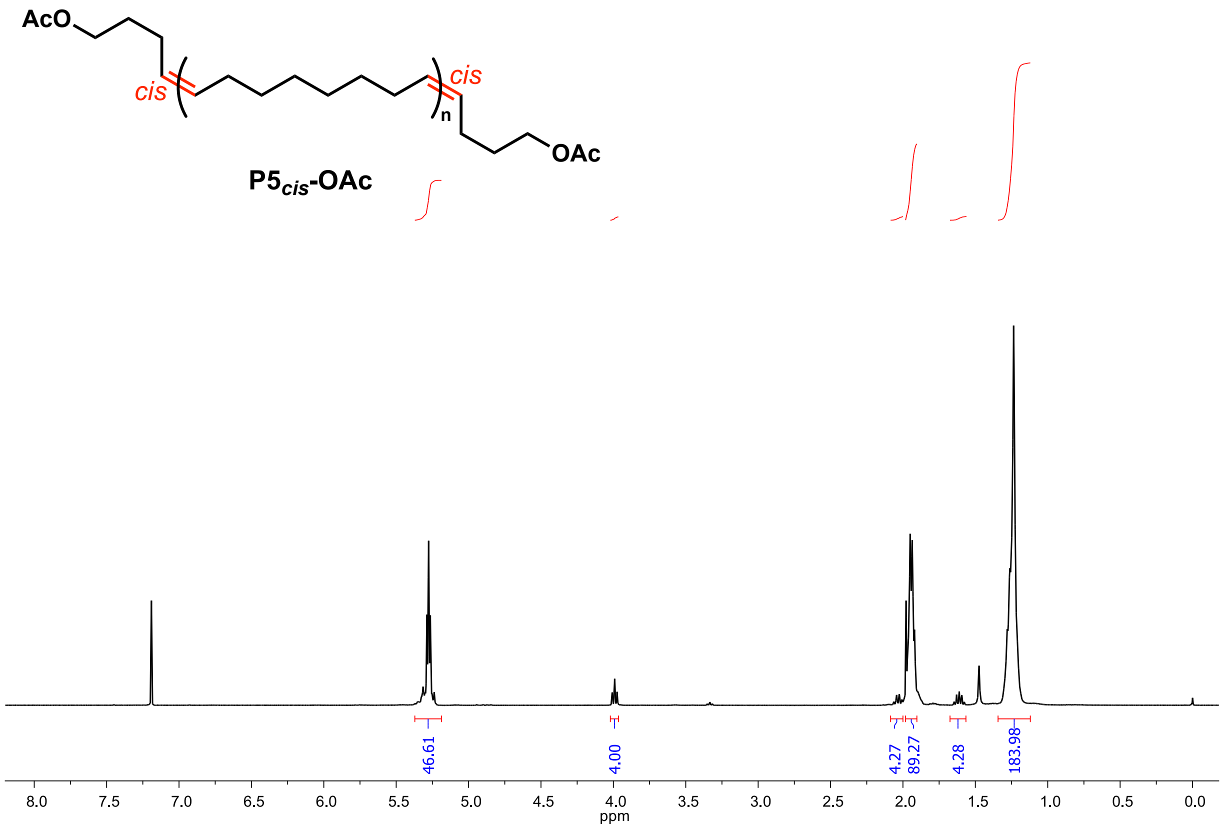

Figure S70. ^{1}H NMR (400 MHz, $CDCl_3$) spectrum of **P5*cis*-OAc**.

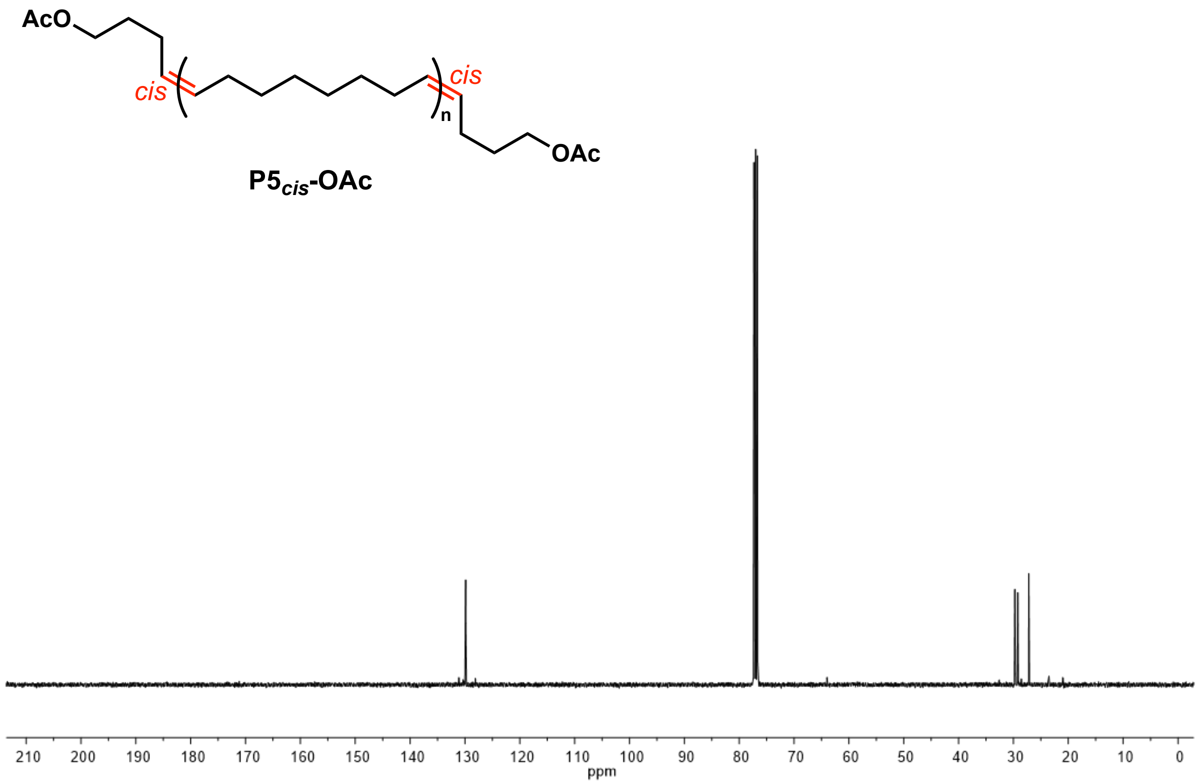

Figure S71. ^{13}C NMR (101 MHz, $CDCl_3$) spectrum of **P5*cis*-OAc**.

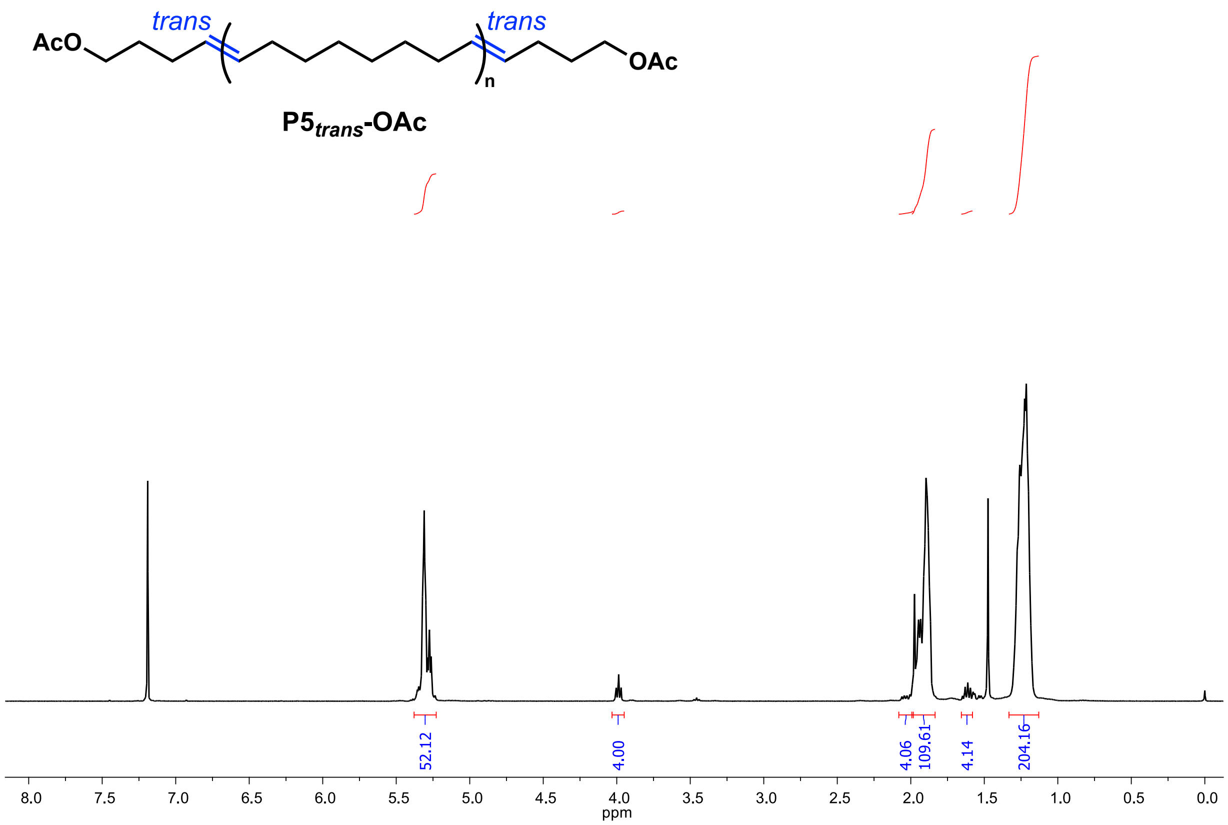

Figure S72. ^{1}H NMR (400 MHz, $CDCl_3$) spectrum of **P5$_{trans}$-OAc**.

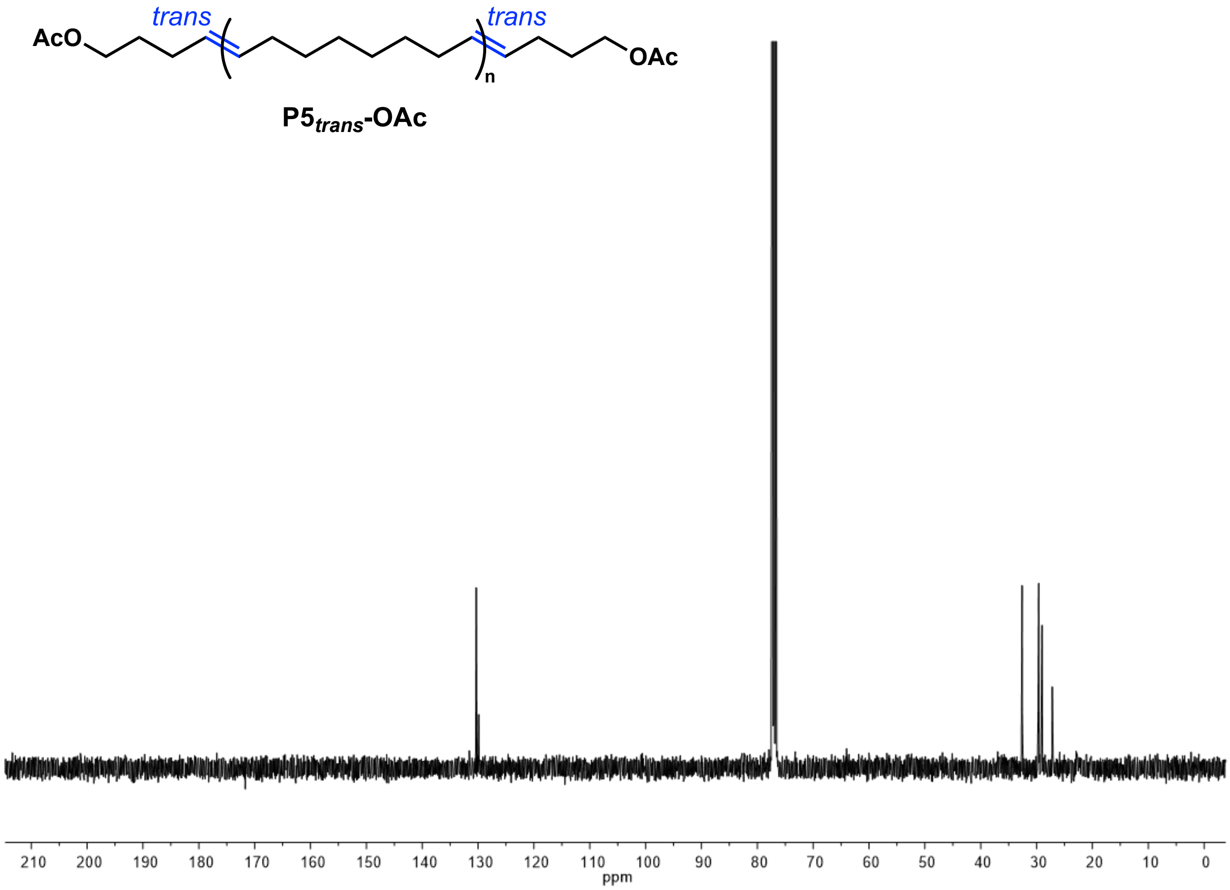

Figure S73. ^{13}C NMR (101 MHz, $CDCl_3$) spectrum of **P5*trans*-OAc**.

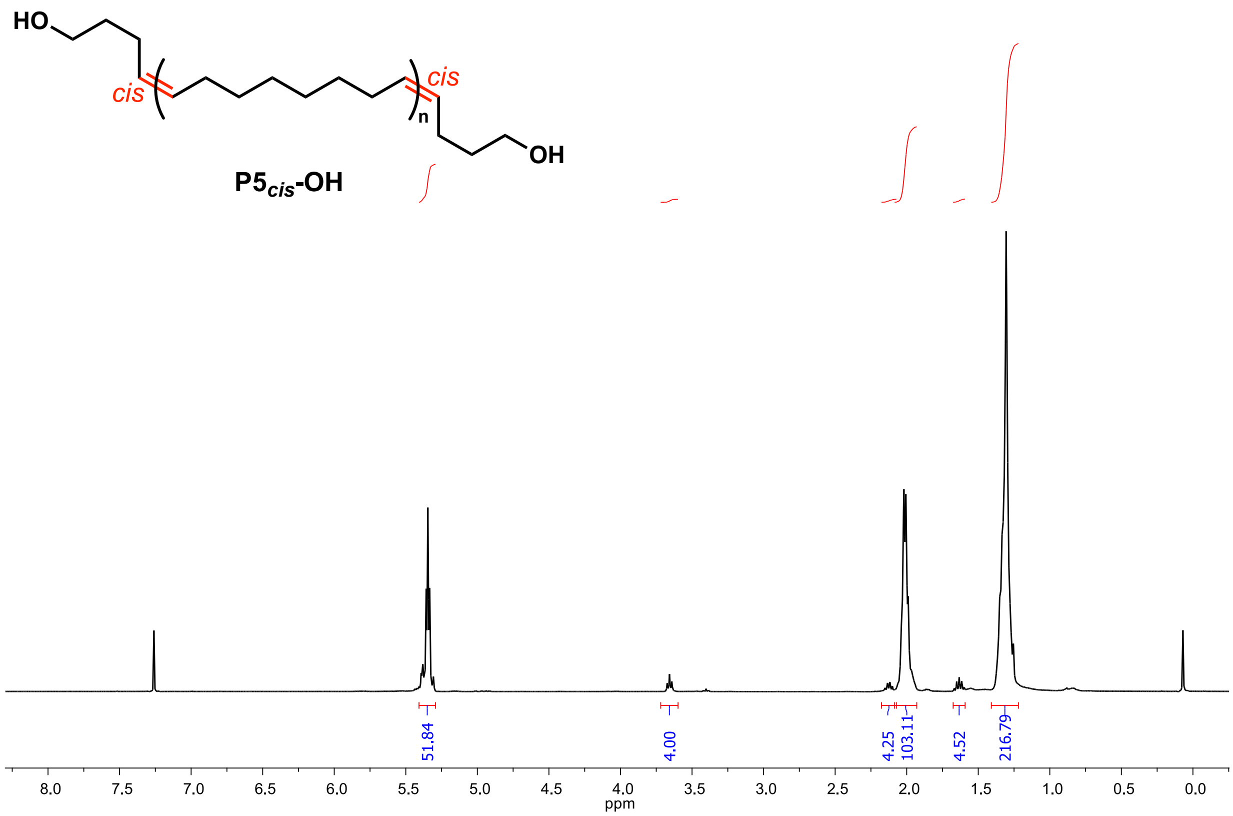

Figure S74. ^{1}H NMR (400 MHz, $CDCl_3$) spectrum of **P5$_{cis}$-OH**.

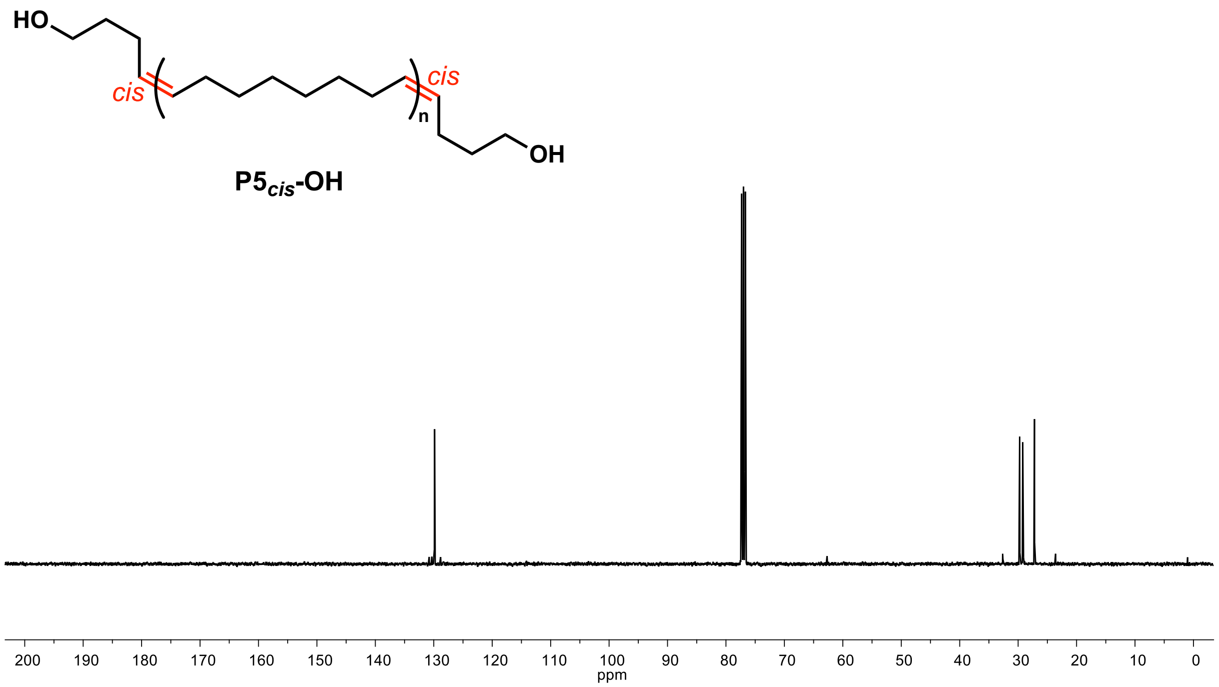

Figure S75. ^{13}C NMR (101 MHz, $CDCl_3$) spectrum of **P5$_{cis}$-OH**.

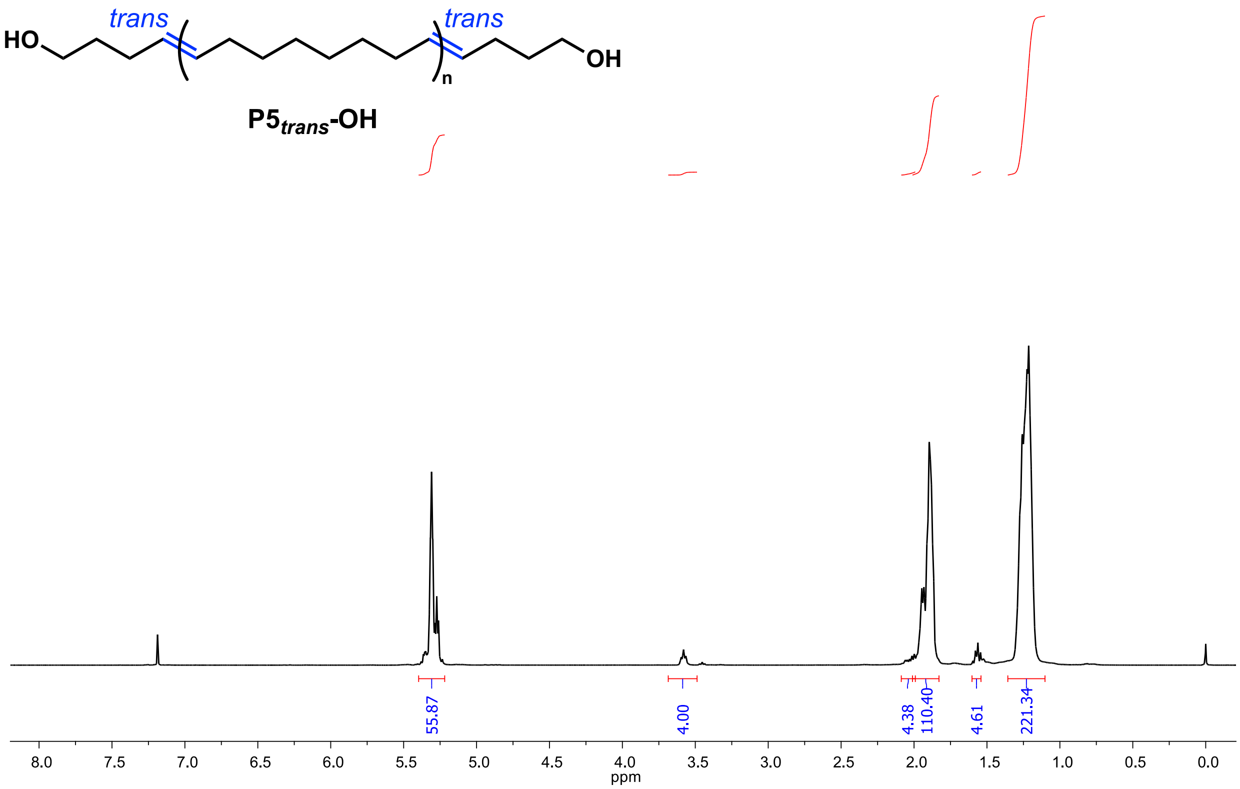

Figure S76. ^{1}H NMR (400 MHz, CDCl$_3$) spectrum of **P5$_{trans}$-OH**.

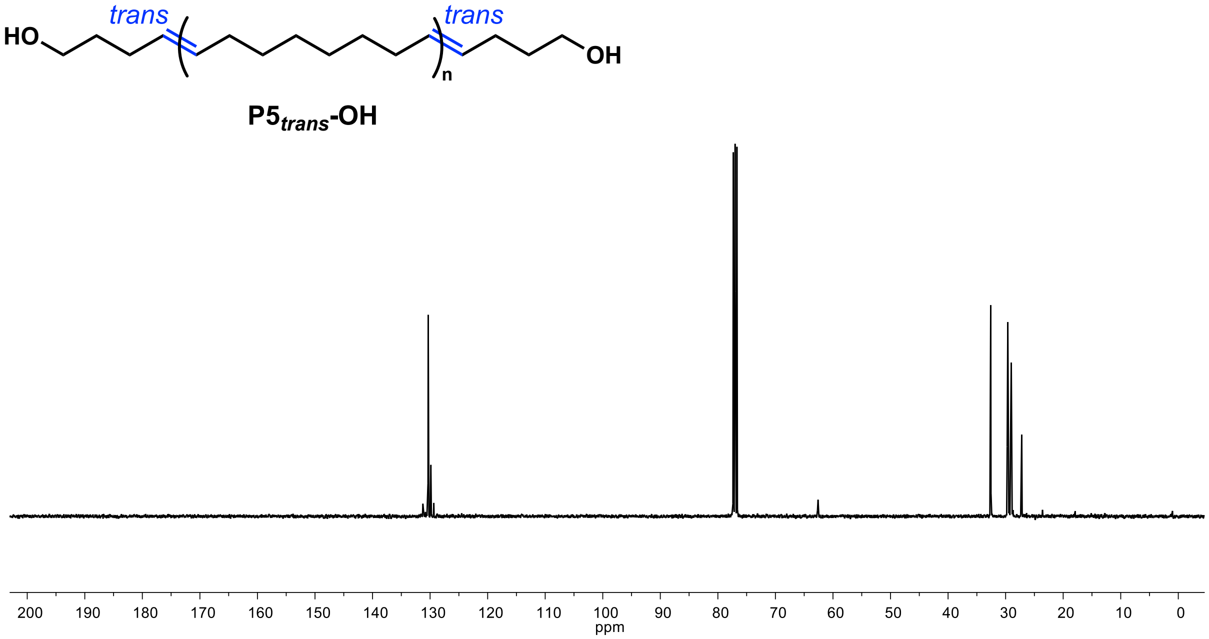

Figure S77. ^{13}C NMR (101 MHz, CDCl$_3$) spectrum of **P5$_{trans}$-OH**.

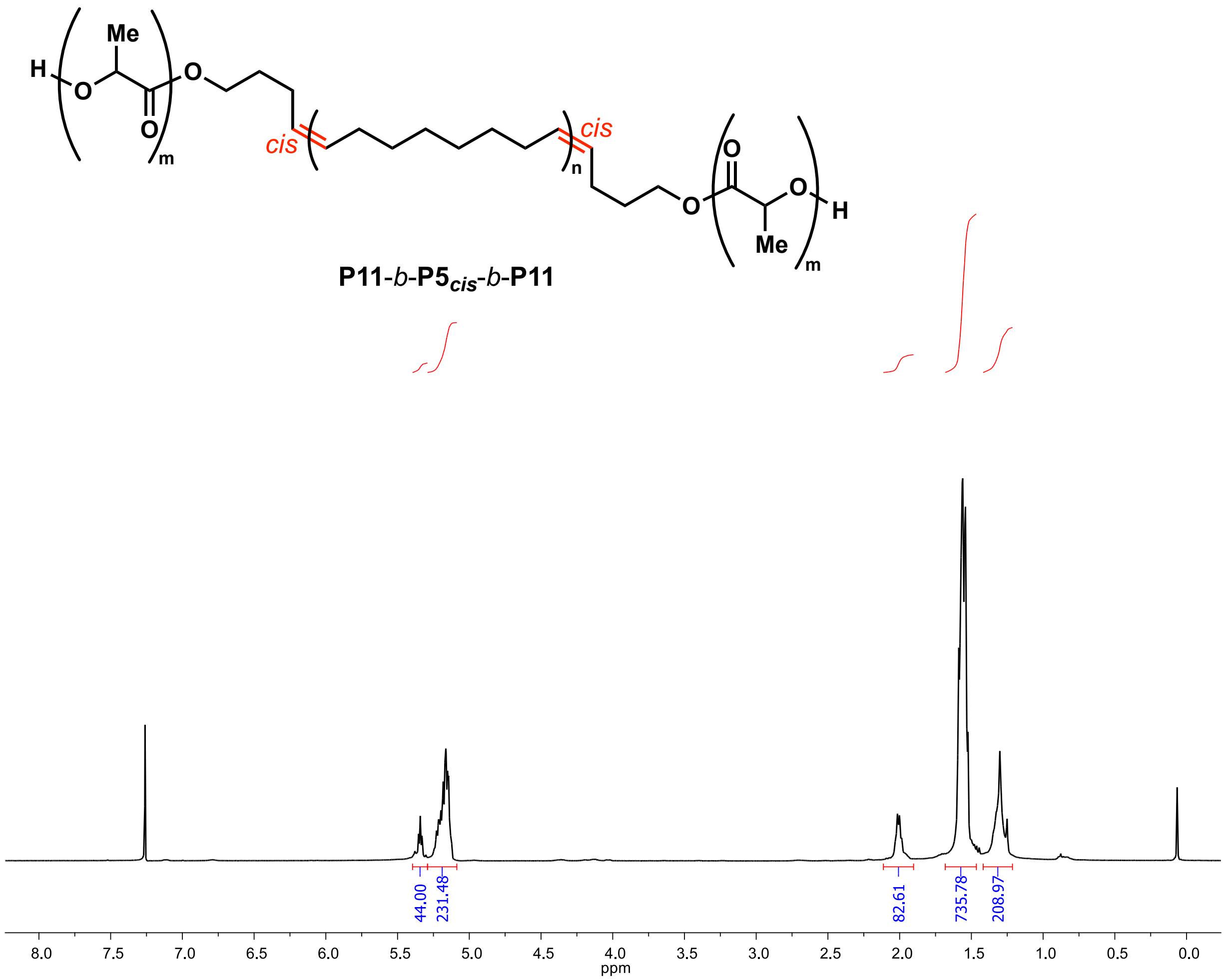

Figure S78. ^{1}H NMR (400 MHz, $CDCl_3$) spectrum of **P11**-*b*-**P5*cis***-*b*-**P11**.

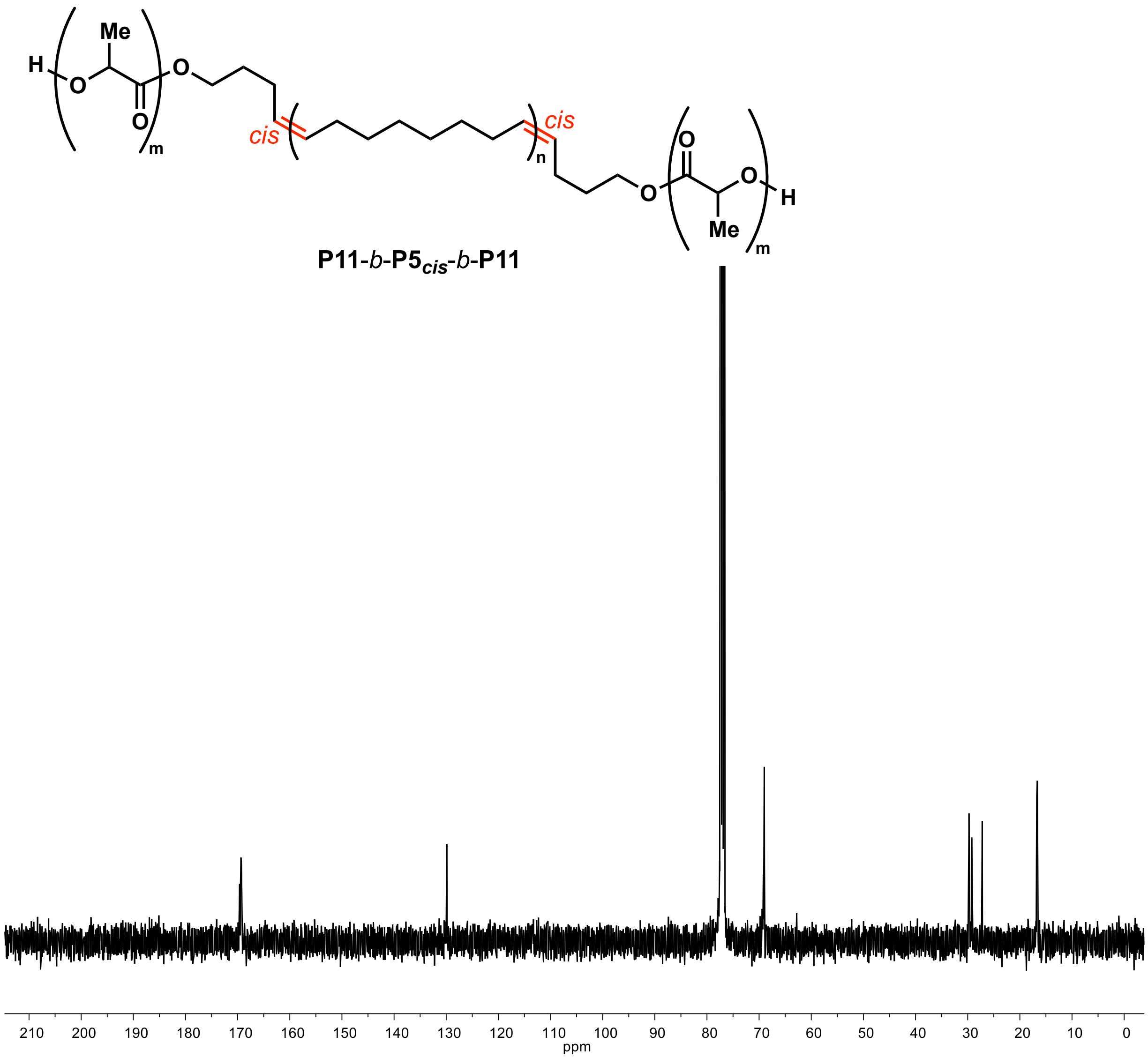

Figure S79. ^{13}C NMR (101 MHz, $CDCl_3$) spectrum of **P11**-*b*-**P5**$_{cis}$-*b*-**P11**.

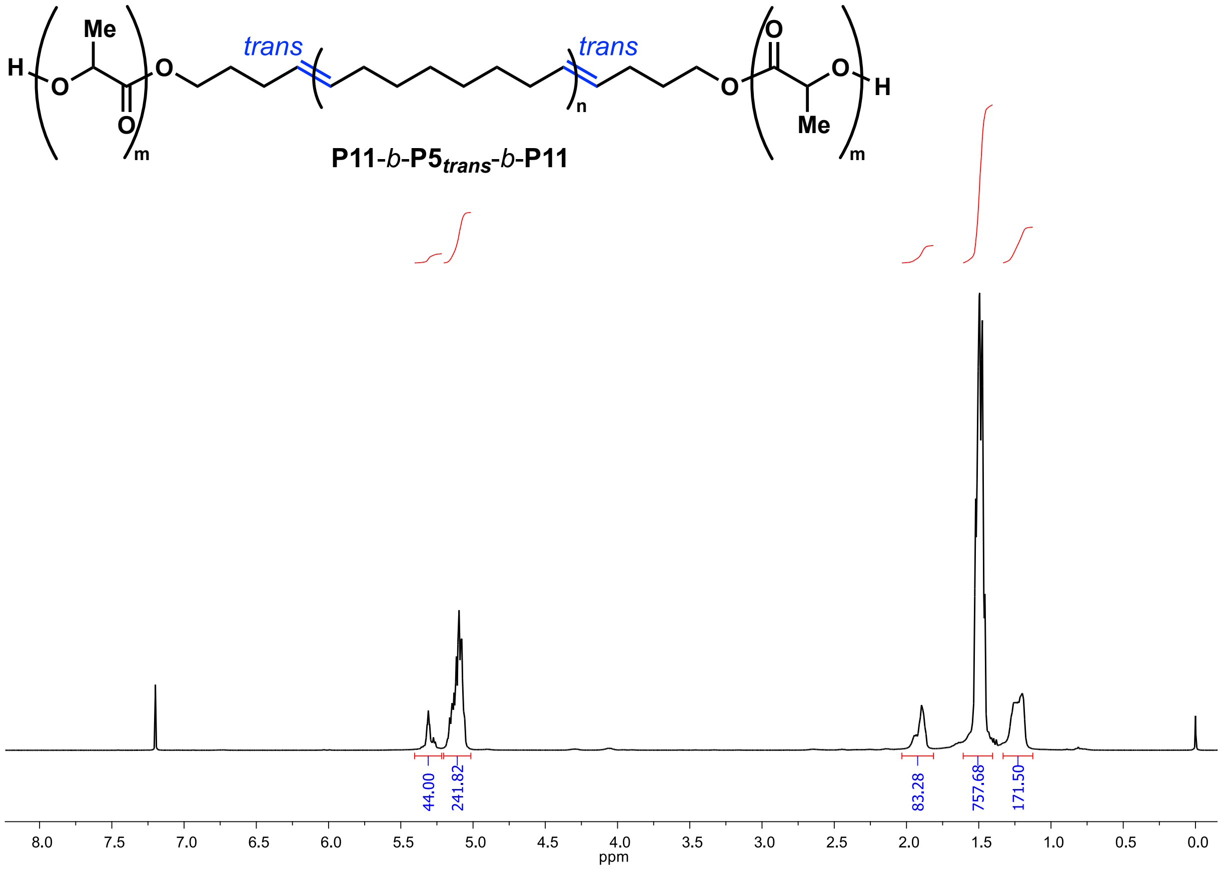

Figure S80. ^{1}H NMR (400 MHz, $CDCl_3$) spectrum of **P11**-*b*-**P5$_{trans}$**-*b*-**P11**.

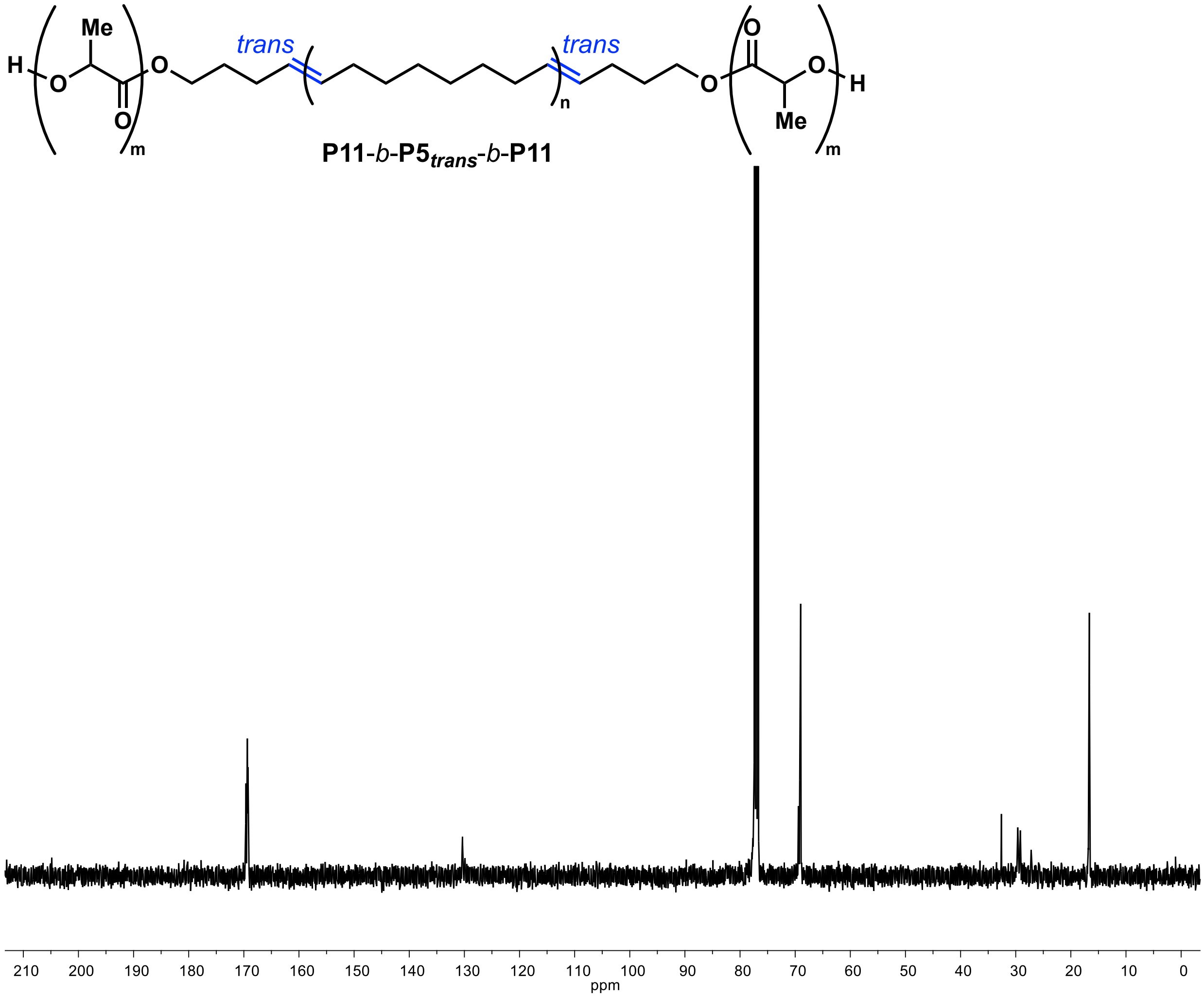

Figure S81. ^{13}C NMR (101 MHz, $CDCl_3$) spectrum of **P11**-*b*-**P5*trans***-*b*-**P11**.

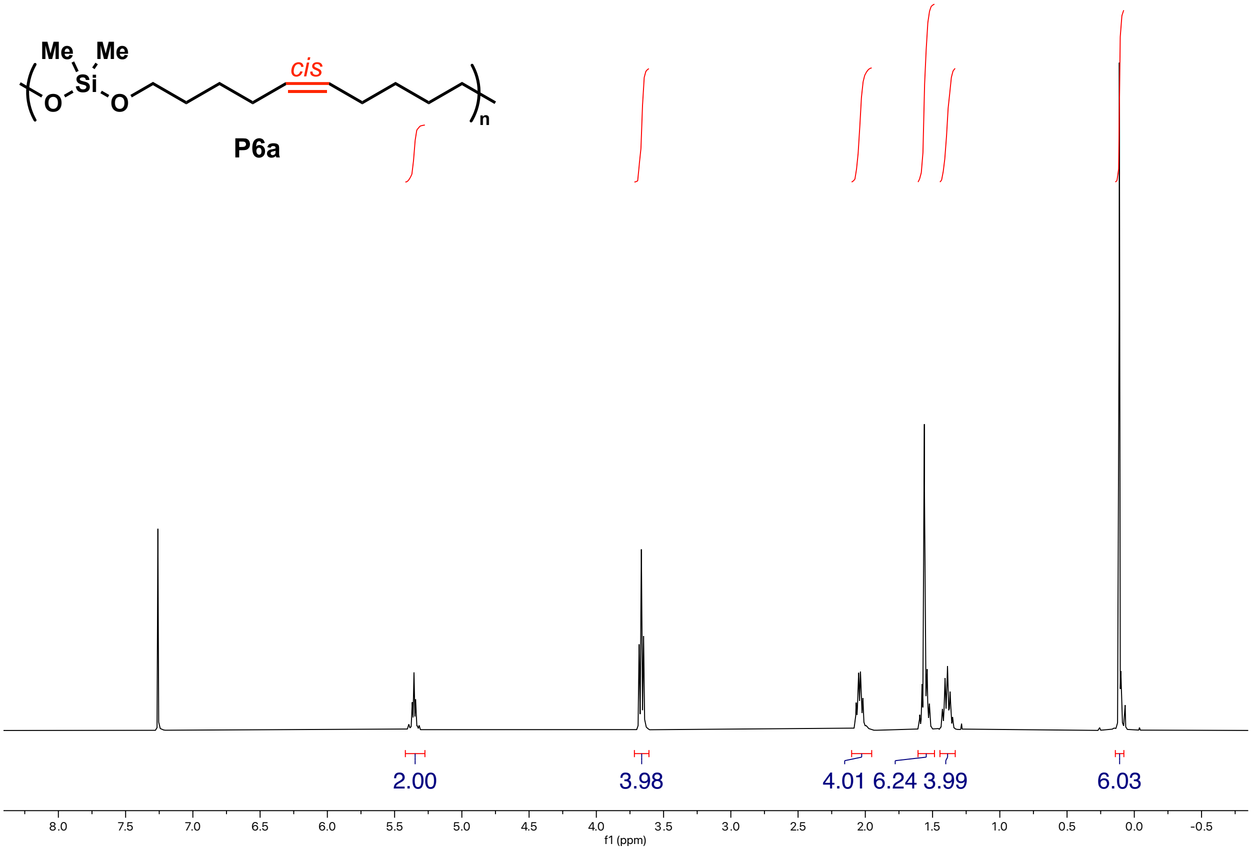

Figure S82. ^{1}H NMR (400 MHz, $CDCl_3$) spectrum of *cis*-rich compound **P6a**.

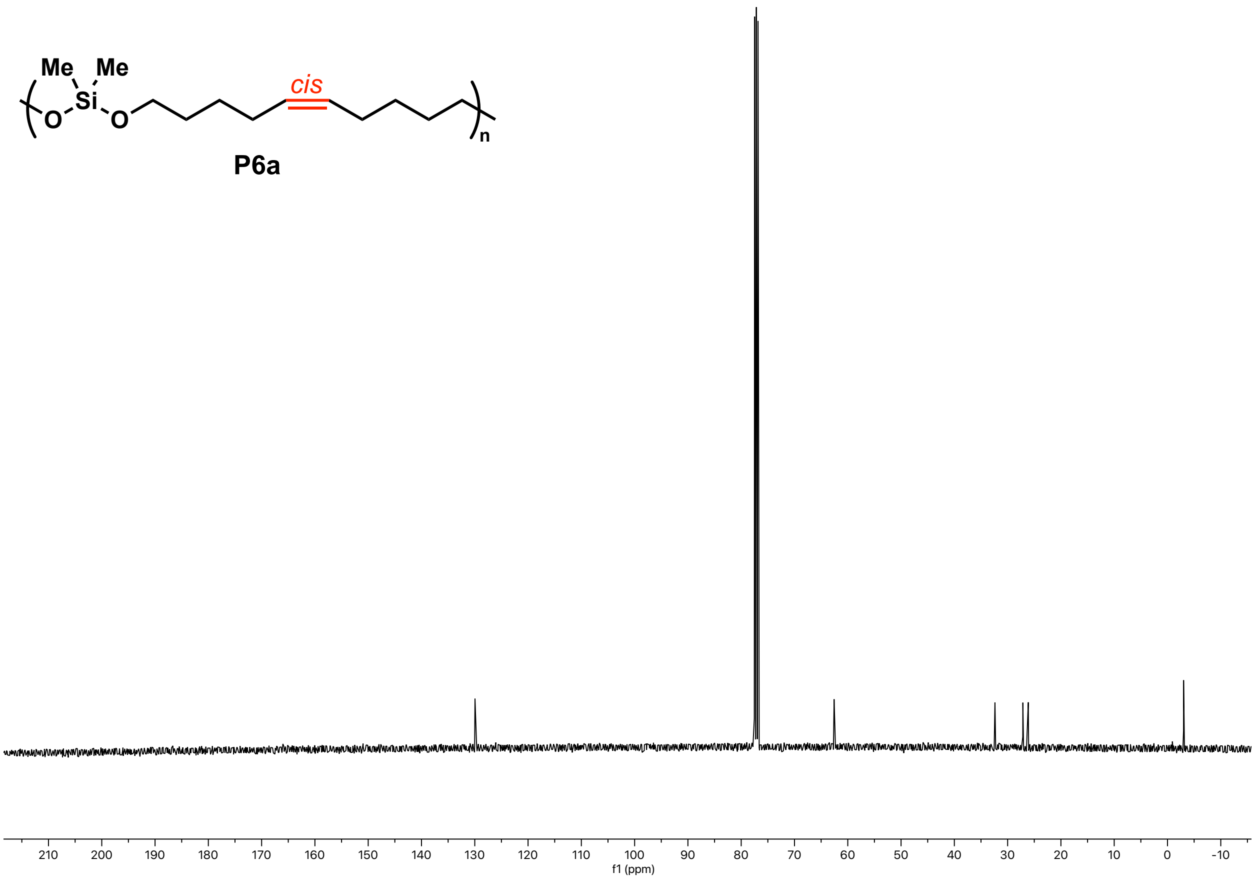

Figure S83. ^{13}C NMR (101 MHz, $CDCl_3$) spectrum of *cis*-rich compound **P6a**

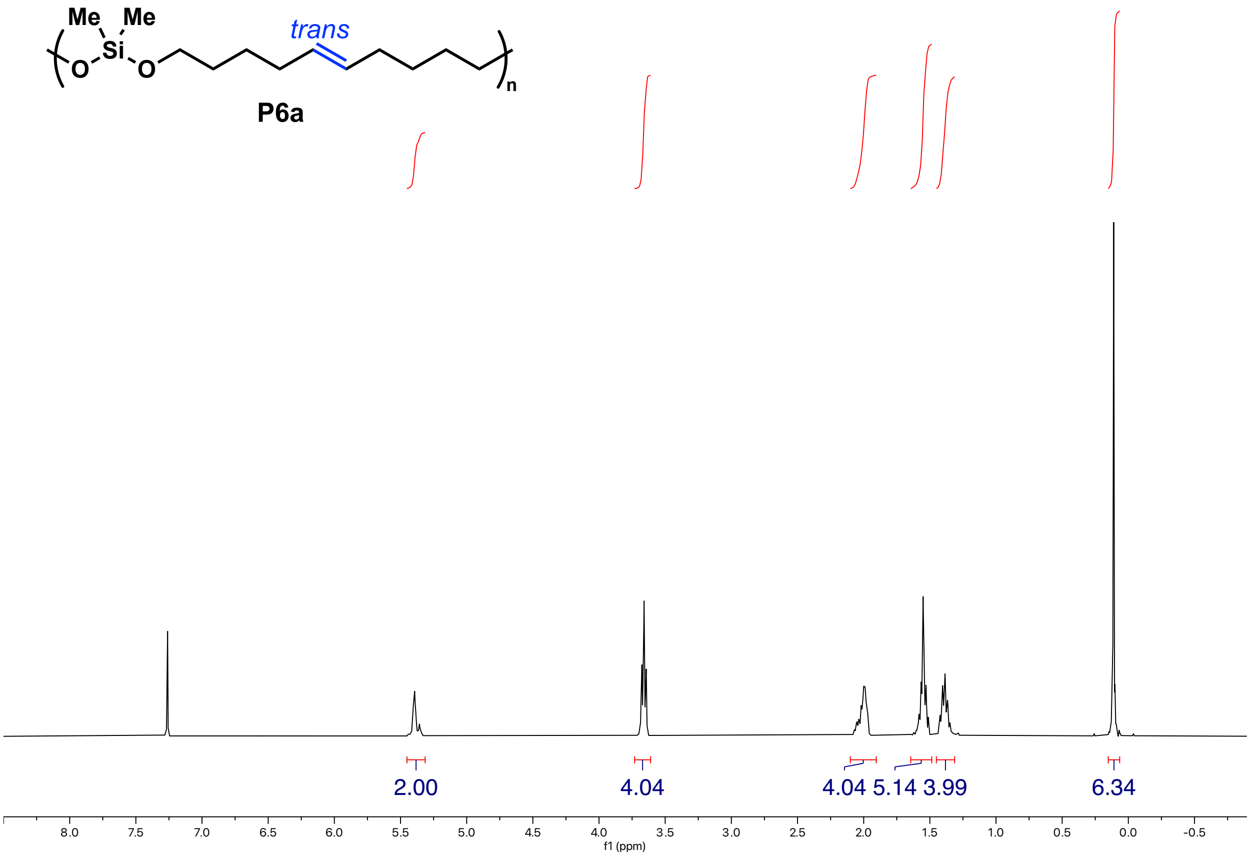

Figure S84. ^{1}H NMR (400 MHz, $CDCl_3$) spectrum of *trans*-rich compound **P6a**.

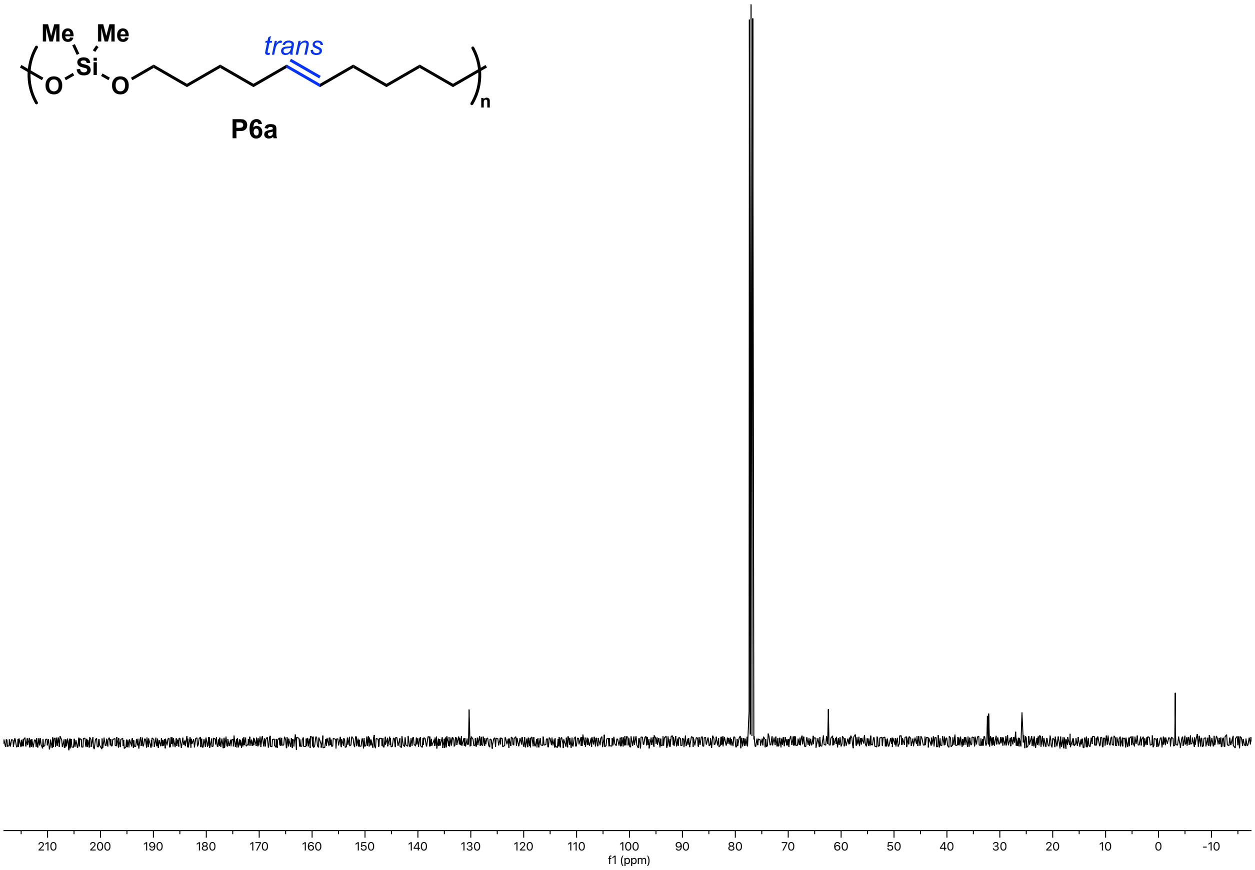

Figure S85. ^{13}C NMR (101 MHz, $CDCl_3$) spectrum of *trans*-rich compound **P6a**

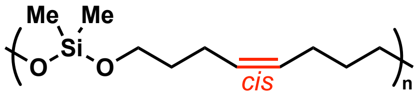

P6b

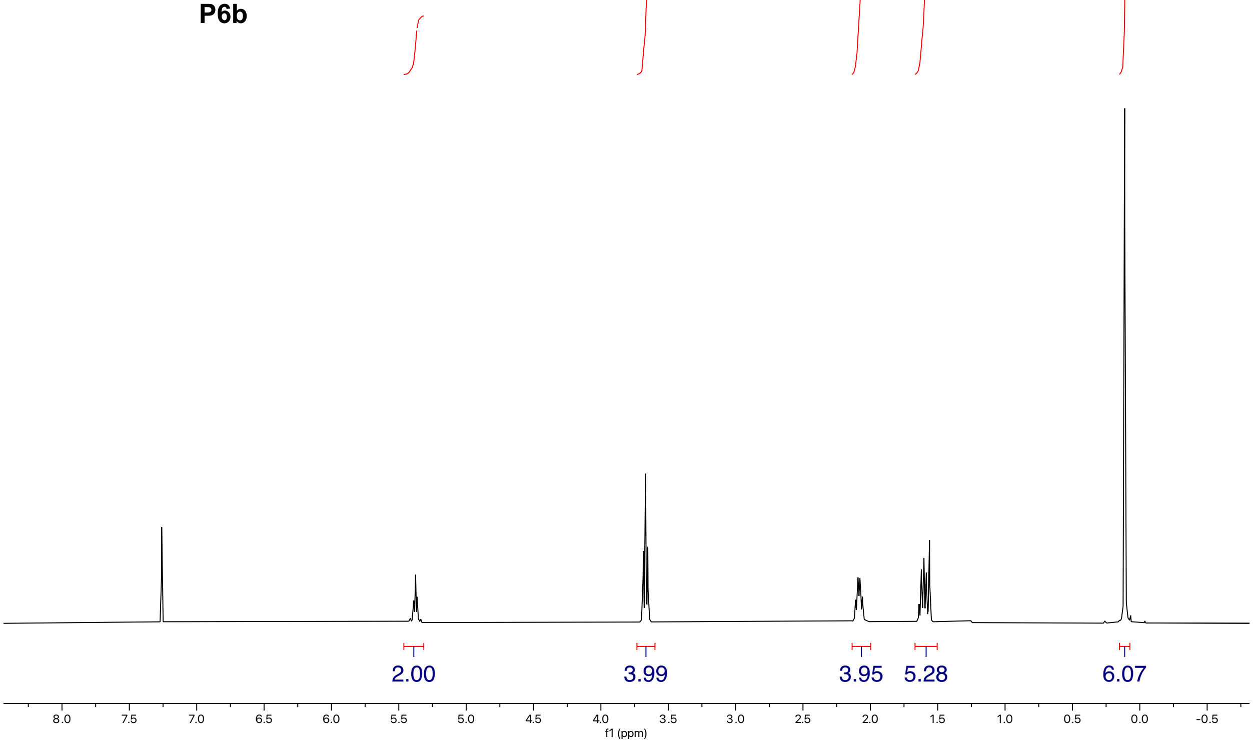

Figure S86. ^{1}H NMR (400 MHz, $CDCl_3$) spectrum of *cis*-rich compound **P6b**.

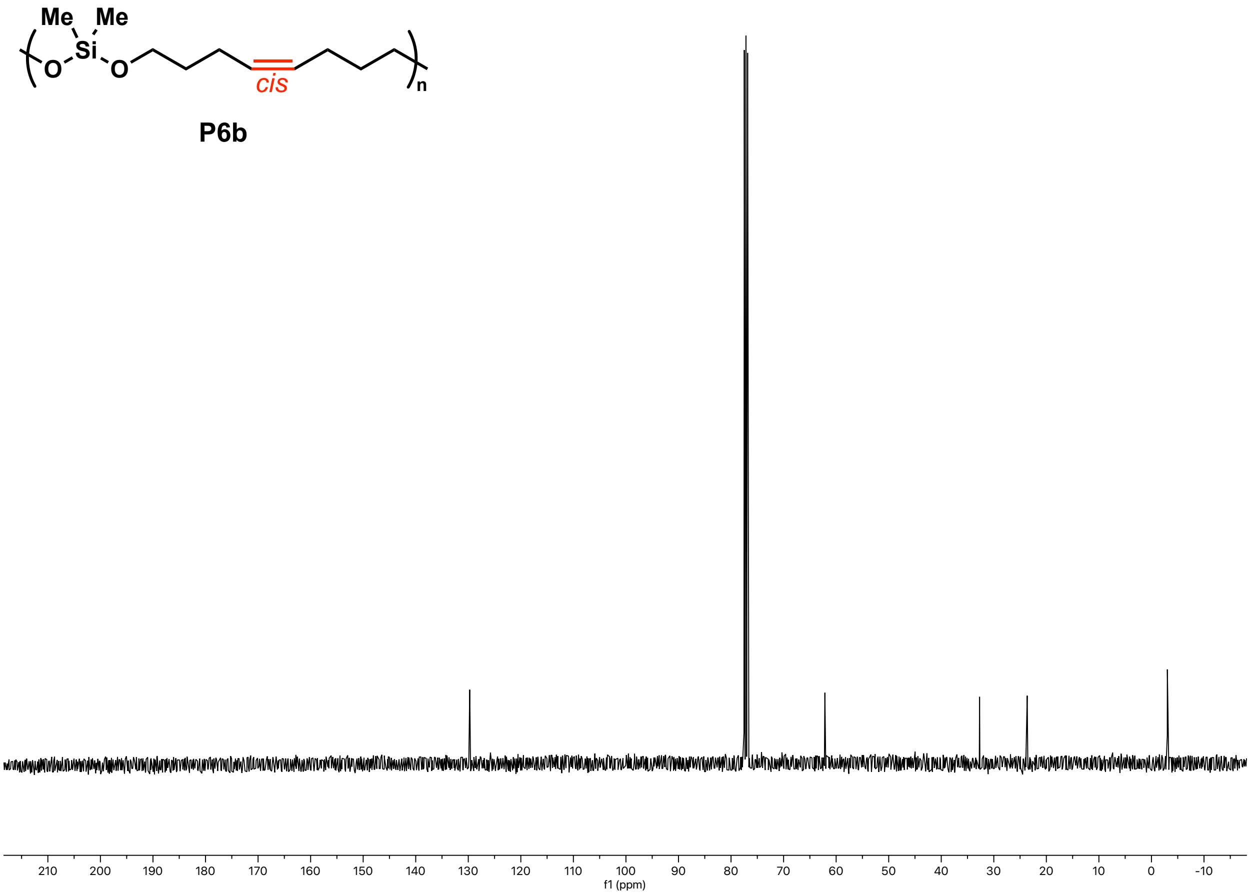

Figure S87. ^{13}C NMR (101 MHz, $CDCl_3$) spectrum of *cis*-rich **P6b**.

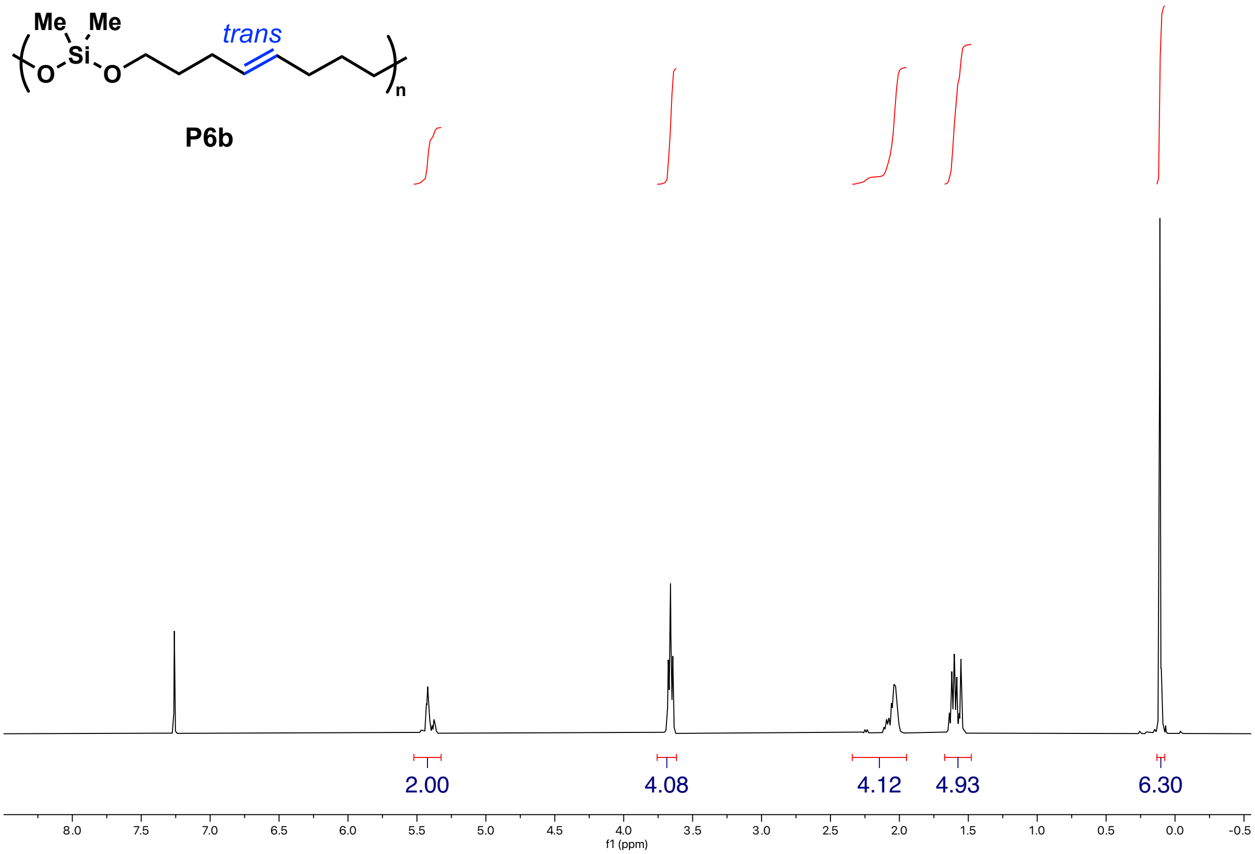

Figure S88. ^{1}H NMR (400 MHz, $CDCl_3$) spectrum of *trans*-rich compound **P6b**.

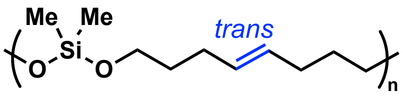

P6b

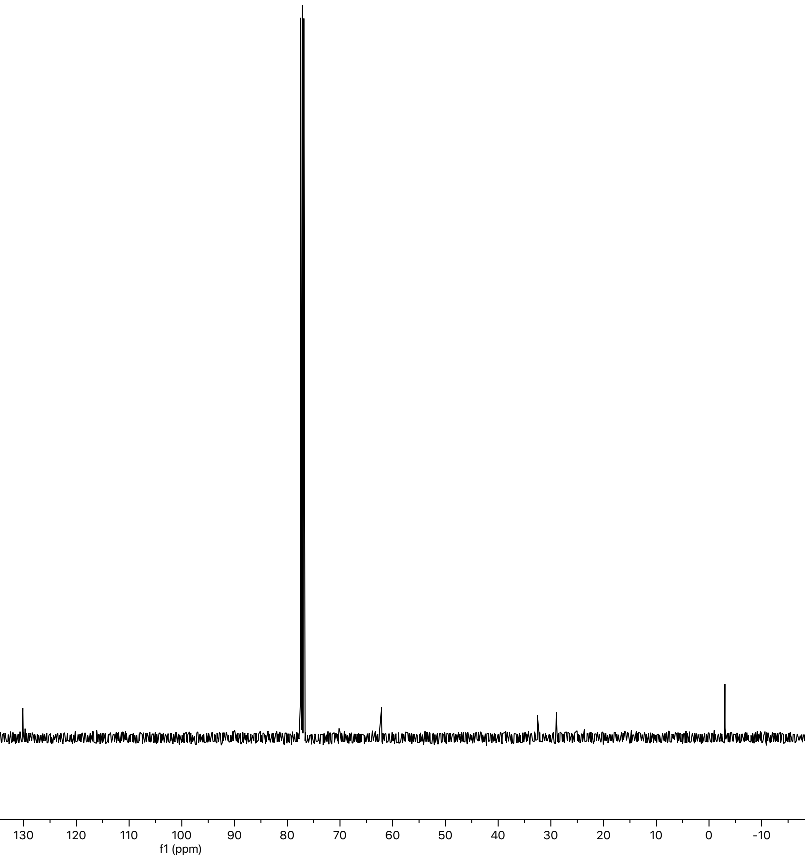

Figure S89. ^{13}C NMR (101 MHz, $CDCl_3$) spectrum of *trans*-rich **P6b**.

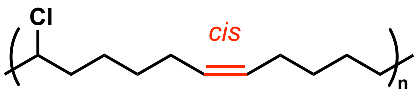

P7a

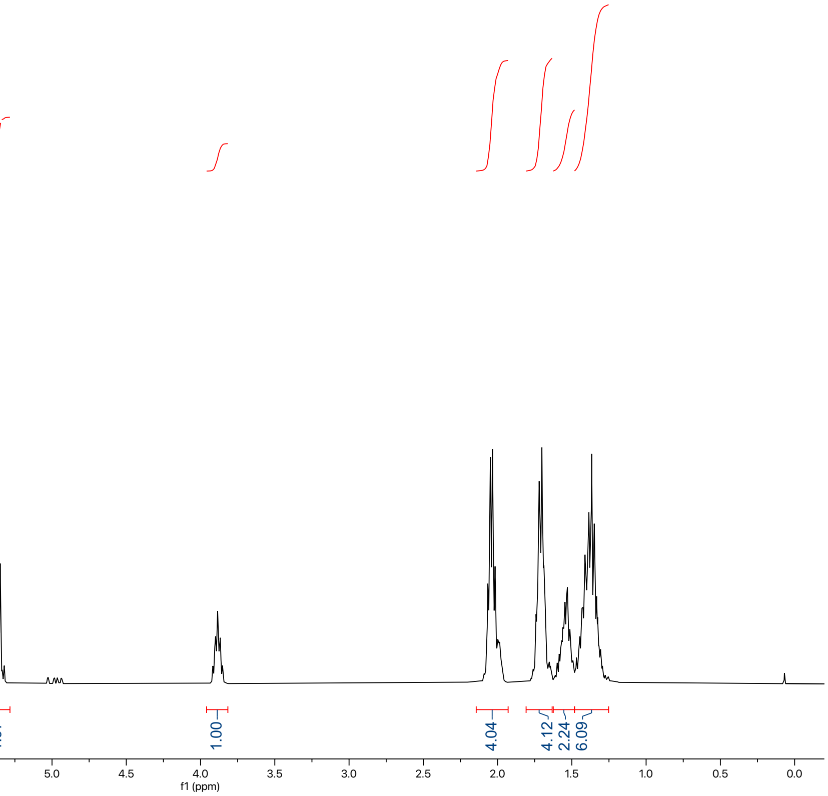

Figure S90. ^{1}H NMR (400 MHz, $CDCl_3$) spectrum of *cis*-rich compound **P7a**.

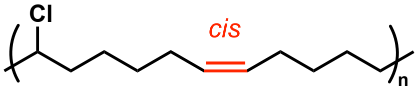

P7a

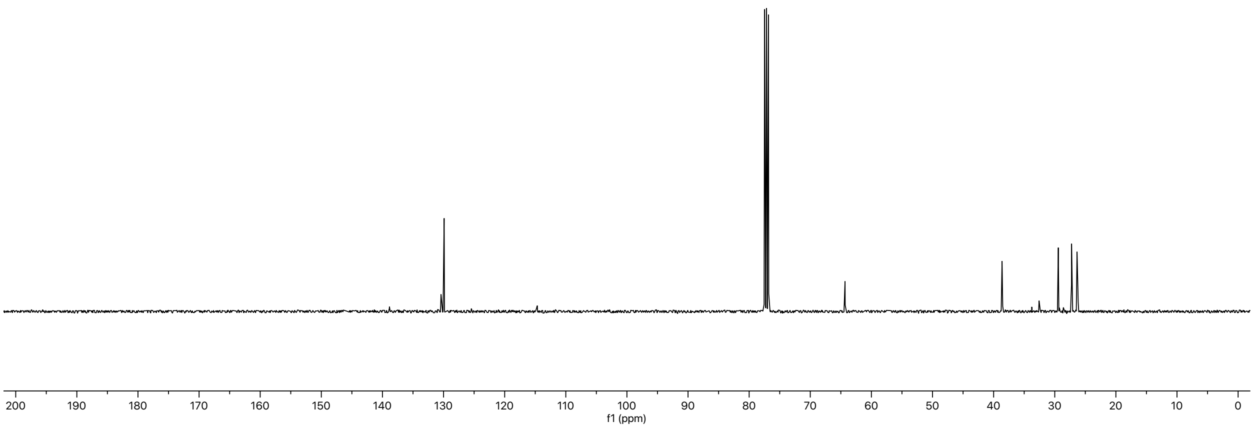

Figure S91. ^{13}C NMR (101 MHz, $CDCl_3$) spectrum of *cis*-rich **P7a**.

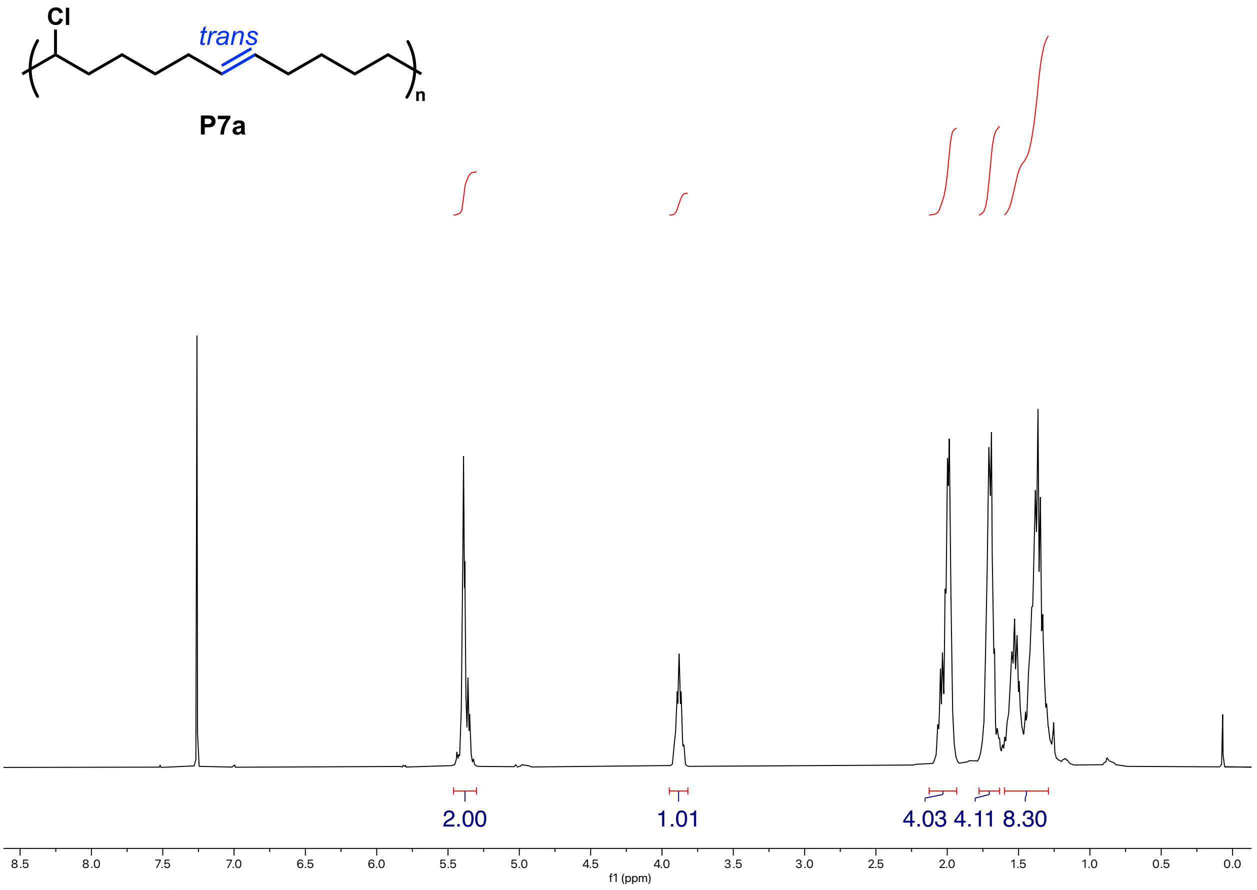

Figure S92. ^{1}H NMR (400 MHz, $CDCl_3$) spectrum of *trans*-rich compound **P7a**.

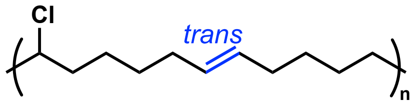

P7a

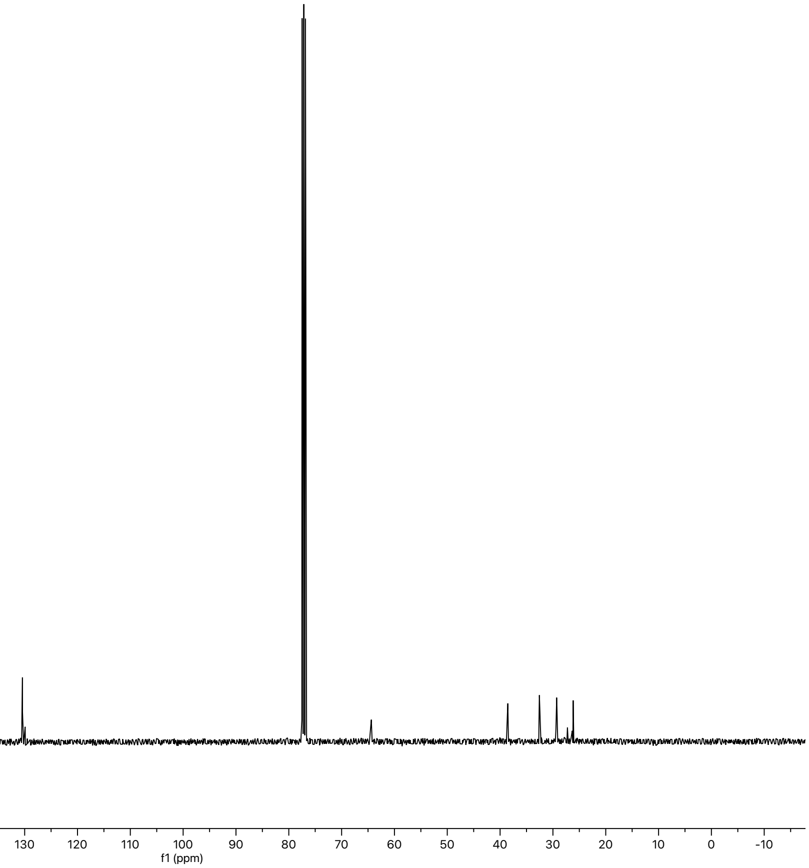

Figure S93. ^{13}C NMR (101 MHz, $CDCl_3$) spectrum of *trans*-rich **P7a**.

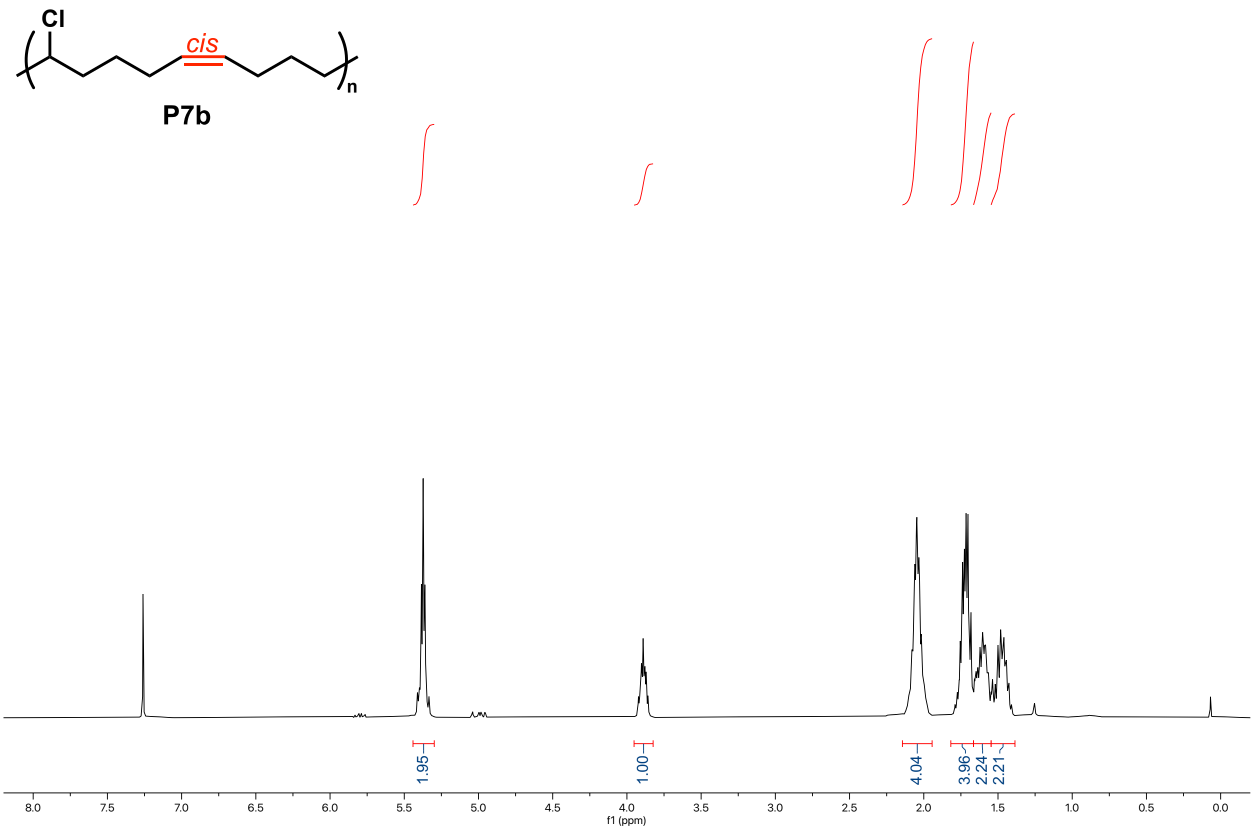

Figure S94. ^{1}H NMR (400 MHz, $CDCl_3$) spectrum of *cis*-rich compound **P7b**.

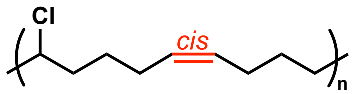

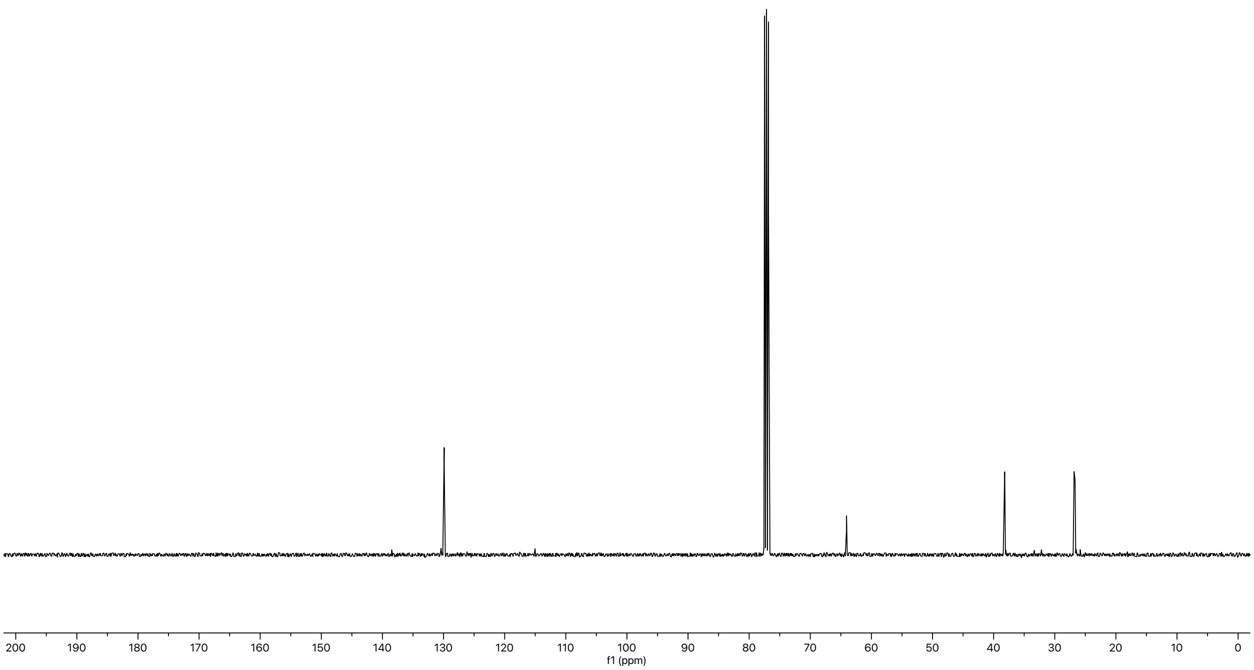

Figure S95. ^{13}C NMR (101 MHz, $CDCl_3$) spectrum of *cis*-rich **P7b**.

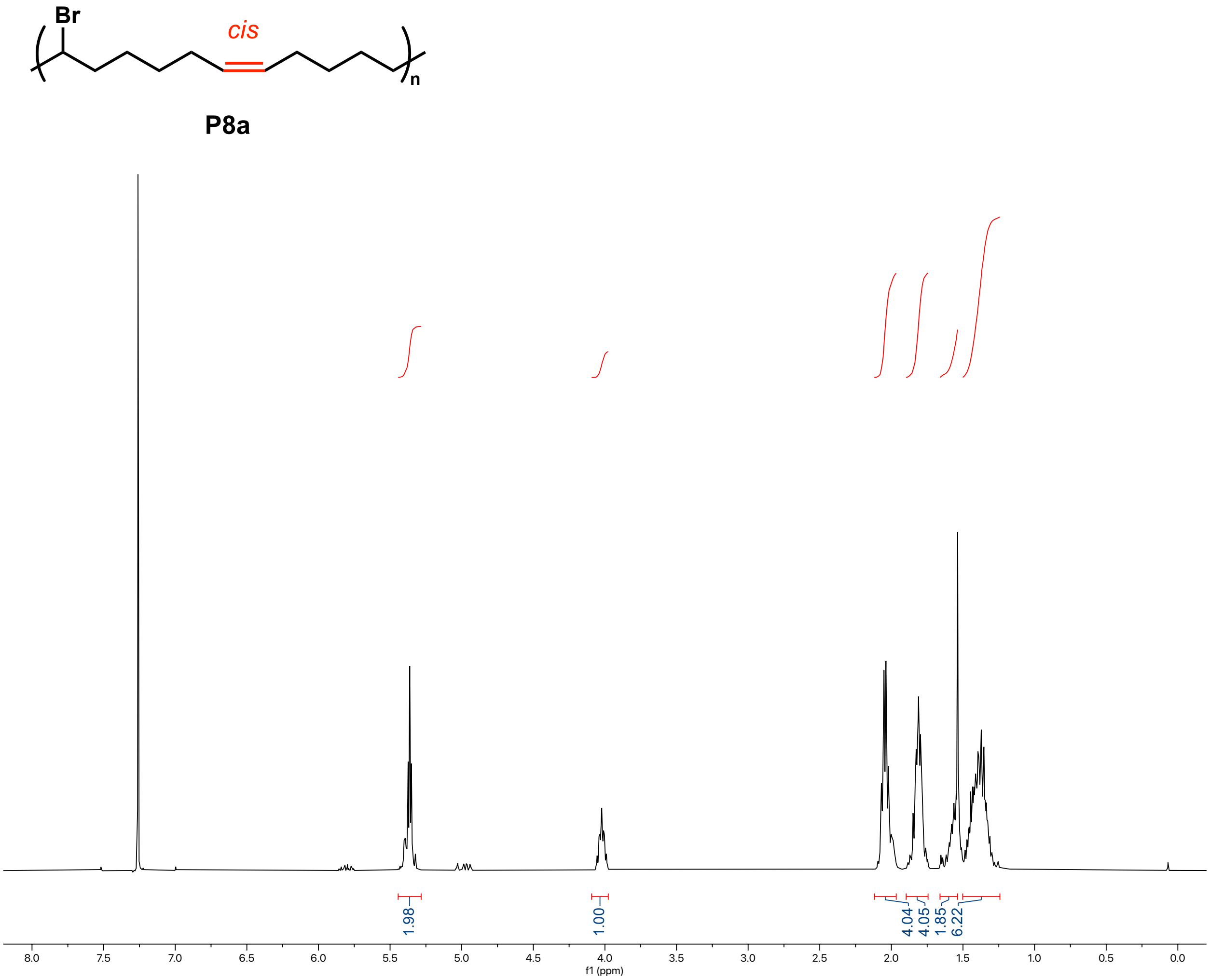

Figure S96. ^{1}H NMR (400 MHz, $CDCl_3$) spectrum of *cis*-rich compound **P8a**.

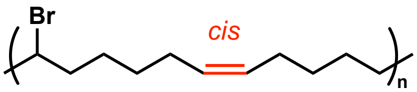

P8a

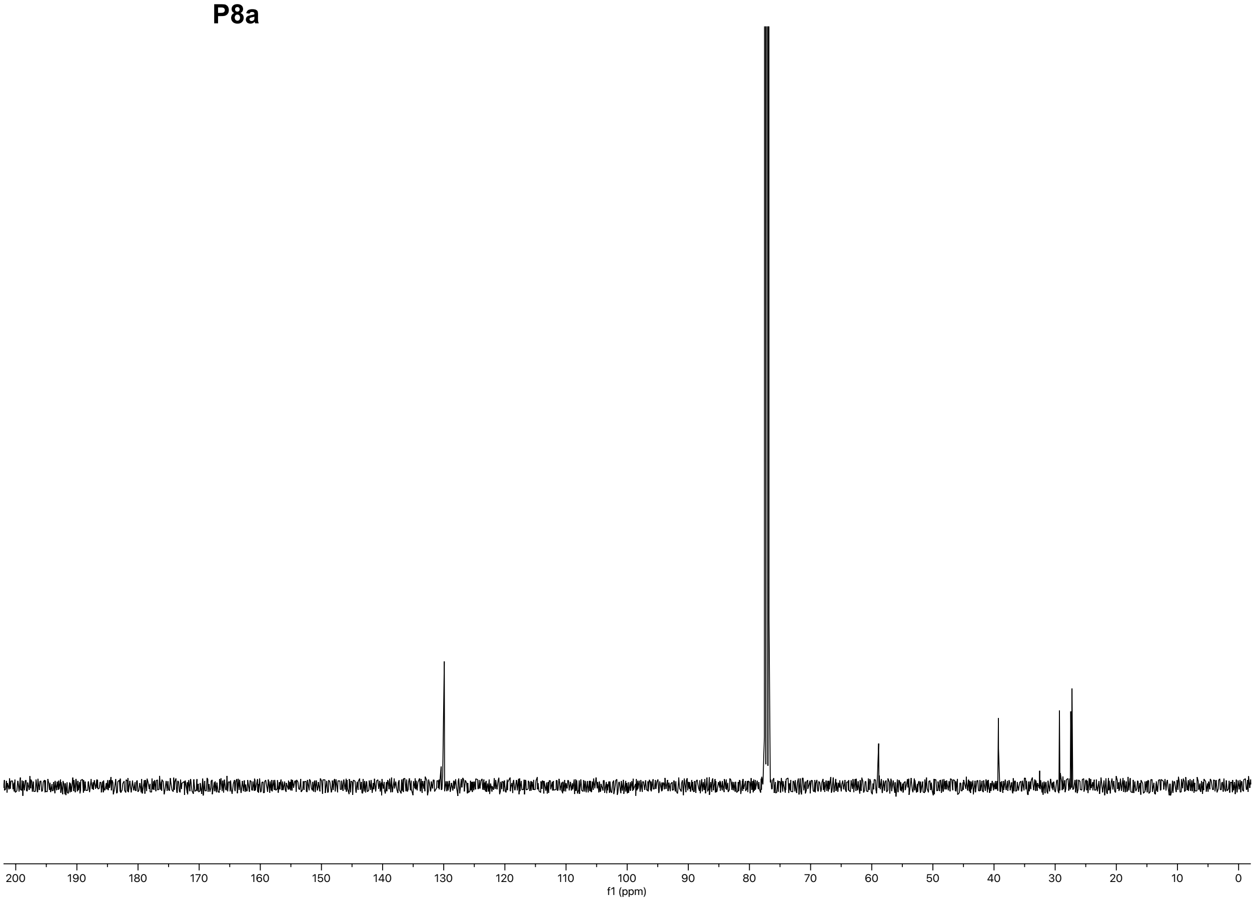

Figure S97. ^{13}C NMR (101 MHz, $CDCl_3$) spectrum of *cis*-rich **P8a**.

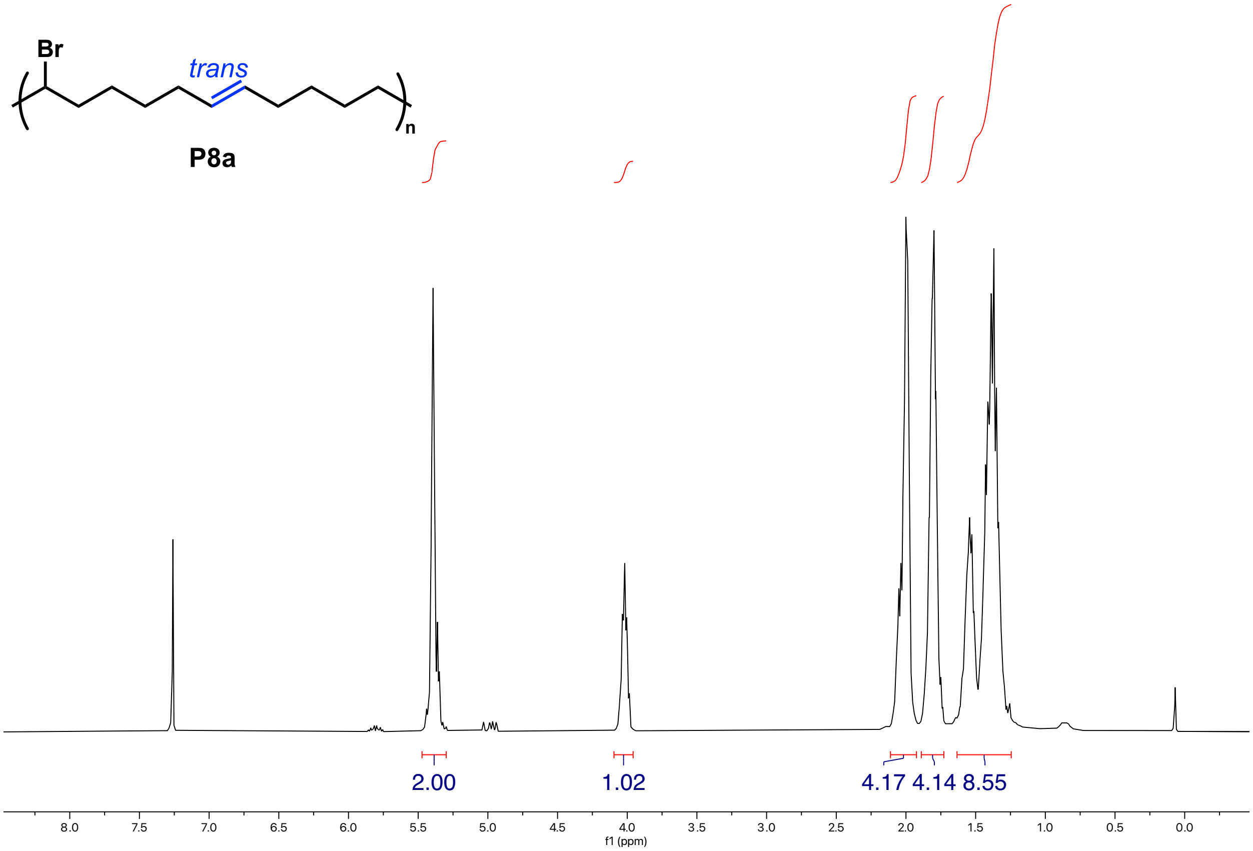

Figure S98. ^{1}H NMR (400 MHz, $CDCl_3$) spectrum of *trans*-rich compound **P8a**.

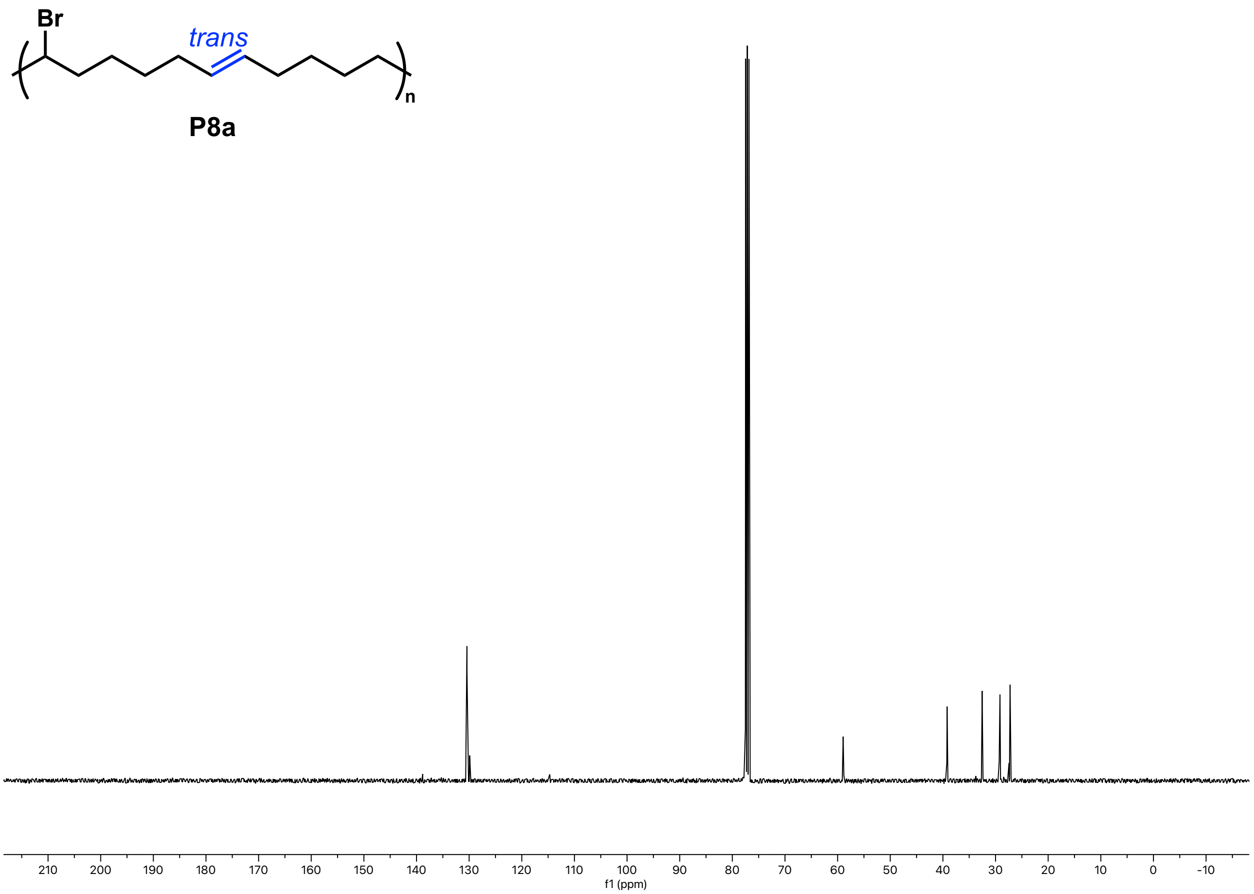

Figure S99. ^{13}C NMR (101 MHz, $CDCl_3$) spectrum of *trans*-rich **P8a**.

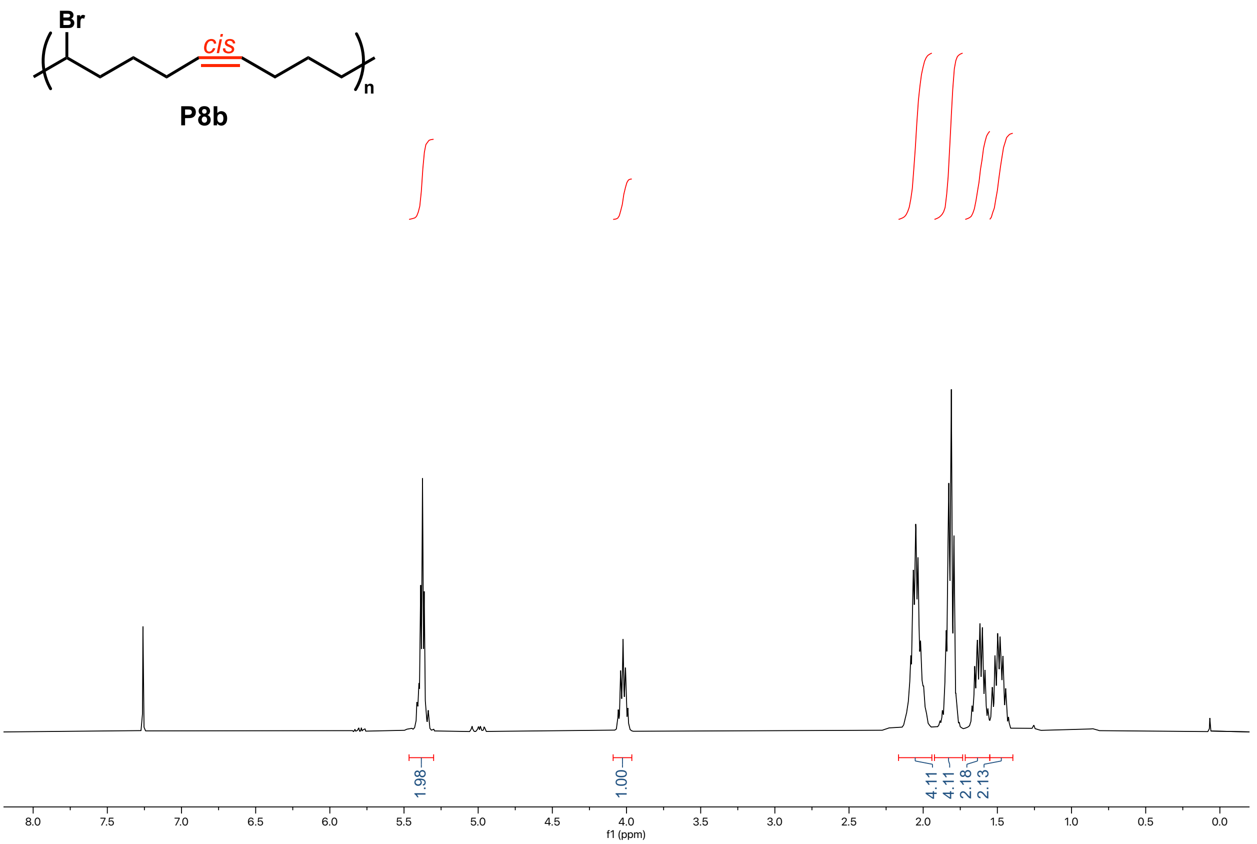

Figure S100. ^{1}H NMR (400 MHz, $CDCl_3$) spectrum of *cis*-rich compound **P8b**.

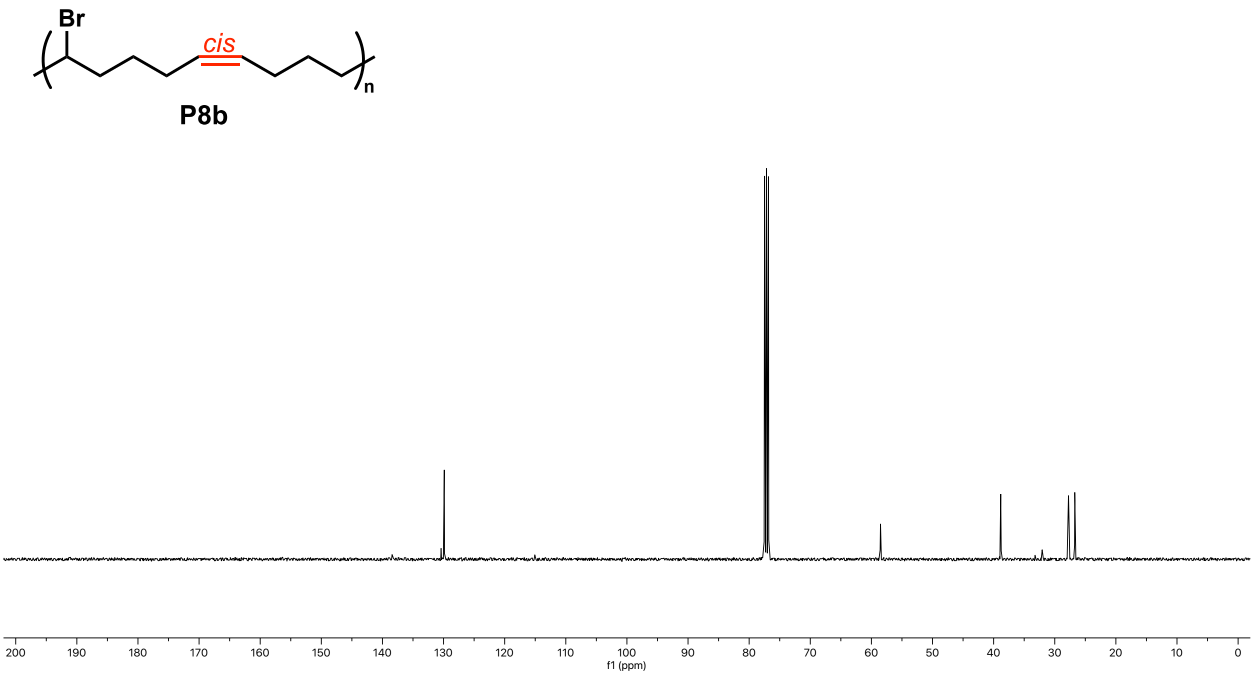

Figure S101. ^{13}C NMR (101 MHz, $CDCl_3$) spectrum of *cis*-rich **P8b**.

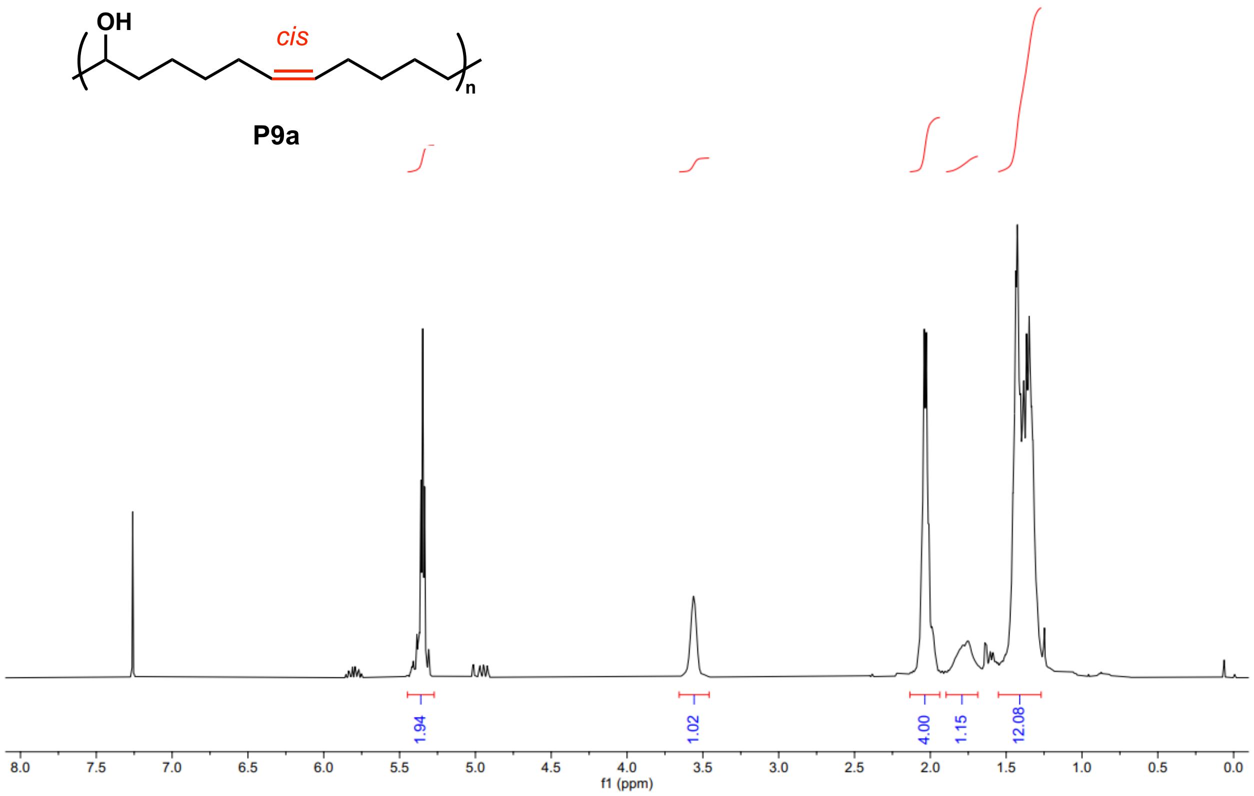

Figure S102. ^{1}H NMR (400 MHz, $CDCl_3$) spectrum of *cis*-rich compound **P9a**.

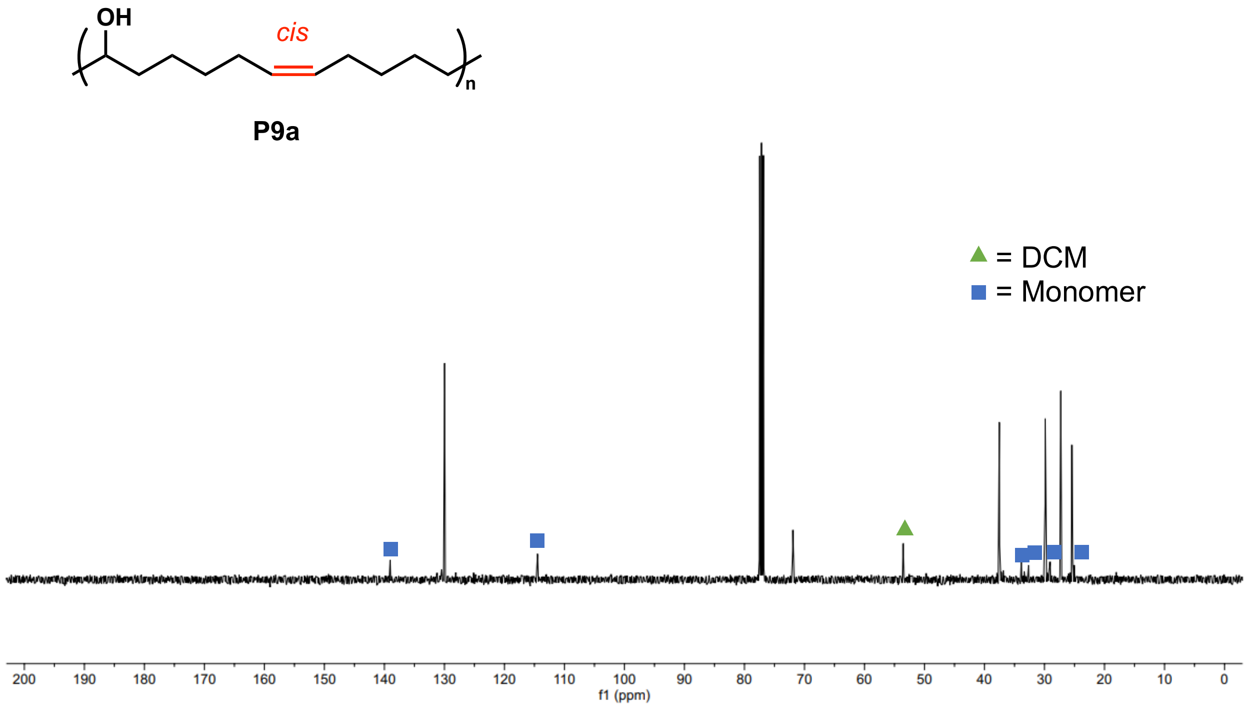

Figure S103. ^{13}C NMR (101 MHz, $CDCl_3$) spectrum of *cis*-rich **P9a** (NOTE: oligomer **P9a** could not be precipitated and separated from unreacted monomer **9a** (blue square labels).

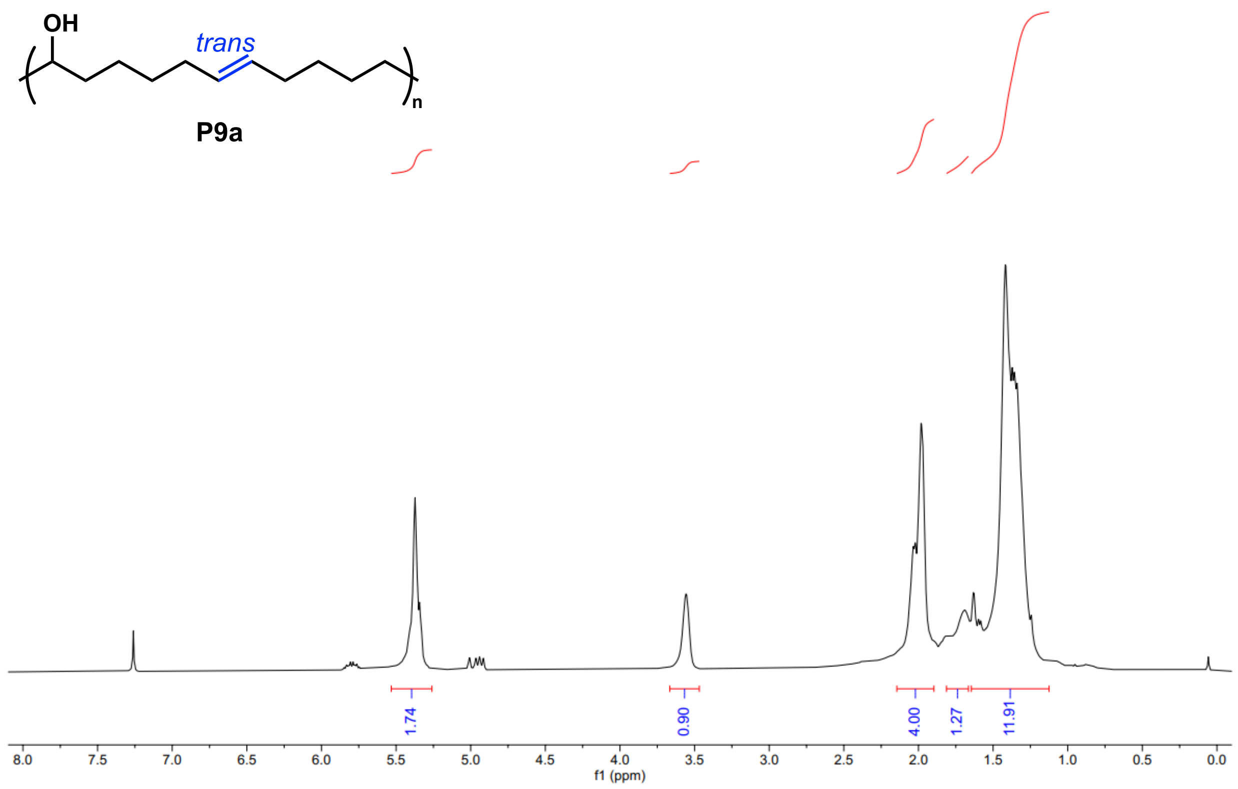

Figure S104. ^{1}H NMR (400 MHz, $CDCl_3$) spectrum of *trans*-rich compound **P9a**.

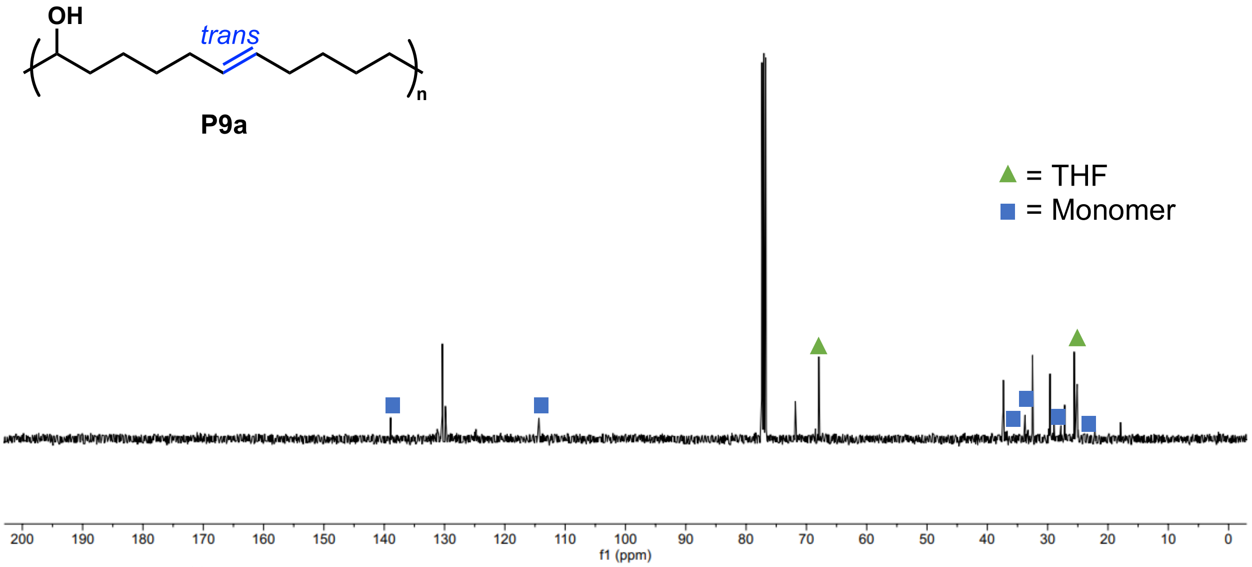

Figure S105. ^{13}C NMR (101 MHz, $CDCl_3$) spectrum of *trans*-rich **P9a** (NOTE: oligomer *trans*-**P9a** could not be precipitated and separated from unreacted monomer **9a** (blue square labels).

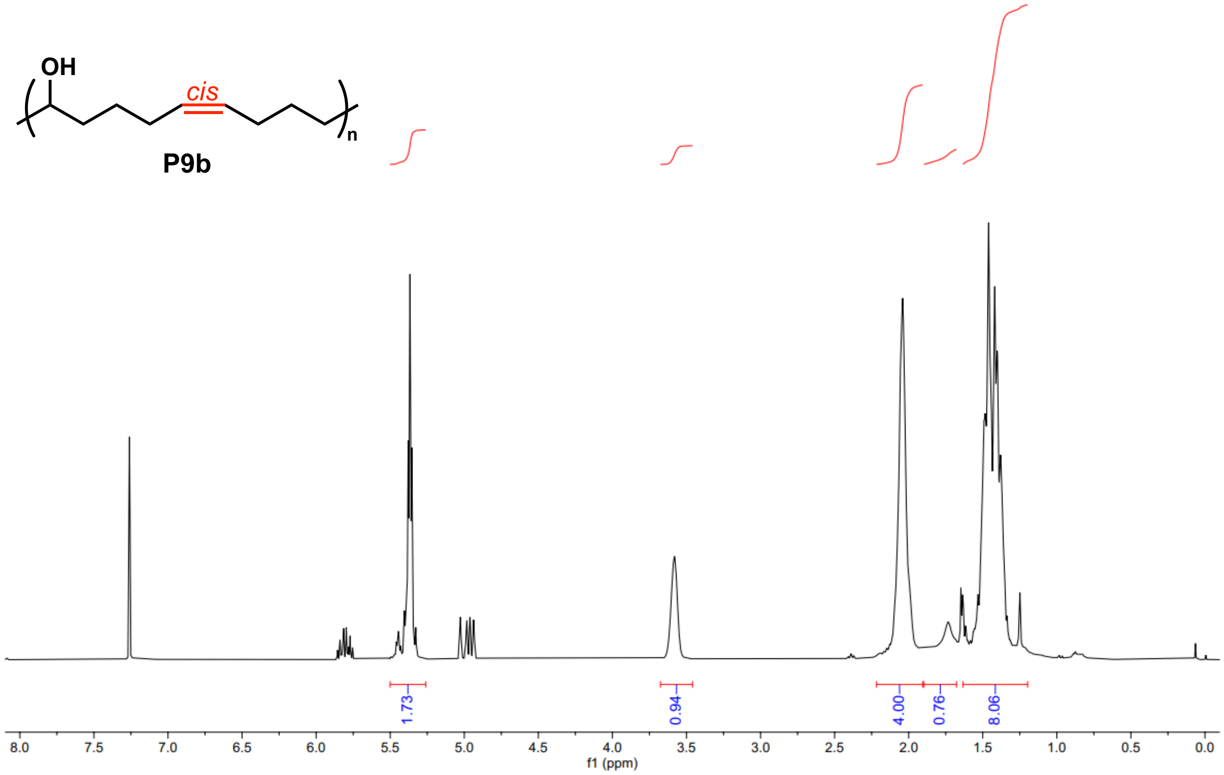

Figure S106. ^{1}H NMR (400 MHz, $CDCl_3$) spectrum of *cis*-rich compound **P9b**.

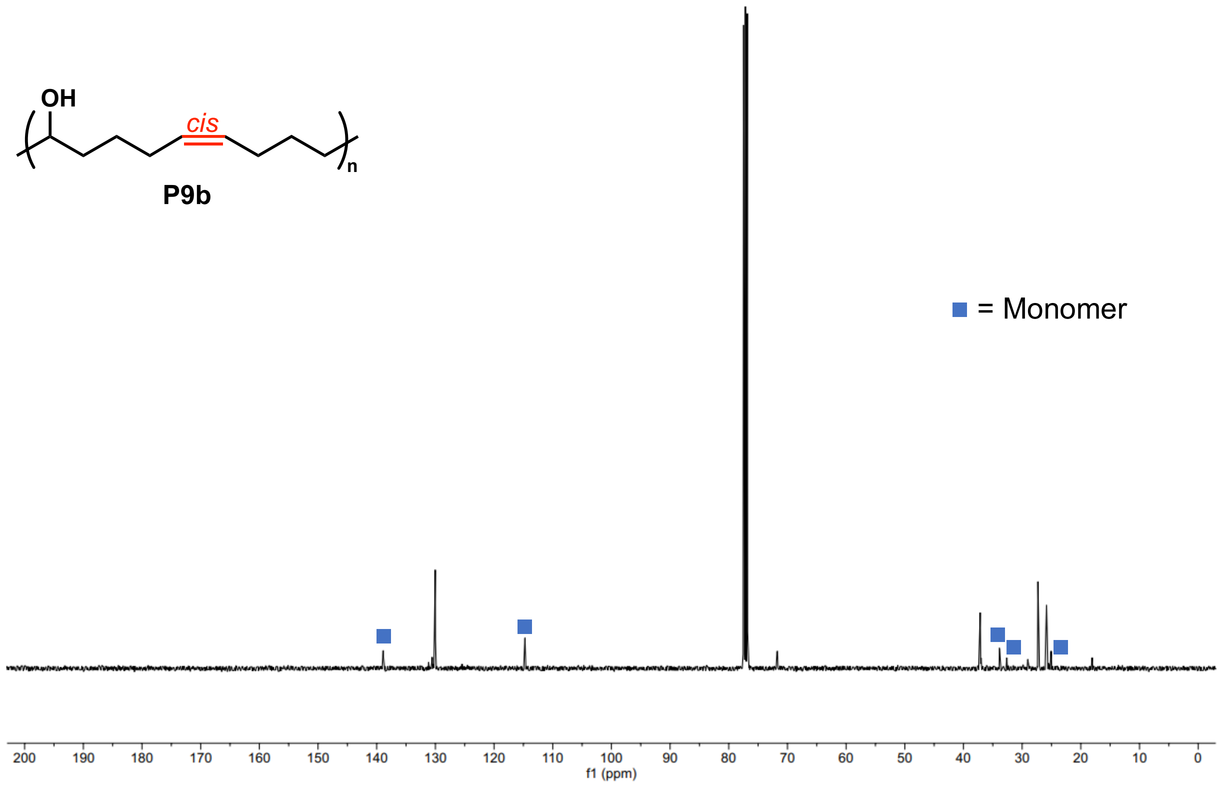

Figure S107. ^{13}C NMR (101 MHz, $CDCl_3$) spectrum of *trans*-rich **P9b** (NOTE: oligomer **P9a** could not be precipitated and separated from unreacted monomer **9a** (blue square labels).

References:

1. Hsu, T.-W.; Kempel, S. J.; Felix Thayne, A. P.; Michaudel, Q., Stereocontrolled acyclic diene metathesis polymerization. *Nat. Chem.* **2023**, *15*, 14–20.
2. Dumas, A.; Tarrieu, R.; Vives, T.; Roisnel, T.; Dorcet, V.; Baslé, O.; Mauduit, M., A Versatile and Highly *Z*-Selective Olefin Metathesis Ruthenium Catalyst Based on a Readily Accessible *N*-Heterocyclic Carbene. *ACS Catal.* **2018**, *8*, 3257–3262.
3. Oliver, W. C.; Pharr, G. M., An improved technique for determining hardness and elastic modulus using load and displacement sensing indentation experiments. *J. Mater. Res.* **1992**, *7*, 1564–1583.
4. Wagener, K. B.; Patton, J. T., Acyclic diene metathesis (ADMET) polymerization. Synthesis of unsaturated polycarbonates. *Macromolecules* **1993**, *26*, 249–253.
5. Patton, J. T.; Boncella, J. M.; Wagener, K. B., Acyclic diene metathesis (ADMET) polymerization: the synthesis of unsaturated polyesters. *Macromolecules* **1992**, *25*, 3862–3867.
6. Poth, D.; Wollenberg, K. C.; Vences, M.; Schulz, S., Volatile Amphibian Pheromones: Macrolides from Mantellid Frogs from Madagascar. *Angew. Chem. Int. Ed.* **2012**, *51*, 2187–2190.
7. Wagener, K. B.; Brzezinska, K., Acyclic diene metathesis (ADMET) polymerization: synthesis of unsaturated polyethers. *Macromolecules* **1991**, *24*, 5273–5277.

8. Boz, E.; Nemeth, A. J.; Ghiviriga, I.; Jeon, K.; Alamo, R. G.; Wagener, K. B., Precision Ethylene/Vinyl Chloride Polymers via Condensation Polymerization. *Macromolecules* **2007**, *40*, 6545–6551.
9. Boz, E.; Nemeth, A. J.; Alamo, R. G.; Wagener, K. B., Precision Ethylene/Vinyl Bromide Polymers. *Adv. Synth. Catal.* **2007**, *349*, 137–141.
10. Valenti, D. J.; Wagener, K. B., Direct Synthesis of Well-Defined Alcohol-Functionalized Polymers via Acyclic Diene Metathesis (ADMET) Polymerization. *Macromolecules* **1998**, *31*, 2764–2773.
11. Gaines, T. W.; Nakano, T.; Chujo, Y.; Trigg, E. B.; Winey, K. I.; Wagener, K. B., Precise Sulfite Functionalization of Polyolefins via ADMET Polymerization. *ACS Macro Lett.* **2015**, *4*, 624–627.
12. Flook, M.M.; Jiang, A.J.; Schrock, R.R.; Müller, P.; Hoveyda, A.H. *J. Am. Chem. Soc.* **2009**, *131*, 7962.
13. Wagener, K. B.; Boncella, J. M.; Nel, J. G., Acyclic diene metathesis (ADMET) polymerization. *Macromolecules* **1991**, *24*, 2649–2657.
